# Adhesion G protein-coupled receptors

**DOI:** 10.1016/j.pharmr.2026.100116

**Published:** 2026-01-16

**Authors:** Tobias Langenhan, Garret R. Anderson, Demet Araç, Gabriela Aust, Monserrat Avila-Zozaya, Sofie Morsing Bagger, Patrick Barth, Sandra Berndt, Stephen C. Blacklow, Beatriz Blanco-Redondo, Antony A. Boucard, James P. Bridges, Lara-Sophie Brodmerkel, Kathleen M. Caron, Yin Kwan Chung, Andrew N. Dates, Virginea de Araujo Farias, Daniel Del Toro, Joseph G. Duman, Felix B. Engel, David M. Favara, Caroline J. Formstone, Chaoyu Fu, Alain Garcia De Las Bayonas, Anastasia Georgiadi, David E. Gloriam, Randy A. Hall, Jörg Hamann, Peter W. Hildebrand, Cheng-Chih Hsiao, Bill X. Huang, Jonathan A. Javitch, Hee-Yong Kim, Robert J. Kittel, Gunnar Kleinau, Richard Leduc, Ines Liebscher, Hsi-Hsien Lin, Joshua Linnert, Marie-Gabrielle Ludwig, David C. Martinelli, Signe Mathiasen, Daniel Matúš, Mariam Melkumyan, Ana L. Moreno-Salinas, Jan Mulder, Michael A. Nash, Kasturi Pal, Daniel T. Pederick, Nicole A. Perry-Hauser, Xianhua Piao, Yu-Qi Ping, Dimitris G. Placantonakis, Fabian Pohl, Simone Prömel, Mette M. Rosenkilde, Laurent Sabbagh, Richard C. Sando, Patrick Scheerer, Torsten Schöneberg, Elena Seiradake, Mareike Selcho, Florian Seufert, Abhishek K. Singh, Georgios Skiniotis, Katja Spiess, Norbert Sträter, David Strutt, Thomas C. Südhof, Jinpeng Sun, Gregory G. Tall, Doreen Thor, Douglas G. Tilley, Kimberley F. Tolias, Mario Vallon, Erwin G. Van Meir, Benoit Vanhollebeke, Giselle R. Wiggin, Uwe Wolfrum, Jie Yan, Nathan A. Zaidman, Yimin Zou, Nicole Scholz

**Affiliations:** 1 Rudolf Schönheimer Institute of Biochemistry, General Biochemistry, Medical Faculty, Leipzig University, Leipzig, Germany; 2 Comprehensive Cancer Center Central Germany (CCCG), Leipzig, Germany; 3 Faculty of Life Sciences, Institute of Biology, Leipzig University, Leipzig, Germany; 4 Department of Molecular, Cell, and Systems Biology, University of California Riverside, Riverside, California; 5 Department of Biochemistry and Molecular Biology, University of Chicago, Chicago, Illinois; 6 Research Laboratories, Department of Orthopedics, Trauma and Plastic Surgery, Medical Faculty, Leipzig University, Leipzig, Germany; 7 Research Laboratories, Department of Visceral, Transplantation, Vascular and Thoracic Surgery, Medical Faculty, Leipzig University, Leipzig, Germany; 8 Department of Cell Biology and Physiology, University of North Carolina at Chapel Hill, Chapel Hill, North Carolina; 9 Department of Biomedical Sciences, Faculty of Health and Medical Sciences, University of Copenhagen, Copenhagen, Denmark; 10 Interfaculty Institute of Bioengineering and Ludwig Institute for Cancer Research Lausanne, École Polytechnique Fédérale de Lausanne, Lausanne, Switzerland; 11 Rudolf Schönheimer Institute of Biochemistry, Signal Transduction, Medical Faculty, Leipzig University, Leipzig, Germany; 12 Department of Translational Drug Research and Signal Transduction, Center for Biotechnology and Biomedicine, Leipzig University, Leipzig, Germany; 13 Department of Biological Chemistry and Molecular Pharmacology, Blavatnik Institute, Harvard Medical School, Boston, Massachusetts; 14 Department of Cell Biology, Centro de Investigación y de Estudios Avanzados del Instituto Politécnico Nacional (Cinvestav), Mexico City, Mexico; 15 Department of Medicine, Division of Pulmonary, Critical Care, and Sleep Medicine, National Jewish Health, Denver, Colorado; 16 Department of Medicine, Division of Pulmonary Sciences and Critical Care, University of Colorado Anschutz Medical Campus, Aurora, Colorado; 17 Department of Psychiatry and Behavioral Sciences, University of California San Francisco, San Francisco, California; 18 Laboratory of Molecular Neurooncology, Department of Neurosurgery, O’Neal Comprehensive Cancer Center, Heersink School of Medicine, University of Alabama at Birmingham, Birmingham, Alabama; 19 Department of Biomedical Sciences, Institute of Neurosciences (IDIBAPS), University of Barcelona, Barcelona, Spain; 20 Department of Neuroscience, Baylor College of Medicine, One Baylor Plaza, Houston, Texas; 21 Department of Nephropathology, Institute of Pathology, Friedrich-Alexander-Universität Erlangen-Nürnberg, Erlangen, Germany; 22 Department of Cardiology, Friedrich-Alexander-Universität Erlangen-Nürnberg, Erlangen, Germany; 23 Division of Structural Studies, MRC Laboratory of Molecular Biology, Cambridge, UK; 24 Department of Oncology, University of Cambridge, Cambridge, UK; 25 Department of Oncology, Addenbrooke’s Hospital, Cambridge University Hospitals NHS Foundation Trust, Cambridge, UK; 26 Department of Clinical, Pharmaceutical and Biological Sciences, University of Hertfordshire, Hatfield, UK; 27 Department of Physics and Mechanobiology Institute, National University of Singapore, Singapore, Singapore; 28 Department of Molecular and Cell Biology, University of California, Berkeley, California; 29 Howard Hughes Medical Institute, Chevy Chase, Maryland; 30 Institute for Diabetes and Cancer, Helmholtz Centre Munich, Neuherberg, Germany; 31 Deutsches Zentrum für Diabetesforschung, Neuherberg, Germany; 32 Department of Drug Design and Pharmacology, University of Copenhagen, Copenhagen, Denmark; 33 Department of Pharmacology and Chemical Biology, Emory University School of Medicine, Atlanta, Georgia; 34 Department of Experimental Immunology, Amsterdam Institute of Immunology and Infectious Diseases, Amsterdam University Medical Center, Amsterdam, the Netherlands; 35 Neuroimmunology Research Group, Netherlands Institute for Neuroscience, Amsterdam, the Netherlands; 36 Institute for Medical Physics and Biophysics, Medical Faculty, Leipzig University, Leipzig, Germany; 37 Laboratory of Molecular Signaling, National Institute on Alcohol Abuse and Alcoholism, National Institutes of Health, Rockville, Maryland; 38 Departments of Psychiatry and Molecular Pharmacology and Therapeutics, Columbia University Vagelos College of Physicians and Surgeons, New York, New York; 39 Division of Molecular Therapeutics, New York State Psychiatric Institute, New York, New York; 40 Department of Animal Physiology, Institute of Biology, Faculty of Life Sciences, Leipzig University, Leipzig, Germany; 41 Charité - Universitätsmedizin Berlin, corporate member of the Freie Univerität Berlin, Humboldt-Universität zu Berlin, Institute of Medical Physics and Biophysics, Group Structural Biology of Cellular Signaling, Berlin, Germany; 42 Department of Pharmacology-Physiology, Université de Sherbrooke, Sherbrooke, Québec, Canada; 43 Department of Microbiology and Immunology, College of Medicine, Chang Gung University, Taoyuan City, Taiwan; 44 Institute of Molecular Physiology (imP), Johannes Gutenberg University Mainz, Mainz, Germany; 45 Biomedical Research, Novartis Pharma AG, Basel, Switzerland; 46 University of Connecticut Health, Farmington, Connecticut; 47 Department of Molecular and Cellular Physiology, Stanford University School of Medicine, Stanford, California; 48 Department of Neuroscience, Karolinska Institute, Stockholm, Sweden; 49 Department of Protein Science, Science for Life Laboratory, KTH Royal Institute of Technology, Stockholm, Sweden; 50 Department of Chemistry, Institute of Physical Chemistry, University of Basel, Basel, Switzerland; 51 Department of Biosystems Sciences and Engineering, ETH Zurich, Basel, Switzerland; 52 Department of Biology, Ashoka University, Sonipat, India; 53 Department of Neuroscience, School of Medicine, Johns Hopkins University, Baltimore, Maryland; 54 Centre for Translational Pharmacology, School of Molecular Biosciences, University of Glasgow, Glasgow, UK; 55 Weill Institute for Neuroscience, University of California San Francisco, San Francisco, California; 56 Medical Science and Technology Innovation Center, Shandong First Medical University & Shandong Academy of Medical Sciences, Jinan, China; 57 Departments of Neurosurgery and Cell Biology, NYU Grossman School of Medicine, New York, New York; 58 Institute of Cell Biology, Heinrich Heine University Düsseldorf, Düsseldorf, Germany; 59 Domain Therapeutics, St-Laurent, Quebec, Canada; 60 Department of Pharmacology, Vanderbilt Brain Institute, Vanderbilt University, Nashville, Tennessee; 61 Rudolf Schönheimer Institute of Biochemistry, Molecular Biochemistry, Medical Faculty, Leipzig University, Leipzig, Germany; 62 University of Global Health Equity (UGHE), Kigali, Rwanda; 63 Department of Biochemistry, University of Oxford, Oxford; 64 Kavli Institute for Nanoscience Discovery, University of Oxford, Oxford; 65 Department of Structural Biology and Center of Excellence for Structural Cell Biology, St. Jude Children’s Research Hospital, Memphis, Tennessee; 66 Department of Virology and Microbiological Preparedness, Statens Serum Institut, Copenhagen, Denmark; 67 Institute of Bioanalytical Chemistry, Center for Biotechnology and Biomedicine, Leipzig University, Leipzig, Germany; 68 School of Biosciences, University of Sheffield, Sheffield; 69 Howard Hughes Medical Institute, Stanford University School of Medicine, Stanford, California; 70 Advanced Medical Research Institute, Cheeloo College of Medicine, Shandong University, Jinan, China; 71 Department of Pharmacology, University of Michigan Medical School, Ann Arbor, Michigan; 72 Department of Cardiovascular Sciences, Aging and Cardiovascular Discovery Center, Lewis Katz School of Medicine, Temple University, Philadelphia, Pennsylvania; 73 Institute of Anatomy and Cell Biology, University of Würzburg, Würzburg, Germany; 74 Laboratory of Neurovascular Signaling, Department of Molecular Biology, ULB Neuroscience Institute, Université libre de Bruxelles, Gosselies, Belgium; 75 WEL Research Institute, Wavre, Belgium; 76 Nxera Pharma UK Ltd., Cambridge, UK; 77 Institute for Quantitative and Computational Biosciences (IQCB), Johannes Gutenberg University Mainz, Mainz, Germany; 78 Department of Biochemistry and Molecular Biology, University of New Mexico School of Medicine, Albuquerque, New Mexico; 79 Neurobiology Section, Biological Sciences Division, University of California, San Diego, La Jolla, California

## Abstract

Adhesion G protein–coupled receptors (aGPCRs) constitute a structurally and functionally distinct group within the superfamily of GPCRs. In 2015, the International Union of Pharmacology invited the Adhesion GPCR Consortium to publish a comprehensive review about aGPCRs and establish a unified nomenclature. Since then, substantial progress has been made in delineating the biological roles, molecular architecture, biochemical properties, expression profiles, ligand repertoire, and activation and signaling strategies of aGPCRs. Commensurate with these advances, their relevance to human pathophysiology has become increasingly apparent. In a coordinated effort, the Adhesion GPCR Consortium has reviewed recent progress in this field and provides a comprehensive assessment of the current understanding of aGPCR biology, including a focus on human and mammalian aGPCRs, their evolutionary origins, methodological approaches, and model systems for their investigation, as well as emerging approaches for their therapeutic targeting.

**Significance Statement:**

Adhesion G protein–coupled receptors are versatile cell-surface proteins that integrate structural, biochemical, and physiological functions, with major roles in health and disease. This review summarizes current knowledge of their molecular features, functions in diverse model systems, and emerging opportunities for therapeutic targeting, providing a comprehensive resource that connects basic biology with translational applications across multiple scientific disciplines.

## I Introduction

Adhesion G protein–coupled receptors (aGPCRs) form a remarkable set of molecules within the superfamily of GPCR.^1^ Ever since aGPCRs were recognized as a receptor family^2^ and alternatively classified as B2 GPCRs,^3^ researchers across many scientific research fields have focused on elucidating their molecular architecture, biochemical features, pharmacological properties, physiological functions, and involvement in disease. This review summarizes the current state of knowledge on aGPCRs, covering a broad range of aspects relevant to the various scientific communities interested in these receptors.

In the following sections, we present an introduction to the general molecular, cellular, and physiological characteristics of all aGPCRs, with an emphasis on human and other mammalian family members. Furthermore, this review offers an overview of current efforts to elucidate the role of aGPCR dysfunction in various diseases and explores strategies for their pharmacological targeting. Finally, we provide a survey of the principal model organisms and experimental approaches currently employed in aGPCR research.

## II Receptor terminology

**Fig. 1:**
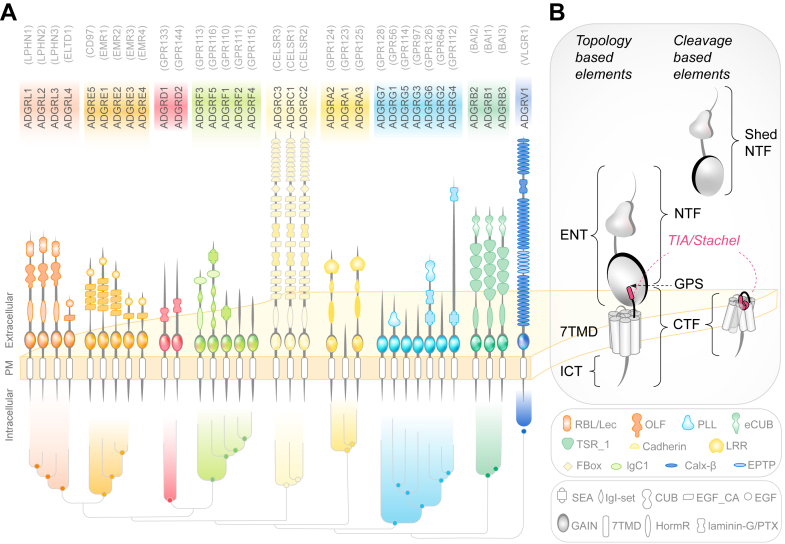
(A) Overview of human orthologs of the aGPCR family, their domain architecture, and phylogenetic relationship.^7^ Receptor names conform to current IUPHAR nomenclature^1^; previous names are given above in brackets. Domains are not drawn to scale, domain numbers per ortholog can vary depending on splice variant/isoform; the phylogenetic relationship is not shown for E4 due to its pseudogene status. NCBI’s Conserved Domain Database^8^ and Expasy PROSITE^9^ were used to annotate domains. Additionally, models were generated using AlphaFold2^10^ (except for V1) to corroborate the sequence-based with a structure-based approach. In case of a mismatch, FoldSeek^11^ was used to identify similar, known domains, based on its predicted structure. Where available (see Sections IV.A and B), known structures of aGPCR ENTs were used to verify predicted domains. Different types of Ig domains were assigned according to their specific topology.^12^ A laminin EGF-like domain was predicted N-terminal to the GAIN domain of all C receptors but was absent in the cryo-EM structure of ADGRC1^13^ and was thus omitted from the sketches. Legend: domain shapes only found in 1 subfamily are color-filled, and domains found in more than 1 subfamily are left blank. (B) Schematic depiction of receptor elements of aGPCR based either on their topology (left) applicable to noncleaved and self-cleaved receptors or on autoproteolysis (right) applicable to self-cleaved receptors only. Autoproteolyzed aGPCRs can exist as nondissociated or dissociated noncovalent NTF-CTF complexes. TIA/Stachel is indicated in pink. 7TMD, heptahelical transmembrane domain; CA, cadherin; Calx-*β*, Na-Ca exchanger *β*; CTF, C-terminal fragment; CUB, complement C1r/C1s, Uegf, Bmp1; eCUB, extended complement C1r/C1s, Uegf, Bmp1; EGF, epidermal growth factor; ENT, extracellular N-terminus; EPTP, epitempin; GAIN, GPCR autoproteolysis-inducing; GPS, GPCR proteolysis site; HormR, hormone receptor motif; IgI-set, immunoglobulin I-set; ICT, intracellular C-terminus; IgC1, immunoglobulin C1-set; LLR, leucine-rich repeat; NTF, N-terminal fragment; OLF, olfactomedin; PLL, Pentraxin/Laminin/neurexin/sex-hormone-binding-globulin-like; PM, plasma membrane; PTX, pentraxin; RBL/LEC, rhamnose-binding lectin/lectin; SEA, Sea urchin sperm protein, Enterokinase, Agrin; TIA, tethered/intramolecular agonist; TSR, thrombospondin repeat. See section II.

**Fig. 2:**
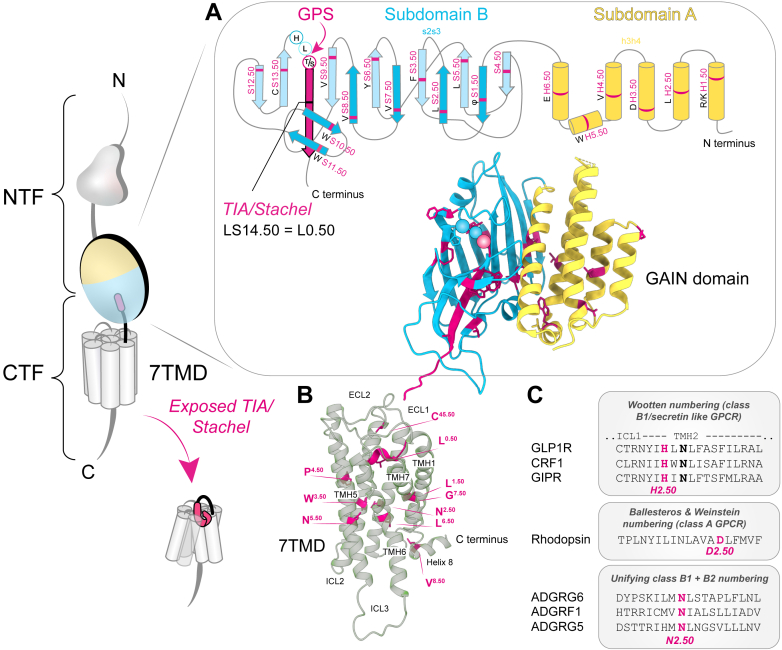
Generic residue numbering schemes for the GAIN domain and 7TMD of aGPCRs. Generic residue numbers indicate the position of a specific AA within a secondary structural element relative to the most conserved residue of the respective segment, which is assigned the number “X.50.” (A) The GAIN domain, located N-terminal to the 7TMD, is subdivided into subdomain A (yellow), which contains several *α*-helices (TMH1–TMH6), and subdomain B (blue), which consists of *β*-strands (S1–S14) and the GPS (red circles). S14 corresponds to the *β*-strand formed by the *Stachel* core in its in GAIN-bound conformation.^14^ Disordered regions connecting structural elements are labeled using the abbreviations of the adjacent segments, such as h3h4 or s2s3. The schemes in (A) represent structural models of aGPCR fragments determined through analysis of GAIN subdomain A (UniprotKB: A0A2Y9F628) and GAIN subdomain B (UniprotKB: A0A2I4CCH8). (B) The 7TMD is composed of 8 *α*-helices (TMH1-7 and helix 8), each with highly conserved AA residues characteristic of class B GPCRs,^15^ visualized at the CTF structure of F1 (PDB ID 7wu5). The TIA/*Stachel* core with the highly conserved L0.50 as part of the CTF can be bound as a helical segment in the 7TMD. The conserved ECL2 residues C^45^^,^^50^ and V8.50 in the cytosolic helix 8 are included in supposed class B GRNs.^16^ (C) Example of a comparison between different GPCR numbering schemes for TMH2. See section IV.A, C.

**Fig. 3:**
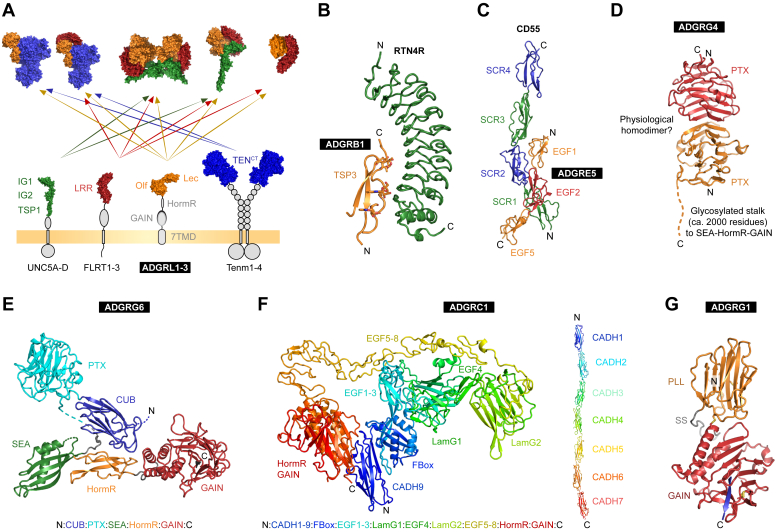
Extracellular domains of aGPCRs and their interactions. (A) Experimentally shown and predicted extracellular complex formations of L1-3 LEC and OLF domains (orange).^17^, ^18^, ^19^, ^20^, ^21^, ^22^, ^23^ (B) Interaction of the TSR3 domain of B1 with RTN4R. Mannose and fucose residues at the binding interface are shown as sticks. (C) Crystal structure of the E5–CD55 complex. (D) The G4 pentraxin domain forms a homodimer. (E) The N-terminal CUB domain of the ENT of G6 interacts with the HormR-GAIN domains resulting in a compact ENT structure. (F) Cryo-EM structure of CADH9-GAIN part (left) and crystal structures of the N-terminal cadherin domains 1–7 (right) of the C1 ENT. (G) Crystal structure of the ENT of G1. A disulfide bridge (SS) links the PLL and GAIN domains. See section IV.B.

Similar to other GPCRs, the structural organization of aGPCRs can be topologically subdivided into the following 3 main regions: an extracellular N-terminus (ENT), a 7-transmembrane helix domain (7TMD) including extra- (ECL) and intracellular loops (ICLs), and an intracellular C-terminus (ICT) (Figs. 1 and 2).^1^^,^^4^, ^5^, ^6^, ^7^, ^8^, ^9^, ^10^, ^11^, ^12^, ^13^, ^14^, ^15^, ^16^ The ENTs of aGPCRs frequently contain a variety of protein domains commonly associated with adhesive functions (Fig. 3).^17^, ^18^, ^19^, ^20^, ^21^, ^22^, ^23^ This structural complexity can contribute to the unusually large size of many aGPCRs and underlies the substantial structural and functional diversity of this receptor class. Although the ICTs of aGPCRs can also be very large, no annotated domains have been identified to date. Nevertheless, ICTs can facilitate interactions through PDZ (PSD95/Dlg1/ZO-1)-binding motifs (PBMs) to PDZ domains of scaffold proteins.^24^

**Fig. 4:**
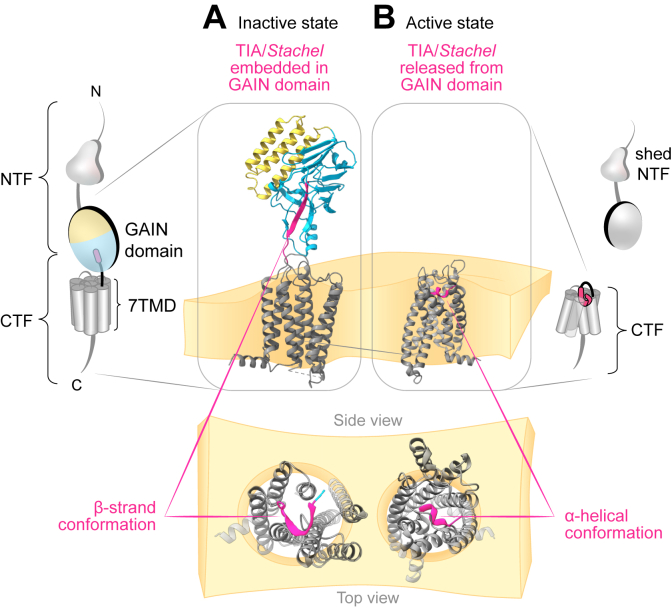
Adhesion GPCR activation mediated by the TIA/*Stachel*. The illustration exemplifies the structural transition of an aGPCR from its inactive to active state using structures of E5.^27^ (A) In the inactive conformation (PDB ID: 8IKJ), the TIA (in pink) is embedded in the GAIN domain as a buried *β*-strand. (B) Upon activation, the NTF dissociates from the CTF exposing the TIA. The TIA then inserts into the orthosteric binding pocket of the 7TMD through its N-terminal *α*-helical segment (PDB ID: 8IKL). See section IV.C.

The GPCR autoproteolysis-inducing (GAIN) domain is the only extracellular structural element consistently found across nearly all aGPCRs (Figs. 1A and 2A),^25^ with the exception of Eutherian A1 orthologs.^14^ Located just outside the membrane, the GAIN domain of many, but not all, aGPCRs^16^ can mediate receptor autoproteolysis at its intradomain GPCR proteolysis site (GPS),^25^^,^^26^ resulting in the cleavage of many, albeit not all, aGPCRs into N-terminal (NTF) and C-terminal (CTF) fragments (Fig. 4).^27^ These fragments remain noncovalently associated at the cell surface,^28^, ^29^, ^30^ where they can dissociate spontaneously and in response to ligand binding combined with mechanical stimuli. The structural entity of the GAIN domain–cleaved aGPCRs may be termed NTF-CTF complex rather than NTF-CTF dimers or heterodimers to avoid confusion with GPCRs that form functional oligomeric assemblies of more than 1 receptor molecule, for example, GABA_B_ or metabotropic glutamate receptors.^31^

The NTFs of GAIN domain–cleaved aGPCRs comprise various extracellular domains, including the larger part of the cleaved GAIN domain (Fig. 3), whereas the CTF includes the C-terminal *β*-strand of the GAIN domain, the linker connecting the GAIN domain and 7TMD, the full 7TMD (Fig. 4), and the complete ICT. The segment corresponding to the last *β*-strand of the GAIN domain, which forms the N-terminus of the CTF, can undergo structural changes to engage directly with its own 7TMD and activate the receptor. Accordingly, this segment is referred to as a tethered or intramolecular agonist, also known as the *Stachel* (German for “*stinger*”; Fig. 2A). The *Stachel* sequence of aGPCRs does not represent a separate “tethered” ligand but an integral segment of each receptor’s polypeptide chain. Peptide mapping established its hydrophobic core as sufficient for receptor activation; however, unlike covalently bound chromophores (eg, retinal in opsins), the *Stachel* is encoded, synthesized, and retained within the same chain.^32^^,^^33^ Consequently, the *Stachel* functions as an intramolecular agonist and may also be termed as such.^16^ In the course of this review, the agonist is referred to by the compound abbreviation tethered/intramolecular agonist (TIA) or by the term *Stachel*.

The stretch of amino acid (AA) residues linking the extracellular domains N-terminal to the GPS motif was historically denominated as a “stalk.”^34^^,^^35^ It was later shown that the “stalk” and “GPS motif” are constituent sequences of the GAIN domain.^25^ Thus, the term “stalk” has become superfluous, and its use should be discontinued.^36^ Of note, the coordinates of the “stalk” region are distinct from the TIA, which immediately follows C-terminal to the GPS.^16^

Since the adoption of the revised nomenclature system for aGPCRs in 2015 by the Adhesion GPCR Consortium, Nomenclature and Standards Committee of the International Union of Basic and Clinical Pharmacology (NC-IUPHAR), and Human Genome Organisation Gene Nomenclature Committee (HGNC), the updated gene/protein names using the adhesion G protein-coupled receptor (ADGR) prefix have been successfully integrated into scientific communication.^1^ We suggest using these new names alongside their traditional synonyms, for example, “ADGRB1/BAI1” or “ADGRL3/Latrophilin-3,” at least at the first mention in a publication. Once introduced, shorthand versions of the receptor names (eg, B1 or L3) may be used, as conducted throughout this review.

## III Generic residue numbering

Generic residue numbering (GRN) schemes enable the comparison of corresponding AA residue positions in homologous proteins within or between species. The common principles for generating GRNs are based on either (1) identifying the most highly conserved AA positions in aligned protein sequences, which allows for assigning reference residues or (2) identifying structural alignments (superimpositions) that consider secondary or even tertiary structures within conserved folds to define corresponding AA positions. Such comparisons can be critical to infer function, establish structure-function relationships across diverse datasets (eg, conserved sequence motifs, structural interactions, and pharmacologically and pathologically relevant mutations), or facilitate a more consistent understanding of evolution.

Most GPCRs, including aGPCRs, share an archetypal 7TMD (Fig. 4) with an ENT and an ICT. The complex, often multidomain structure of the aGPCR ENT varies across receptor subtypes, even within the same receptor group (Figs. 1 and 3). As a result, a common GRN scheme for the N-termini of different aGPCR groups is not feasible. Furthermore, multiple transcript variants of individual members^37^ add further complexity to the numbering system. This also applies to the GAIN domain, found in 31 human aGPCRs (not considering *E4* due to its pseudogene status). The protein sequences of the GAIN domain vary substantially among aGPCR members, making reliable sequence alignment for assigning reference residues extremely difficult.^14^^,^^16^

The *Stachel* core sequence and the GPS motif comprise the first highly conserved extracellular AA sequences directly adjacent to transmembrane helix (TMH) 1. The *Stachel* core has been observed as a *β*-strand bound within GAIN domain or ENT structures (Fig. 2A) but adopts an *α*-helical structure within the 7TMD in active state-like CTF structures (Fig. 2B). A recent sequence-based numbering approach focused on this conserved extracellular *Stachel* core sequence^32^^,^^33^ and identified a leucine (L0.50) within the TxFxxLM core motif as the most highly conserved residue.^16^ Here, “0” indicates the CTF region, although highly conserved TMH residues are denoted by the respective helix number (1, 2, etc.) combined with the “.50” designation (eg, L1.50), allowing for up- or down-counting of adjacent residues. This scheme is an adaptation of the Ballesteros-Weinstein numbering system for class A GPCR.^38^

Due to the high sequence variability of the GAIN domain, an alternative GRN scheme was developed based on the structural comparison of 14,435 vertebrate GAIN domain models.^14^ This analysis revealed a secondary structure consensus of 6 *α*-helices (H1–H6) in the more structurally variable subdomain A (Fig. 2A), where some aGPCR subfamilies, for example, D and G, show heterogeneity ranging from 2 to 6 *α*-helices. In the more conserved subdomain B, 14 *β*-strands (S1–S14) were defined, with the GPS motif individually indexed (Fig. 2A). In this GRN scheme, the *Stachel* is designated as S14 (strand 14 of subdomain B), with its most highly conserved residue LS14.50 (equivalent to L0.50) located in the *Stachel* core.

For the 7TMD, the Wootten numbering scheme^39^ was originally developed for the secretin receptor-like family (class B1 GPCRs) based on sequence conservation analyses and was later also applied to aGPCRs (class B2 GPCRs) (Fig. 2C). This scheme was subsequently extended to include ICL1, ECL2, and helix 8, and the TMH1-7 numbering was revised to incorporate structural data that account for helix bulges or constrictions.^40^^,^^41^ These GRNs were integrated into the GPCR database, alongside the GAIN GRN scheme.^14^^,^^42^^,^^43^

Importantly, phylogenetic analyses revealed that secretin-like receptors evolved from aGPCRs.^7^^,^^44^^,^^45^ Consequently, a unified GRN scheme for most of the CTF region (*Stachel*-7TMD-intracellular helix 8), based on high sequence conservation across both aGPCR and secretin-like receptors, was recently proposed (Fig. 2C).^15^ This provides a standardized framework for residue comparison across diverse receptor groups and members.

## IV Structures of adhesion G protein–coupled receptors

### A GPCR autoproteolysis-inducing domains

Crystal structure analyses of the GAIN domains of human B3 and rat L1^8^ showed that the previously identified GPS motif,^26^ which comprises roughly 40–60 residues and includes several highly conserved residues,^46^ is located at the C-terminus of a larger fold comprising approximately 320 residues and 2 subdomains (Figs. 3 and 4).

The N-terminal subdomain A consists of up to 6 *α*-helices, with 5 of them forming a roughly parallel bundle, whereas a shorter helix 5 typically sits perpendicular to the others like a lid on top. Subdomain A is sometimes short through the lack of several *α*-helices (section III; Fig. 4A), for example, in mouse G1,^47^ human G3,^48^ or human E5.^27^

The core fold of the C-terminal subdomain B consists of a twisted *β*-sandwich, including 11 *β*-strands in 2 antiparallel *β*-sheets. Additionally, short *α*-helices or *β*-strands may be found within flexible loop regions. Two highly conserved disulfide bridges stabilize subdomain B, both anchoring with one end close to the expected cleavage site (CxCxHL|T).

The cleavage site itself (referred to as GPS) is located within a sharply bent loop right before the last *β*-strand, which corresponds to the *Stachel*^32^^,^^33^ (Fig. 4A). The GPS is flanked by 2 flexible regions, dubbed Flap 1 and Flap 2, which influence solvent accessibility and exposure of the GPS.^30^ Structures of human F1,^49^ G3,^48^ L3,^50^ mouse G1,^47^ zebrafish G6,^51^ and rat L1^8^ in the cleaved state demonstrated that the *Stachel* remains tightly bound within the GAIN domain fold after self-cleavage. The GAIN domains of human B2,^52^ B3,^25^ and a cleavage-deficient mutant of E5^35^ were observed in an uncleaved state. To date, no receptor has been structurally characterized in both a precleavage and cleaved state.

### B Extracellular domains and ligand-bound structures

Adhesion GPCRs exhibit diverse architectures of the ENTs that are critical for their unique activation mechanisms and functional specificity. Although the membrane-proximal GAIN domain is present in most and the hormone receptor motif (HormR) domain in many aGPCRs, a diverse set of more N-terminal domains form a plethora of structures and interaction surfaces for binding partners of each individual aGPCR. Recent studies using mainly X-ray crystallography and single-particle cryogenic electron microscopy (cryo-EM) have begun to uncover the structural diversity of these domains and their complexes. Although each structure appears unique, there are commonalities in how aGPCRs use their ENTs to recognize ligands (Table 1).^53^, ^54^, ^55^, ^56^, ^57^, ^58^, ^59^, ^60^, ^61^, ^62^, ^63^, ^64^, ^65^, ^66^, ^67^, ^68^, ^69^, ^70^, ^71^, ^72^, ^73^, ^74^, ^75^, ^76^, ^77^, ^78^, ^79^, ^80^, ^81^, ^82^, ^83^, ^84^, ^85^, ^86^, ^87^, ^88^, ^89^, ^90^, ^91^, ^92^, ^93^, ^94^, ^95^, ^96^, ^97^, ^98^, ^99^, ^100^, ^101^, ^102^, ^103^, ^104^, ^105^, ^106^, ^107^, ^108^, ^109^, ^110^, ^111^, ^112^, ^113^, ^114^, ^115^, ^116^, ^117^, ^118^, ^119^, ^120^, ^121^, ^122^, ^123^, ^124^, ^125^, ^126^, ^127^, ^128^, ^129^, ^130^, ^131^, ^132^, ^133^, ^134^, ^135^, ^136^, ^137^, ^138^, ^139^, ^140^, ^141^, ^142^, ^143^, ^144^, ^145^, ^146^, ^147^, ^148^, ^149^, ^150^, ^151^, ^152^, ^153^, ^154^, ^155^, ^156^, ^157^, ^158^

**Table 1:** Identity, level of evidence, effect, and interface of known interaction partners of aGPCRs

Receptor	Endogenous Ligands/Interactors	Exogenous Ligand	Species/Organism of Receptor	Identification/Isolation Method	Tissue of Identification or Tissue/Cell Expression	Signaling Pathways	Interaction Interface	References
Subfamily A								
A2	RECK/Wnt7a/Wnt7b/Dishevelled-2		Mouse Zebrafish		Endothelium	*β*-catenin Cdc42		53, 54, 55, 56, 57, 58, 59, 60
A2	*α*_v_*β*_3_ Integrin, *α*_5_ Integrins		Human	Adhesion assay, affinity chromatography	Human umbilical vein endothelial cells (HUVEC)		ENT, HormR domain RGD motif	^61^
A2	Heparin		Human	ELISA	HUVEC cells		Unknown, but depends on the ionic strength of the environment (1M NaCl)	^61^
A2	Glycosaminoglycans (chondroitin sulfate A, heparan sulfate, dermatan sulfate)		Human	ELISA	HUVEC cells			^61^
A2	SDC1/SDC 2 (Syndecan)		Human	Cell-based genome-wide approach with CRISPR activation	HEK293 cells	-	-	^62^
A2	Discs large		Human	-	-	-	ICT, PDZ-binding motif(PBM)	^63^
Subfamily B								
B1	RTN4s/NoGo receptors (RTN4R, RTN4RL1, RTN4RL2)		Mouse, Human	Cell-based genome-wide approach with CRISPR activation, cell surface labeling assay, quantitative cell adhesion assay, binding affinity measurements surface plasmon resonance, pulldown, mass spectrometry	Neuron/glia, HEK293 cells	-	ENT, TSR3 domain (B1-RTN4R K_d_ = 26.8 nM, B1-RTN4RL1 Kd = 9.6 nM, ENT, B1-RTN4RL2 K_d_ = 30 nM)	^62^^,^^64^^,^^65^
B1	Neuroligin 1		Mouse	Functional Assays, coimmunoprecipitation	Hippocampal neurons, HEK293 cells	-	ENT, Vasculostatin	^66^
B1	Phosphatidylserine		Mouse	Overlay binding assays phagocytosis assay (in vitro), engulfment assay (in vivo)	LR73 Chinese hamster ovary cells, J774 macrophages and NIH 3T3 cells) and primary cells (mouse astrocytes); apoptotic thymocytes injected into mouse peritoneum	ELMO1/Dock/Rac	ENT, TSR	^67^
B1	ATP11A			Coimmunoprecipitation assays	HEK293T cells	Reduces constitutive G*α*_12/13_ signaling	NTF	^68^
B1	Lipopolysaccharide		Mammalian	Biotin-streptavidin-phycoerythrin based flow cytometry	Macrophages	Rac/ELMO/Dock	ENT, TSRs	^69^
B1	Integrin *α*_v_*β*_5_		Mouse	Immunoprecipitation of mouse brain lysate	HUVEC cells	-	ENT	^70^
B1	BAI-associated protein 2/IRSp53		Human	Y2H	-	-	Binding between AAs 1304 - 1584	^71^
B1	BAI-associated protein 3		Human	Coimmunoprecipitation	COS-7 cells	-	ICT	^72^
B2	GIP3 (glutaminase interacting protein)		Human	Human fetal brain cDNA library screened using a yeast 2-hybrid assay, Circular Dichroism, Nuclear Magnetic Resonance spectroscopy	Human brain	G*α*_16_, NFAT pathway	ICT, C-terminal sequence RDGDFQTEV	^73^^,^^74^
B3	C1q-like proteins		Mouse	Affinity chromatography, Mass spectrometry, pulldown, proteomics	Mouse brain samples, HEK293 cells, C2C12 cells (muscle cell line)	-	ENT, eCUB, (C1ql-4) (K_d_ = 1–20 nM)	75, 76, 77, 78
B3	RTN4R and RTN4RL1		Mouse	Cell surface labeling assays, quantitative cell adhesion assay, pulldown	HEK293 cells	-	ENT, TSR2 (B3-RTN4R with binding affinity of K_d_ = 1.9 nM)	^64^^,^^65^
B3	Stabilin-2		Mouse, Chicken	Proteomics	Purified BAI3 HEK293T cells and to affinity purify proteins from the supernatant of differentiating C2C12	ELMO proteins, *β*-Arrestin2	ENT	^78^
B3	Neuronal pentraxins (NPTX1/ NPTXR)		Mouse	Time-controlled cross-linking, Aggregation assay, Coimmunoprecipitation, mass spectrometry	Mouse brain lysates, HEK293 cells		-	^75^
Subfamily C								
C1	ADGRC1 (homophilic *trans*-interaction)		Mouse	Cell binding assay	HEK293 cells	-	ENT, cadherin repeats	^79^
C1	ADGRC2 (heterotypic *trans*-interaction)		Mouse	Cell binding assay	HEK293 cells	-	ENT; cadherin repeats	^79^
C1	Vangl-2		Mouse	Fluorescence-based recruitment assay, coimmunoprecipitation	Cultured keratinocytes, HEK293T cells	-	-	^80^^,^^81^
C1	Frizzled-6		Mouse	Fluorescence-based recruitment assay	Cultured keratinocytes	-	-	^80^
C1	LRRK2		Mouse, Human	Coimmunoprecipitation, mass spectrometry	Substantia nigra cells (SN4741), HEK293T cells	WNT/*β*-catenin pathway	-	^82^
C2	ADGRC2 (homophilic interaction)		Rat	Coculture and aggregation assays, Calcium imaging	Cortex and hippocampal neurons, HEK293 cells	CaMKII, calcineurin, Ca^2+^ signaling	ENT, cadherin repeats	^79^^,^^83^
C2	ADGRC1 (homophilic *trans*-interaction)			Cell binding assay	HEK293 cells	-	ENT, cadherin repeats	^79^
C3	ADGRC3 (homophilic *trans*-interaction)		Rat	Cell binding assay, Co-IP (exogenous and brain lysate), aggregation assays, Calcium imaging	Cortex and hippocampal neurons, HEK293 cells	Ca^2+^ signaling	ENT, cadherin repeats	^83^^,^^84^
C3	Dystroglycan		Mouse	Live-cell binding assay, coimmunoprecipitation	COS-7 cells, HEK293 cells		ENT, LG1 domain	^84^
C3	Frizzled-3		Mouse	Coimmunoprecipitation	Brain extracts	-	-	^85^
C3	PSD-95		Mouse	Coimmunoprecipitation	Brain extracts	-	-	^85^
C3	SV2		Mouse	Coimmunoprecipitation	Brain extracts	-	-	^85^
Subfamily D								
D1	Protein Tyrosine Kinase 7 (Ptk7)		Human-patient derived, Mouse	Affinity copurification, mass spectrometry	Patient-derived GBM cells, HEK293T cells	cAMP (specifically in trans), requires membrane anchoring	NTF	^86^^,^^87^
D1	5-hydrotestosterone		Mouse	cryo-EM structure	HEK293 cells	G*α*_s_, cAMP-PKA	7TMD	^88^
D1	Plexin Domain-Containing Protein 2		Human	Library of single-transmembrane-spanning human cell surface receptors followed by interaction screening based on AVEXIS method	Expi293F cells	Increase in cAMP	ENT, PTX domain	^87^
D1	Methenolone		Mouse	cAMP screen, cryo-EM structure	HEK293 cells	G*α*_s_, G*α*o cAMP-PKA	7TMD	^88^
D1		AP503		cryo-EM structure	HEK293 cells	G*α*_s_, cAMP-PKA	7TMD	^88^
Subfamily E								
E2	Dermatan sulfate (chondroitin sulfate B)		Human	Cell-based ligand-binding assay	Most cell surface, ECM	Mast cell degranulation (histamine release)	ENT, EGF-like domain 4	^89^^,^^90^
E2	FHR1		Human	RNA sequencing/Gene associated disease analysis, direct protein-protein interaction assay	Complement system protein in blood	Inflammasome activation in monocytes	ENT	^91^
E3	Unknown surface ligand		Human	Cell-based ligand-binding assay	Macrophages and activated neutrophils	ND	ENT	^92^
E4	Unknown surface ligand		Mouse	Cell-based ligand-binding assay	A20 mouse B-cell lymphoma cell line	ND	ENT, EGF-like domain 2	^93^
E5	CD55		Human/Mouse	Ab blocking assay/biochemical analysis	Red blood cells, lymphocytes, etc.	Induces G*α*_13_ signaling in cDC2 and marginal zone B cells in the spleen	ENT, EGF-like domains 1, 2, 5	94, 95, 96, 97
E5	Dermatan sulfate		Human	Cell-based ligand-binding assay	Most cell surface, ECM	ND	ENT, EGF-like domain 4	^90^^,^^98^
E5	*α*5*β*1, *α*v*β*3 integrin		Human	Functional blocking Abs in cell attachment assay	HUVEC cells	Enhances HUVEC chemotactic migration and angiogenic response	ENT, RGD motif	^99^
E5	CD90		Human	Cell adhesion assay/protein-cell binding assay	Activated endothelial cells, Glioblastoma cells	ND	ENT, GAIN domain	^100^^,^^101^
E5	LPAR1		Human	Co-IP of tagged receptors, in situ proximity ligation assay	Prostate cancer and thyroid cancer cells	Increased Rho-ERK signaling	7TMD	^102^^,^^103^
E5	RIG-I		Mouse	Immunoprecipitation	HEK-293T or HeLa cells	Inhibition of IFN-I signaling	ICT	^104^
E5		Gingipain K protease	Human	Multiplexed bioactivity screening of GPCRs, microbiome-GPCRome interactions	*Porphyromonas gingivalis*	Increased Tango reporter activity	ENT, cleavage at K290 residue	^105^
E5		SteD surface effector	Mouse	Pulldown, mass spectrometry, coimmunoprecipitation	MutuDCs (immortalized dendritic cells)	Ubiquitination of a cytoplasmic lysine residue	CTF	^106^
E5	DLG1		Human	GST-Pulldown, proximity ligation assay	CD97-(over) expressing HT1080 cells; DLD1 cells		ICT, PBM	^107^
Subfamily F								
F1	N-docosahexaenoylethanolamine (synaptamide)		Mouse	Pull down coupled to mass spectrometry	Mouse fetal brains or NSCs, HEK293	G*α*s	ENT, GAIN domain	^108^^,^^109^
F1	Occludin		Mouse	Chemical cross-linking, affinity purification, and mass spectrometry	HEK293	ND	ND	^110^
F1	Laminin-211		Human/Mouse	Cross-linking-aided IP coupled with mass spectrometry	BT474 and SKBR3 cells overexpressing Adgrf1	Inhibition of G*α*_s_	ND	^111^
F5	Surfactant protein D		Mouse	Coimmunoprecipitation	HEK293T			^112^
F5	FNDC4		Mouse	Fluorescent flow cytometry binding assay in live cells	Immortalized pre adipocytes (imm. SVF) from inguinal WAT	G*α*_s_, increase in cAMP		^113^
Subfamily G								
G1	Phosphatidylserine			Flow cytometry, membrane lipid strips	Ba/F3 cells	ND	ENT, GAIN domain	^114^
G1	Transglutaminase 2		Mouse, Human	Radioimmunoprecipitation assay	Lung, melanoma cells, keratinocytes	ROCK-dependent activation of ADAM17	ENT, STP region	^115^^,^^116^
G1	Collagen III		Mouse	In vitro biotinylation/proteomics and MS	Meningeal Fibroblasts	RhoA activation	ENT, PLL domain	^117^
G1	Laminin				HEK293T	Together with TG2 increase in SRE luciferase		^118^
G1	L-phenylalanine		Human	High resolution mass spectrometry, NMR and coinjection analyses of the active fraction	Supernatant of human gut bacteria	G*α*_s_-G*α*t and G*α*_s_-G*α*_o_ chimera-mediated increase in CRE-SEAP assay		^119^
G1	Heparin		Mouse, Human	ELISA, pulldown	Purified protein	Induction of receptor shedding and increased cell adhesion but no identified increased signaling	ENT, AA 26-35	^120^
G1	Progastrin		Human	Immunofluorescence staining and FACS analysis	Colo320 cells	ND	ND	^121^
G1	CD9, CD81		Human	Mass spectrometry protein sequencing	NT2RA, HEK293 cells	Scaffolding of G*α*11 and G*α*q	ND	^122^
G1	Plectin		Mouse	Unbiased mass-spectrometry screen, co-IP	P5 sciatic nerves	ND	NTF and CTF	^123^
G1		Antibody	Mouse-human chimera	Cell-based fluorescence binding assay	HEK293T	SRF increase	ENT, GAIN domain	^124^
G1		Monobody	Mouse		HEK293T	SRE luciferase inhibition	ENT	^47^
G1		Small molecules incl. dihydromunduletone, 3-*α*-acetoxydihydrodeoxygedunin		In vitro substance screen	Compound library	SRE luciferase activation		125, 126, 127
G2	Dehydroepiandrosterone		Mouse, Human	In vitro substance screen	Compound library	Increase in cAMP	7TMD	^128^
G2	Deoxycorticosteron		Mouse, Human	In vitro substance screen	Compound library	Decrease in cAMP	7TMD	^128^
G3	Cortisol, 11-deoxycortisol			In vitro substance screen	Compound library	Binding to receptor	7TMD	^129^
G3	L-phenylalanine		Human	High resolution mass spectrometry, NMR and coinjection analyses of the active fraction	Supernatant of human gut bacteria	PRESTO Tango Assay activation		^119^
G3		Beclomethasone		In vitro substance screen	Compound library	Go activation	7TMD	^129^^,^^130^
G3		Ezetimibe, Flunarizine, Zeranol		In vitro substance screen	Compound library	ß-arrestin recruitment		^131^
G3		Compound 36, compound 4		In vitro substance screen	Compound library	G*α*_13_ activation	7TMD (assumed)	^127^
G5		Dihydromunduletone, 3-*α*-acetoxydihydrodeoxygedunin	HEK293T cells	In vitro substance screen	Compound library	G*α*_13_ inhibition	7TMD (assumed)	^125^
G6	Collagen IV		Zebrafish	Coimmunoprecipitation	Sciatic nerve	Increase in cAMP	ENT, CUB/Pentraxin	^132^
G6	Collagen VI		Mouse	Pull down coupled to mass spectrometry	Sciatic nerve	G*α*_i_, decrease in cAMP	ENT, GAIN domain	^133^
G6	Laminin-211		Zebrafish, mouse	Coimmunoprecipitation	Sciatic nerve	Increase in cAMP when combined with mechanical forces	ENT, GAIN domain	^132^^,^^134^
G6	Prion protein		Zebrafish	Coimmunoprecipitation	HEK293, SW10 cells	Increase in cAMP	ENT (assumed)	^135^
G6	Progesterone, 17-hydroxyprogesterone		HEK293 cells, breast cancer cells	In vitro substance screen	Compound library	G*α*_i_, decrease in cAMP	7TMD (assumed)	^136^
G6		Apomorphine	Zebrafish, COS7 cells	In vivo substance screen and in vitro verification	Compound library	Increase in cAMP	7TMD (assumed)	^137^
G6		Multiple small molecules	Zebrafish	In vivo substance screen	Compound library	ND		^138^
Subfamily L								
L1	Teneurin-2 (Lasso)		Rat	Affinity purification	Brain lysates	cAMP decrease, Ca^2+^ increase	ENT, RBL/LEC domain	^139^
L1	FLRT1, 3		Rat	Affinity purification	Brain lysates	cAMP increase/decrease	ENT, OLF domain	^140^
L1	Neurexin-1*α*, -1*β*, -2*β*, -3*β*		Rat	Cell binding assays	HEK293 cells	cAMP increase/decrease	ENT, OLF domain	^141^
L1	Contactin-6		Mouse	Affinity purification	Brain lysates and HEK293 cells	Apoptosis	ENT	^142^
L1	Shank		Human, rat	Y2H, Co-IP	cDNA libraries of human brain, brain lysates		ICT	^143^
L1	TRIP8b		ND	Y2H SOS recruitment assay	cDNA library of rat brain		ICT	^144^^,^^145^
L1	glucose		Mouse	Affinity purification-LC/MS, ligand-receptor binding assays	Hypothalamic neurons, CHO cells	G*α*_i_/cAMP decrease	ND	^146^
L1		LK29, LK30, LK31 (engineered synthetic binders)	Human	Single-point protein ELISA, epitope mapping, Surface plasmon resonance			ENT, RBL/LEC domain	^147^
L1		*α*-Latrotoxin		Cell-based Ca2+-uptake and neurotransmitter-release assays	Neurons	Massive neurotransmitter-release	GAIN/TMH1 (residue 467–891)	^148^
L2	FLRT3		Rat	Affinity chromatography, mass spectrometry, coimmunoprecipitation	Brain lysates, transfected heterologous cell lysates	Not determined (ND)	ENT	^140^
L2	Teneurin-2		Mouse	Ligand-receptor binding via SPR	HEK293T		ENT, RBL/LEC domain	^149^
L2	Leucine-rich ɑ-2-glycoprotein 1 (LRG1)		Mouse	Ligand-based receptor capture followed by LC–MS/M	HEK293T	Increase in Lyn-PI3K-AKT-NF-*κ*B p65 pathway		^150^^,^^151^
L3	FLRT1, 3		Rat	Affinity purification	Brain lysates	Not determined (ND)	ENT	^140^
L3	FLRT2		Mouse	Crystal structure, coimmunoprecipotation, surface plasmon resonance	HEK293 cells, HeLa cells, cultured neurons	Cell adhesion and repulsion	ENT, LEC/OLF domain	^20^
L3	UNC5D (only in ternary complex) with FLRT2		Mouse	Crystal structure, pulldown, mass spectrometry	HEK293 cells	Cell adhesion	ENT, LEC/OLF domain	^20^
L3	FLRT3		Human	Crystal structure, cell aggregation assays	HEK293 cells	Cell adhesion	ENT, OLF domain	^19^
L3	Teneurin-3		Rat	Coimmunoprecipotation	HEK293 cells	ND	ENT	^140^
L3		LK29, LK30, LK31 (engineered synthetic binders)	Human	Single-point protein ELISA, epitope mapping, Surface plasmon resonance	-	SRE	ENT, RBL/LEC domain, breaks interaction between L3 with TEN2 but not FLRT3	^147^
L3		LK12 (engineered synthetic binders)	Human	Single-point protein ELISA, epitope mapping, Surface plasmon resonance		SRE	ENT, LEC/OLF domain	^147^
L4	ku80		Human		HUVEC cells	NFAT	ENT	^152^
L4	*β*-spectrin		Human		HUVEC cells	NFAT	ENT	^152^
Cirl	Toll-8/Tollo		*D. melanogaster*	Mass spectrometry, functional readouts	Lysates from *Drosophila* embryo and pupae	ND	ENT	^153^^,^^154^
HC110R	FMRFamide-like neuropeptides AF1, AF10, PF2		*H. contortus*	Surface plasmon resonance	-	ND	ENT	^155^
LAT-1	LAG-2 (DSL protein/Notch ligand)		*C. elegans*	BRET (in vitro) and BiFC (in vivo) assays	Somatic gonad, germ cell	ND	GAIN, RBL domain	^156^
Subfamily V								
V1	Harmonin		Mouse	GST-Pulldown, coimmunoprecipitation, yeast 2-hybrid assays	COS-1 cells, bacterial lysates	ND	ICT, PBM	^157^
V1	Whirlin		Mouse	Glutathione S-transferase (GST) pulldown assays, yeast 2-hybrid assays, immunoprecipitation assays	COS-1 cells, bacterial lysates	-	ICT, PBM	^158^

A recurring feature is a multidomain architecture in which several individually folded domains are attached to 1 another via linkers. Another emerging feature is their ability to form higher-order receptor complexes (Fig. 3A), or signaling hubs, that bring together multiple copies of the receptor and/or multiple ligands in a context-specific way.

ADGRL are exemplary with regard to these features. First described as receptors for the *α*-component of the black widow spider toxin “latrotoxin,”^159^, ^160^, ^161^ they are now known to bind different endogenous ligands such as fibronectin leucine-rich transmembrane protein^140^ and teneurins,^139^ using distinct binding domains (Fig. 3A). Structures suggest the formation of large signaling hubs that involve 3 or more proteins.^17^, ^18^, ^19^, ^20^, ^21^, ^22^ B1 and B3 require glycans—C-mannosylated tryptophan and O-fucosylated threonine residues in their thrombospondin domains—to bind to its ligands, the reticulon-4 receptors (Fig. 3B).^62^^,^^64^ The extended complement C1r/C1s/, Uegf, Bmp1 (CUB) domain^75^^,^^162^ (also referred to as atypical CUB domain^163^) of B3 (Fig. 1A) interacts with its ligand, complement component 1q-like 3 (C1ql3),^76^^,^^164^^,^^165^ Ca^2+^-dependently in a 3:3 stoichiometry.^163^ This hexameric subunit displays an unusual atomic interface architecture that differs from a previously resolved C1q domain-receptor costructure^166^ and may be further joined into higher order complexes, for example, a 36-meric supercomplex.^163^ Crystal structures of E5 in complex with CD55^167^ and of E2^168^ showed that the 3 epidermal growth factor (EGF) domains of E5 have a stable conformation for a *trans*-interaction with 3 short consensus repeat domains of CD55 in an antiparallel orientation (Fig. 3C). However, in many cases, the individual domains of the ENT are connected by flexible linkers. An extreme example is G4, which contains a ∼2000 AA long glycosylated, flexible region between the N-terminal pentraxin domain and the SEA-HormR-GAIN domains (Fig. 3D).^169^

Important insights were gained with the full ENT structures of G6 (Fig. 3E), C1 (Fig. 3F), and G1 (Fig. 3G), which demonstrated that the extracellular domains display varying, isoform-dependent conformations that range from “compact/closed” to “extended/open-like” and dictate conformation-dependent downstream signaling. In some examples, the most distal, N-terminal domain is positioned near the transmembrane region, where it has a regulatory role in signaling.^13^^,^^47^^,^^51^^,^^170^ Contrasting with larger aGPCRs is G3, which contains a minimal GAIN domain and just a short helical extension in the ENT.^48^

Taken together, these results underscore the complexity of aGPCR signaling and the critical roles of their extracellular regions in receptor interaction, function, and regulation.

### C 7TM domains

The N-termini of self-cleaved aGPCR-CTFs are ∼20–25 AA long segments that extend extracellularly from TMH1. In cleaved but nondissociated aGPCR NTF-CTF complexes, these CTF sequences are buried in a *β*-strand configuration within the NTF GAIN domain core (Fig. 4A). After NTF-CTF dissociation, the 7 AA long N-terminus of the CTF is liberated and becomes the TIA activating the CTF and driving G protein signaling, suggesting that the agonists bind intramolecularly to a 7TMD orthosteric site (Fig. 4B).^32^^,^^33^

Cryo-EM structures of TIA-activated G1 and L3, in complex with G*α*_13,_^171^ and D1, F1, G2, G4, and G5, in complex with G*α*_s,_^172^, ^173^, ^174^ revealed a shared hook-like, partial *α*-helical TIA pose that occupied a common orthosteric binding site within the core of the 7TMD bundle (Fig. 4B). The TIA consensus sequence TxFxxLM contacts multiple TMH spans and residues from ECL2.^36^^,^^175^

In TIA-activated aGPCR-G protein complexes, the conserved toggle switch residue W6.53 directly engages the TIA to mediate receptor activation. As demonstrated for E5, W6.53 exhibited a marked rotational shift upon receptor activation, transitioning between inactive and active states.^27^ Unlike class A and canonical class B GPCRs, which depend on conserved activation motifs (eg, PIF, NPxxY, and DRY), aGPCRs lack these canonical sequences. Instead, activated aGPCRs show TMH6-TMH7 breaks or kinks like class B1 GPCR. Notably, the class B1 helix-unwinding residues G6.50/G7.50 are conserved in many aGPCRs, indicating that they are a common feature of TIA-mediated aGPCR activation.

In the glucocorticoid-bound G3 complex, the receptor adopts a 7TMD architecture distinct from that of TIA-activated aGPCRs.^129^ The glucocorticoid interacts with W6.53 to support receptor activation. One unconventional feature of the G3/GPR97-G*α*_o_ complex structure is the palmitoylation of the mini-G*α*_o_ C-terminus, which is inserted deeply into the 7TMD core. This modification is crucial for cortisol-induced coupling of G3 to G*α*_o_. However, it remains unclear whether this modification is present in native G*α*_o_.

Apo structures of E5 and G2 exhibit inactive- and active-state conformations, respectively.^27^^,^^128^ The active-state G2 conformation is indistinguishable for the apo or dehydroepiandrosterone-bound forms, indicating that it is stabilized by the bound G*α*_s_ and nanobody 35, as G2 can be activated by its TIA.^176^ E5 exhibits an inactive, compact 7TMD conformation with pronounced inward shifts of the extracellular ends of TMH6 and TMH7, creating a constrained orthosteric pocket shielded by ECL hydrophobic residues, W685 and F760. The GAIN domain stabilizes an autoinhibited state through direct interactions with ECL1 and 2, whereas the TIA remains sequestered within the hydrophobic core of the GAIN domain, preventing its interaction with the 7TMD core. A conserved "triad tethering motif" (W545-Y683-F760) forms a tight hydrophobic interaction to lock the inactive E5 conformation. Understanding whether these features are common to other apo-aGPCRs requires more structural investigations.

*Critical synopsis and outlook: Structural insights into aGPCRs have fundamentally strengthened earlier concepts of TIA-dependent receptor activation and have enabled the delineation of 7TMD features that distinguish aGPCRs from other GPCR families. Not least, recent structural biology studies revealed the unexpected finding that steroids can bind to and activate aGPCRs by occupying positions that overlap with the canonical TIA-binding site. In the near future, it will be of prime interest to determine whether TIA- and steroid-mediated agonism are mutually exclusive or cooperative modes of receptor activation, how these modes relate to the receptors’ perception of mechanical stimuli, and under which physiological conditions they occur individually or in concert.*

*The GAIN domain will likewise continue to attract structural attention. To date, only a single GAIN–7TMD costructure has been solved, and many more are needed to understand the central role this domain plays in aGPCR signaling behavior. In particular, studies addressing the steric relationship and potential contacts between the GAIN domain and the 7TMD are still lacking. Similarly, the dynamic behavior of the GAIN domain under mechanical stimulation remains ill-defined, and approaches such as* *Förster resonance energy transfer* *imaging, nuclear magnetic resonance, and double electron–electron resonance spectroscopy are in high demand to resolve structural transitions of the domain during receptor activation, signaling, and rest.*

*Structural work has also made major strides in uncovering how individual aGPCRs can engage multiple ligands, which has been a central conundrum in the field for a long time. A complex picture is emerging, in which aGPCRs act as membrane-embedded hubs that integrate diverse extracellular cues—sometimes cooperative, sometimes mutually exclusive, into coherent cellular decisions. Frequently, however, this combinatorial logic remains unresolved. Subsequent work must therefore focus on how the structures of aGPCR-ligand complexes can illuminate cellular responses in the presence of more than 1 ligand for a given receptor. Cryo-electron tomography of intact aGPCR-ligand complexes at endogenous expression sites within their native tissue context remains elusive but will ultimately be required to complement—and in parts correct—our current understanding derived largely from studies of isolated domains or receptor fragments.*

## V Autoproteolytic processing

Autoproteolytic cleavage at the GPS is a defining feature of many aGPCRs.^177^, ^178^, ^179^ Prior to the discovery of the GAIN domain,^25^ a region of approximately 40–60 AAs, which is characterized by 4 conserved Cys, 2 invariant Tyr residues, and 1 consensus tripeptide cleavage sequence, had been dubbed the “GPS motif.”^29^^,^^46^^,^^180^^,^^181^ The GPS proteolytic reaction typically occurs at a 3 AA His(P-2)-Leu/Ile(P-1)-Ser/Thr(P+1) sequence within the GAIN domain (Fig. 2A).^26^^,^^181^ Deglycosylation and pulse-chase experiments suggest that this autoproteolytic process likely takes place in the endoplasmic reticulum (ER) during receptor biosynthesis.^26^^,^^46^^,^^182^, ^183^, ^184^ Subsequent studies have demonstrated that early *N*-glycosylation plays a crucial role in regulating the efficiency of GPS proteolysis, highlighting the stringently controlled nature of receptor cleavage.^184^, ^185^, ^186^ Recent advances have further uncovered key structural determinants and molecular interactions surrounding the GPS, which are essential for ensuring the completion of the autoproteolytic reaction.^52^^,^^187^ Furthermore, translational formation of the 7TMD and distance of the GAIN domain to the inner leaflet of the ER membrane enhances GAIN domain cleavage efficiency, probably by localizing the nascent GAIN domain in proximity to components of the *N*-glycosylation machinery required for correct domain folding and ER exit (see also Section VII).^184^

GAIN domain autoproteolysis dissects the receptor into an NTF and a CTF, which remain tightly bound as a noncovalent complex at the cell surface (Fig. 2A).^28^, ^29^, ^30^ Notably, the NTF of some aGPCRs, such as L3, are, in part, spontaneously shed at the GPS,^188^ whereas the NTFs of other aGPCRs, such as B1 and A2, are cleaved by proteinases at non-GPS locations, releasing different fragments that can mediate non-cell autonomous functions.^189^, ^190^, ^191^, ^192^

Further analysis of crystal, cryo-EM, and AlphaFold structures of various aGPCRs has highlighted the functional significance of the GAIN domain as an evolutionarily conserved protein fold, consisting of subdomains A and B (Fig. 2), necessary and sufficient for receptor autoproteolysis at the GPS.^25^^,^^187^^,^^193^ The first reported structures analyzed GAIN domains of L1 and B3,^25^ which contain a long subdomain A built of 6 *α*-helices. Later studies showed that GAIN domains with a significantly shorter subdomain A, containing only 2–3 *α*-helices, are able to autoproteolyze as well (see sections III and IV.A), for example, G1,^47^ G3,^48^ and E5.^27^ Hence, although the structure of subdomain B appears largely conserved across the aGPCR family, subdomain A exhibits much higher structural variability that is still compatible with GAIN domain autoproteolysis. These structural insights also enhanced the understanding of the molecular basis of *Stachel*-mediated aGPCR activation^171^, ^172^, ^173^, ^174^ and the role of aGPCRs as metabotropic mechanosensitive receptors^89^^,^^94^^,^^153^^,^^194^^,^^195^ (see sections X and XV.D).

Interestingly, several non-aGPCR cell surface proteins, including the sea urchin sperm receptors for egg jelly and polycystic kidney disease 1–like proteins, also contain a GAIN domain and undergo autoproteolysis.^193^^,^^196^, ^197^, ^198^ These findings indicate a broader functional role for GPS cleavage in receptor biology.^14^ As a result, specific point mutations in the GAIN domain that impact receptor structural stability and proteolysis have been associated with a range of unique genetic pathological disorders.^89^^,^^199^, ^200^, ^201^, ^202^ Overall, GAIN domain-mediated autoproteolytic processing is a vital post-translational modification that ensures the correct structural organization and functionalization of many aGPCRs.

*Critical synopsis and outlook: Autoproteolytic processing of aGPCRs has been studied intensively. The remarkable evolutionary conservation of the GAIN domain and its autoproteolytic capacity underscores the substantial contribution this mechanism has made to the fitness of multicellular organisms. Nonetheless, many questions concerning the process itself, its cell biological role, and its physiological consequences remain unresolved. For example, is the steric flexibility of the GAIN domain a prerequisite for, or a consequence of, its self-cleavage? Are there extrinsic factors that cells can deploy to promote or inhibit receptor autoprocessing? Moreover, if the autoproteolytic step serves as a checkpoint for ER exit, what mechanisms does the ER use to assess whether cleavage of a given receptor molecule has taken place? Addressing these questions will be essential to extend our understanding of why many aGPCRs undergo autoproteolysis, whereas others do not.*

## VI Alternative splicing and protein variants of adhesion G protein–coupled receptors

Adhesion GPCRs are encoded by large genes with extended exon-intron structures that facilitate extensive transcript variation through alternative promoter usage and splicing events.^28^^,^^37^^,^^95^^,^^203^, ^204^, ^205^ This genomic complexity enables the generation of multiple mRNA variants from a single gene, which can change the cell expression specificity and/or the open reading frame, potentially resulting in protein variants with distinct structural and functional properties.

**Fig. 5:**
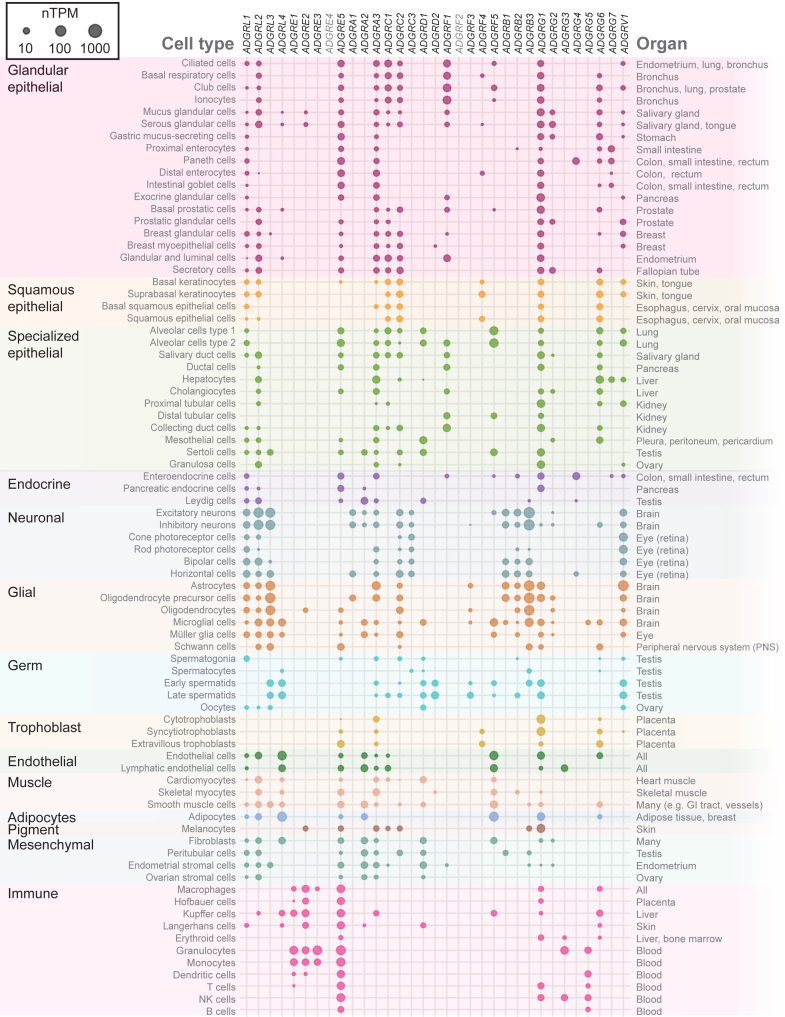
Overview of gene expression of aGPCRs across human cell types obtained by single-cell RNA sequencing data from solid tissues and bulk RNA sequencing data from sorted blood collected by the Human Protein Atlas (HPA) consortium.^206^ Note that (1) cell types are grouped primarily by function, not by tissue, (2) ubiquitous cell types, such as endothelial cells lining vessels, appear only once, although present in many tissues, (3) organs contain various cell types and can appear in multiple categories (eg, liver: hepatocytes and cholangiocytes under “specialized epithelial cells” and Kupffer cells under “immune cells”), (4) hard-to-isolate cells, such as osteocytes and chondrocytes, are not covered in this list, (5) enucleated cells/cell fragments, like red blood cells and platelets, are not included, although they may express aGPCRs, and (6) data represent healthy adult tissues. Normalized transcripts per million (nTPM) values represent the number of transcripts detected for a given gene. The size of the dot depends on the nTPM value (cutoff value of ≥ 4). Each data point represents gene expression and does not distinguish between transcript variants. *ADGRE4*^207^^,^^208^ and *ADGRF2* are currently considered as pseudogenes in humans; however, transcripts originating from both loci have been reported.^21^ See sections VIII and XII.A.

Many aGPCRs and their transcript variants are expressed in a tissue-specific manner (Fig. 5).^206^, ^207^, ^208^ For instance, certain variants are predominantly found in neural tissues, whereas others are more common in immune cells.^47^^,^^204^ This selective expression suggests that different protein forms may be adapted to meet cell-type specific needs. Beyond tissue specificity, spliceosomal reprogramming of aGPCR transcripts was also shown to occur within the same tissue or cell in a stimulus-dependent manner.^209^ A striking example of this comes from the postsynaptic receptor L3, whose ability to promote synapse formation in the hippocampus^210^ relies on 2 convergent pathways G*α*s-mediated signaling and the assembly of postsynaptic scaffold condensates, both of which are governed by activity-dependent alternative splicing of L3.^209^ Furthermore, alternative splicing of the L homolog Cirl in *Drosophila* generates both canonical 7TMD-containing and atypical 7TMD-lacking proteins, whose coexpression enables G protein signaling and the discrimination between different mechanical stimulus intensities in sensory neurons.^211^

Although the identity of the specific factors guiding these processes remains unknown, these data unveil an intrinsic potential for cell autonomous modulation of aGPCR splicing events, which can sustain tissue/cell plasticity throughout development. Comparative analyses across species have revealed that transcript variants are conserved, suggesting that their functional significance may be evolutionarily conserved.^37^^,^^204^

**Fig. 6:**
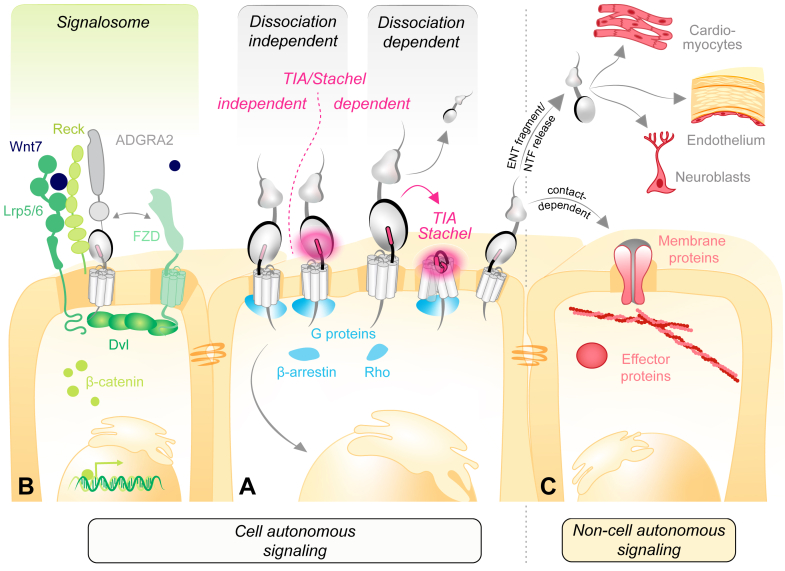
Activation and signaling modes of aGPCRs. (A) Central to the activation of many aGPCRs is the TIA/*Stachel* (in pink), but it is not necessarily a prerequisite for triggering aGPCR activity. Both TIA-dependent and TIA-independent activation modes have been reported. Ligand binding and/or mechanical force application can induce TIA-dependent aGPCR signaling in the intact NTF-CTF receptor complex (dissociation-independent TIA-dependent activation). Alternatively, the bipartite NTF-CTF complex can dissociate and trigger TIA-dependent metabotropic activity (dissociation-dependent TIA-dependent activation). Both scenarios can induce cell autonomous signaling in the aGPCR-expressing cell. (B) Some receptors such as A2 function as part of a signalosome complex, where they not necessarily fulfil a metabotropic function but may be required for other aspects, for example, for ligand recognition. (C) aGPCRs can also relay non–cell autonomous signals via direct membrane-anchored aGPCR-ligand contact between neighboring cells or by shedding ENT fragments, including the NTF that affect the activity of distant cells. See sections X and XI.

Alternative splicing can lead to considerable modifications in receptor structure. Variations may include the differential inclusion or exclusion of extracellular domains, segments of the 7TMD or ICT.^37^^,^^204^^,^^212^ Such differences can influence key aspects of receptor function, including ligand binding affinity, receptor activation, and the modulation of downstream signaling pathways (Fig. 6).^212^, ^213^, ^214^, ^215^ Experimental studies have verified the existence of multiple aGPCR protein isoforms, exhibiting unique signaling properties and protein interaction profiles.^209^^,^^211^, ^212^, ^213^, ^214^, ^215^, ^216^ A well established example of the latter is E5 and its ligand CD55 in the immune system. The E5 ENT exists in 3 different layouts containing between 3 and 5 EGF-like repeats, each of which is characterized by a different binding affinity to CD55 (Fig. 3C).^28^^,^^203^^,^^217^ Furthermore, a prominent protein variant of L3 that excludes an element with inhibitory influence on transsynaptic interactions diminished Ca_V_1.4 calcium channel activity, profoundly disrupted synaptic release by cone photoreceptor cells, and resulted in synaptic transmission deficits.^218^ The L ortholog in *Drosophila*, Cirl, is alternatively spliced, giving rise to structurally disparate protein variants that act in concert to control mechanosensitive bandwidth in peripheral neurons.^211^ In *Caenorhabditis elegans*, variants comprising only the ENT of L/LAT-1 and therefore lacking a functional 7TMD are present and could mediate 7TMD-independent function of the protein in the reproductive system in a non–cell autonomous manner (Fig. 6C).^219^ G1 has multiple variants, and experiments with transgenic mice showed that the S4 variant is dispensable for cortical development and central nervous system (CNS) myelination but is essential for microglia-mediated synaptic pruning.^220^ Use of alternative promoters is another way to generate protein diversity and differential regulation. For example, the *B1* gene has an alternative promoter in the distal portion of intron 17 that drives the synthesis of shorter B1 isoforms lacking the ENT and resembling the ICT obtained after autoproteolytic cleavage at the GPS but with variable N-termini that can include new AAs.^205^ These findings highlight the functional heterogeneity within the aGPCR class and illustrate how structural differences arising from alternative splicing can determine specific cellular outcomes.

The impact of natural, disease-causing mutations further emphasizes the importance of alternative splicing. Mutations located in exons common to all splice variants generally lead to a broad loss of receptor function, whereas mutations confined to alternatively spliced regions may selectively impair individual isoforms.^37^ One such example is provided in the C-terminus of C1, which varies in length and sequence. Here, a P^2983^A mutation leads to faulty protein trafficking and is associated with neural tube defects.^221^ However, P^2983^A is located in the variable C-terminus of C1 and warrants thorough investigation of its pathogenicity. Such a differential effect may account for tissue-specific disease manifestations and underscores the necessity of considering the splicing landscape when investigating the molecular basis of aGPCR-related disorders. The presence of multiple splice variants also carries significant experimental implications. When generating transgenic animal models or developing antibodies, it is crucial to consider the full spectrum of aGPCR variants to avoid misinterpretation of experimental results. Furthermore, specifying the exact transcript variant used in functional or structural analyses is essential for ensuring reproducibility and for accurately correlating observed phenotypes with specific protein isoforms.

In summary, alternative promoter usage and alternative splicing are fundamental mechanisms that contribute to the structural and functional diversity of aGPCRs, thereby influencing receptor activity in both physiological regulation and pathology.

*Critical synopsis and outlook: Motivated by the complex genomic architecture of many aGPCR loci, analyses of their splicing repertoires have begun to reveal a fascinating versatility in their gene products. This diversity ranges from subtle structural variations—some with critical consequences for receptor signaling—to major alterations that generate products no longer conforming to a canonical GPCR architecture. Thus far, only a few studies have traced individual splice forms through to their protein products and examined their occurrence and physiological functions in animal models; however, these have already uncovered intriguing roles in aGPCR activity. Many more such in-depth structure-function analyses will be required to understand how a cell chooses to express a particular splice form or a subset of splice forms of an aGPCR gene, and to what end.*

## VII Subcellular trafficking, localization, and cellular functions of adhesion G protein–coupled receptors

Adhesion GPCRs mediate cell-cell or cell-matrix interactions, thus requiring proper trafficking to the plasma membrane.^114^^,^^210^^,^^222^^,^^223^ Anterograde trafficking guiding aGPCRs to the plasma membrane follows the ER-Golgi route, with the ENT acquiring *N*-glycosylation and mucin-type O-glycans.^224^, ^225^, ^226^, ^227^ Experiments with L1, E2, and D1 have shown that the GAIN domain undergoes autoproteolysis in the ER.^26^^,^^28^^,^^29^^,^^46^^,^^184^^,^^224^^,^^226^

Our understanding of molecular cues directing aGPCR intracellular trafficking has largely been provided by mutational analyses. Mutagenesis of G1 with disease-relevant point mutations, including in the GAIN domain, unveiled disruption of its anterograde transport and accumulation in the ER.^200^^,^^201^^,^^228^^,^^229^ However, mutations affecting self-cleavage within or in the immediate vicinity of the GPS in E2, E5, and D1 did not alter the receptors’ principal ability to be trafficked to the plasma membrane but rather the extent of protein exiting the ER.^30^^,^^184^^,^^226^^,^^230^ In addition, also uncleaved subpopulations of cleavage-competent receptors were observed to be trapped in the ER, potentially due to their incomplete or improper GAIN domain folding.^184^^,^^226^ These observations suggest that correct protein folding, but not GPS cleavage, is necessary for anterograde trafficking of aGPCR to the plasma membrane. GPS cleavage may act as a folding indicator before ER exit, but is not absolutely required for it. However, the importance of autoproteolytic cleavage for these phenomena may vary among aGPCRs.^25^^,^^46^^,^^184^^,^^231^

*N*-glycosylation is an important determinant of aGPCR self-cleavage, maturation, and trafficking.^184^, ^185^, ^186^ Mutating *N*-glycosylation sites in the E2 NTF produced variable effects on plasma membrane localization of the receptor,^227^ whereas pharmacologic inhibition of glycosylation or glycosylation-incompetent mutants impaired autoproteolysis of E2^184^ or E5.^185^ Furthermore, *N*-glycosylation within the G6 NTF was shown to affect ER exit, whereas GPS cleavage was dispensable for membrane trafficking.^186^

Retrograde trafficking of aGPCRs from the plasma membrane can occur spontaneously or upon ligand binding, as exemplified by L1, whose NTF and CTF undergo internalization into distinct endocytic organelles,^141^^,^^224^ some identified as early endosomes.^232^ Studies with G2 suggest that arrestin-dependent endocytosis of its CTF is prominent upon dissociation from the NTF.^233^ In contrast, A3 undergoes endocytosis via an arrestin-independent, clathrin-mediated mechanism.^234^

V1, the largest aGPCR, is an interesting example of the wide variety of cellular functions mediated by a single receptor. V1 is found in the microvilli-like stereocilia of the mechanosensory hair cells in the inner ear and at the cilium of the photoreceptor cells of the retina and forms adhesion complexes with other molecules associated with Usher syndrome.^235^ These depend on the formation of fibrous links between neighboring membranes by its exceptionally long ENT. V1 is targeted to the base of primary cilia by its interaction with a cytoplasmic chaperonin complex consisting of T-complex protein 1 ring complex/chaperonin-containing T-complex protein 1 chaperonins and the Bardet-Biedl syndrome chaperonin-like proteins.^236^^,^^237^ V1 was also described as a metabotropic mechanoreceptor in focal adhesions controlling cell shape and motility.^238^^,^^239^ Furthermore, V1 is a component of specialized mitochondria-associated ER membranes, where it is involved in Ca^2+^ release from the ER and its uptake into mitochondria, thus regulating cellular Ca^2+^ homeostasis.^240^ Finally, V1 can inhibit the autophagy process at different steps.^241^

*Critical synopsis and outlook: In recent years, several connections have emerged between the post-translational processing of aGPCRs and their intracellular localization, helping us to bridge gaps in our understanding of aGPCR self-cleavage, glycosylation, and subcellular trafficking. For a few receptors, a basic understanding of their distribution within specific cell types has begun to take shape. However, it remains striking that for many aGPCRs, their proposed roles in sensing adhesive or mechanical cues are not yet supported by direct evidence of their localization to the subcellular sites where such cues are encountered. This includes a broader lack of studies addressing the colocalization of aGPCRs with their cognate ligands. A coordinated effort will be needed to address this substantial deficit in our understanding of aGPCR biology. Emerging technologies—such as advanced super-resolution microscopy, next-generation protein-labeling approaches including genetic code expansion and click chemistry, and the continuous development of highly specific binders (antibodies, nanobodies, and monobodies)—promise to accelerate this progress. Together, these tools will be essential for determining the precise localization of aGPCRs at their native expression sites within cells.*

## VIII Anatomical and cellular distribution of human adhesion G protein-coupled receptor transcripts

**Fig. 7:**
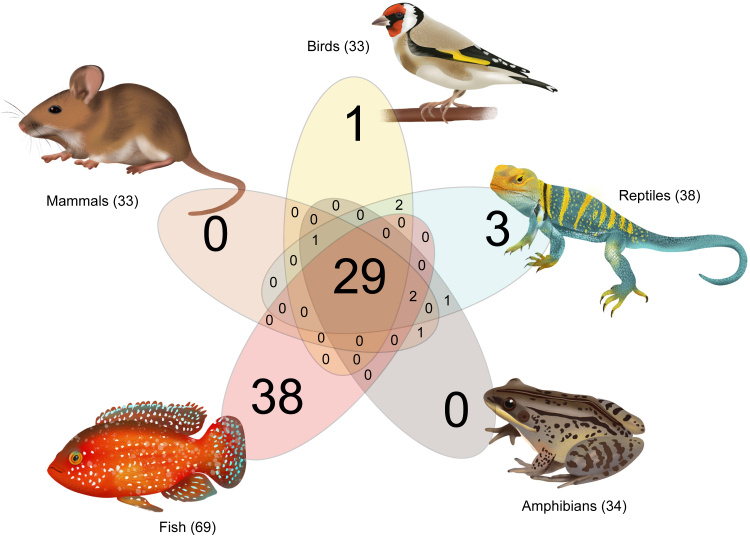
The number of shared and private aGPCRs in vertebrate genomes. The dataset of human aGPCR genes (including the pseudogene *E4*) was used to extract the orthologous aGPCRs from all vertebrate classes using the webtool OrthoDB v11.^243^ In total, 87.9% of human aGPCRs are shared within vertebrates as shown in the Venn diagram. The high number of fish aGPCR is mainly due to genome duplication in some ray-finned fish (*Actinopterygii*). Of note, the number of private fish, amphibian, reptile, and bird aGPCRs is biased by the fact that only human aGPCRs were used to extract the orthologous sequences. Animal icons are examples. See section III.A.

Mammalian genomes contain a collective of 33 aGPCR loci, but the count in individual species varies (Fig. 7).^1^^,^^15^^,^^242^^,^^243^ For example, as the mouse genome encodes 31 aGPCRs, as orthologs of *E2* and *E3* are lacking,^244^, ^245^, ^246^ and because *D2* is considered a pseudogene,^15^ only 30 aGPCR genes are likely to produce functional receptor proteins in the mouse. Of the 33 human aGPCR loci, *E4* and *F2* are currently annotated as pseudogenes. Although some studies reported that these loci generate transcripts that encode truncated proteins^207^ or are transcriptionally inactive (Fig. 5), respectively, other studies detected the expression of *E4* and *F2* transcripts.^37^ Until it is conclusively demonstrated that these transcripts fail to produce functional proteins and that the loss of *E4* or *F2* has no phenotypic consequences, their gene status remains unresolved.

Figure 5 summarizes single-cell RNA sequencing data across 31 human tissues and bulk RNA sequencing of blood samples for all aGPCRs. It is unlikely that any of the approximately 200 cell types in the human body completely lack aGPCR expression. In Fig. 5, the complex data are visually simplified by grouping cells with similar or overlapping functions, even if they originate from different organs. For example, serous secretory epithelial cells are found in the exocrine pancreas, as well as in salivary and mammary glands. These cells may not share the same embryonic origin. Interestingly, the presence of an aGPCR in a specific cell layer of 1 organ often corresponds to its presence in the same cell layer of another organ. For instance, *F4* is found in the epidermis, the squamous (cornified) epithelium of the skin,^247^ and is likely also present in organs or cavities lined by squamous (noncornified) epithelium.

Some aGPCR transcripts are omnipresent in various functionally distinct cell types. The most prevalent examples are *E5* and *G1*. *E5* is not only predominantly expressed in immune cells but also at moderate levels in smooth muscle,^248^ skeletal muscle,^249^ and lung epithelial cells. The presence of *G1*, first shown in circulating natural killer (NK)^250^ and cytotoxic T cells,^251^ has been confirmed for many (secreting) epithelial cells, as well as microglia and astrocytes.^252^

On the other hand, there are more selective aGPCRs expressed in only a few cell types. This applies to *E1–E3*, *G3*, and *G5*, all expressed by immune cells.^253^ Furthermore, *D2* and *F3* are restricted to spermatids and spermatids/glial cells, respectively, whereas *G4* is a specific marker of intestinal enteroendocrine/enterochromaffin and Paneth cells. No transcripts encoding *F2*, which shares notable sequence homology with *F4*, were found in any tissue in the current analysis.

Although the cellular expression patterns of aGPCRs are becoming increasingly elucidated, there are cell types, such as osteocytes in bone and chondrocytes in cartilage, that are difficult to isolate for RNA sequencing, leaving knowledge lagging behind.^254^ Moreover, most of these transcriptomic data still require validation at the protein level. One reason for the gaps in our knowledge about protein expression is the lack of thoroughly evaluated antibodies for most aGPCRs. Exceptions are antibodies targeting members of the E, G, and L subfamilies. Generating a comprehensive set of tools for detecting aGPCRs at the protein level would benefit from a community effort, which includes rigorous quality controls such as cells expressing ectopically tagged aGPCRs and CRISPR-generated aGPCR-deficient cell lines.^252^^,^^255^ Another reason is the structural complexity of aGPCRs due to extensive alternative transcription,^37^ splicing,^204^ and posttranslational modification. These processes result in cell type- and context-specific expression of isoforms (eg, E2 and E5),^256^ protein truncation (eg, by proteolytic cleavage within or outside the GPS),^178^ and glycosylation (eg, E5).^248^^,^^257^

*Critical synopsis and outlook: Modern single-cell sequencing technologies have enormously widened the grasp of cellular expression patterns for aGPCRs. However, as with the demanding transition from splice- to isoform-level repertoires, single-cell protein expression studies that extend transcriptional information to the translational landscape are still lagging behind. Such analyses are urgently needed to establish where—and ideally at isoform-specific resolution—individual aGPCRs execute their functions.*

## IX Interactions

### A Ligands (extracellular binding partners)

As bona fide adhesion molecules, aGPCRs participate in both heterophilic and homophilic protein-protein interactions (PPIs) that stabilize cell-cell or cell-matrix contacts while also allosterically regulating receptor signaling. Interactions with extracellular ligands are mediated by subfamily-specific aGPCR domains and the GAIN domain located in the ENT (Table 1, Fig. 3). Efforts to identify extracellular ligands have been carried out in the cellular and tissue context in which individual aGPCRs are expressed. These efforts aim to shed light on a plethora of physiological processes involving a wide range of receptor-ligand pairs. Early work on the immunological response led to E5 being among the first deorphanized aGPCR.^96^ Meanwhile, the neurotropism of an exogenous toxin would lead to its pairing with L1.^29^^,^^258^ Subsequent studies on tissue development revealed that, thus far, only members of subfamilies C^79^^,^^259^ and V^260^^,^^261^ engage homophilically as adhesive aGPCR pairs. These observations highlight the potential of aGPCRs to participate in diverse types of ligand interactions.^98^^,^^139^^,^^262^

The molecular basis for this ligand diversity resides in the presence of distinct domains, many with adhesive functions, organized into separate modules in different aGPCR subfamilies (Figs. 1 and 3). For example, G subfamily members interact with cell surface antigens, phospholipids, extracellular matrix (ECM) molecules, steroid hormones, or small molecule ligands through structurally distinct N-terminal adhesion modules (Fig. 3D, E, G) or within their transmembrane domains.^114^^,^^115^^,^^117^, ^118^, ^119^, ^120^^,^^122^^,^^125^^,^^128^^,^^138^^,^^263^ Interaction patterns are prone to further diversification as adhesion modules can accommodate many ligands at once,^210^ be modified by alternative splicing to regulate ligand binding,^114^^,^^214^ or stabilize interactions occurring in *trans* (across cells) (Fig. 3A–D) as well as in *cis* (within the same cell) configurations (Fig. 3).^141^^,^^142^^,^^264^

Transcending their mere adhesion role, a growing number of aGPCRs is shown to utilize their ligand interactions to initiate signaling through both G protein–coupled mechanisms^86^^,^^94^^,^^108^^,^^213^^,^^265^ and G protein–independent pathways.^53^ These advances have facilitated the identification of ligands with both positive and negative allosteric effects on aGPCR signaling.^108^^,^^111^

Emerging from these studies is a pattern of wide physiological relevance, wherein a given aGPCR can engage with distinct cellular contexts to regulate cell-specific functions (Fig. 5). For example, F5 binding with its ligand sFNDC4 in adipose tissue contributes to glucose uptake,^113^ whereas in the lung, its interaction with the Sp-D ligand regulates surfactant homeostasis.^112^^,^^266^^,^^267^ However, more than 30% of vertebrate aGPCRs still lack assigned ligands including members of the V subfamily, whose ligand-pairing remains elusive despite or because it harbors the largest ENT among all aGPCRs.^238^ Future efforts focusing on ligand discovery are likely to enhance our understanding of aGPCR functions.

### B Intracellular binding partners (except G proteins, arrestins, and Rac/Ras/Rho GTPases)

Many aGPCRs bind to postsynaptic density protein 95/disc large tumor suppressor/zona occludens 1 (PDZ) scaffold proteins via their cytoplasmic C-termini. Notably, nearly half of the 33 mammalian aGPCRs possess a PBM—typically the last C-terminal 4–5 AAs of the C-terminus—that can specifically dock into the binding pocket of target PDZ domains.^268^ These PPIs can influence receptor signaling, link receptors to the cytoskeleton, and/or influence the subcellular localization of both the receptors and their PDZ partners (Fig. 6A). For example, mechanical force-induced phosphorylation of E5 in its PBM alters the intracellular binding of E5 to the F-actin cytoskeleton.^107^

Several aGPCR interactions with PDZ proteins have been reported. For example, L and B receptors are targeted to the postsynaptic membrane of neurons by binding to PDZ domains of the Shank (SH3 and multiple ankyrin repeat domains) and membrane-associated guanylate kinase proteins.^143^^,^^269^^,^^270^ In sensory cells, V1 is integrated into adhesion complexes by binding to the PDZ proteins harmonin, whirlin, and PDZD7, as well as to other proteins related to the Usher syndrome.^157^^,^^158^^,^^236^^,^^268^ In non-neuronal cells, the membrane-associated guanylate kinase protein DLG1 interacts with A2, A3, and E5.^63^^,^^107^^,^^271^ A2, A3, and C1 interact with the PDZ protein Dishevelled to modulate WNT signaling (Fig. 6B).^54^^,^^272^^,^^273^ A2 was also found to interact with MAGI3 and DLG4237.

Regarding non–PDZ-mediated PPIs, E5 binds to *β*-catenin independently of the WNT pathway.^274^ B1, B3, and A2 interact with the ELMO/DOCK180 complex regulating the F-actin cytoskeleton via RAC.^67^^,^^275^, ^276^, ^277^, ^278^ Additionally, the tetraspanin CD81 binds to G1, inhibiting NK cell activation.^279^

Systematic affinity proteomic screens have validated known PPIs and have identified numerous novel putative interacting partners for A1-3, B1-3, D1, E5, L2, and V1.^24^^,^^262^^,^^280^ These PPIs were associated with synapses, focal adhesions,^238^ mitochondria, the ER, ER-plasma membrane bridges,^280^ mitochondria-associated ER membranes,^240^ autophagosomes,^241^ the *γ*-secretase complex, nuclear-cytoplasmic shuttling, and primary cilia. At primary cilia, V1 was found to bind to CCT molecules of the TRiC-chaperonin complex and chaperonin-like Bardet-Biedl syndrome proteins.^236^^,^^237^ V1 also binds to the Sigma-1 receptor and ACSL4 in mitochondria-associated ER membranes and controls Ca^2+^ flux from the ER,^240^ whereas suppression of D1 signaling by ESYT1, a Ca^2+^-dependent ER-plasma membrane molecular bridge, is abrogated by Ca^2+^ elevation.^280^

*Critical synopsis and outlook: Recent work has uncovered a strikingly diverse interaction landscape for aGPCRs, spanning extracellular ligands and intracellular binding partners. Extracellularly, aGPCRs engage heterophilic and homophilic partners through subfamily-specific adhesion modules and the GAIN domain, enabling both adhesive functions and allosteric regulation of signaling. The breadth of identified ligands—from ECM proteins and cell-surface antigens to steroid hormones and small molecules—highlights the modularity and contextual adaptability of their ENT architecture. However, more than one-third of vertebrate aGPCRs remain orphan receptors, illustrating persistent gaps in ligand discovery and tissue-specific mapping. Intracellularly, many aGPCRs harbor PBMs that link them to synaptic scaffolds, cytoskeletal networks, and polarity complexes, whereas additional non–PDZ interactions connect them to pathways controlling actin remodeling, organellar crosstalk, autophagy, and Ca^2+^ flux. Proteomic screens have further expanded this repertoire, revealing associations with mitochondria, ER-plasma membrane bridges, focal adhesions, and primary cilia. Looking ahead, a central challenge will be to mechanistically integrate these diverse interactions into unified models of aGPCR function. Key priorities include systematic ligand discovery, structural elucidation of receptor-partner complexes, and resolving how context-dependent extracellular and intracellular interactions converge to shape signaling bias. Such efforts will be essential for unlocking the pharmacological potential of this receptor family.*

## X Receptor activation modes

### A TIA/Stachel-dependent signaling: Dissociation model

Autoproteolytic cleavage of aGPCRs, first observed for E5^28^ and L1,^29^ results in the presentation of stable NTF-CTF receptor complexes at the cell surface and is essential for the subsequent dissociation capacity of receptor fragments (Fig. 6).^26^^,^^30^ Self-cleavage is essential for the function of many aGPCRs. This is exemplified, for example, by a mouse strain carrying a cleavage-deficient F5 point mutant knock-in as it displayed the same phenotype of pulmonary surfactant oversecretion as *F5* knockout (KO) animals.^281^ Engineered NTF deletions of G1 and B2 (ie, CTF-only) had elevated signaling.^73^^,^^282^^,^^283^ Numerous studies reporting enhanced constitutive activity of expressed CTF-only receptors support these findings.^32^^,^^33^^,^^73^^,^^282^, ^283^, ^284^ The observation of receptor NTF-CTF complex dissociation via NTF release sensors (NRSs) of endogenously expressed aGPCRs in *Drosophila* demonstrated NTF-CTF separation in vivo.^153^ Recovery of isolated aGPCR NTF from tissues and cells further corroborated NTF-CTF complex dissociation models of aGPCRs.^285^^,^^286^ Additionally, chaotropic salt was used to dissociate NTFs from CTFs in overexpressed aGPCR membrane preparations, leading to robust receptor and G protein activation.^33^^,^^287^

These observations, along with structural insights revealing the autoproteolytic mechanism of the GAIN domain,^25^ have led multiple groups to propose a model for the enhanced activity of isolated aGPCR-CTFs. In this model, the residual ENT of the CTF is masked by NTF structural elements in intact NTF-CTF complexes; upon fragment dissociation, the CTF N-terminus is decrypted and binds intramolecularly to an orthosteric site within the CTF, serving as a TIA (Figs. 4B and 6A). A series of complementary studies with G1, G2, G5, G6, D1, F1, and L3 support this TIA hypothesis.^33^^,^^176^^,^^216^^,^^287^, ^288^, ^289^ Furthermore, multiple structural analyses have demonstrated the commonality of TIA binding to aGPCR/G protein complexes^36^^,^^175^ and are described in detail in section IV.

Acute activation of aGPCRs by exogenous proteases at engineered cleavage sites positioned N-terminal to the TIA of L3^290^ and D1^226^ helped confirm the model. A follow-up study found that TIA-mediated signaling of L3 required NTF dissociation and that ∼5% of the receptor population spontaneously sheds its NTF, potentially accounting for the majority of basal activity (Fig. 6A).^188^ This parallels a study in which D1 was activated by an NTF-specific antibody in a cleavage-dependent manner; presumably, the antibody promoted partial NTF dissociation.^291^ Modes of TIA-dependent and independent aGPCR activation mechanisms have been reviewed extensively (Fig. 6A).^292^^,^^293^ A recent profiling study of TIA-dependent signaling and trafficking of human aGPCRs supported the TIA activation model for most receptors, including those above.^284^ However, despite strong TIA sequence conservation across the entire receptor panel, not all aGPCRs exhibited TIA-dependent signaling (Fig. 6A), which correlated with AlphaFold predictions identifying receptors unlikely to exhibit TIA-binding to the CTF.^284^

### B TIA/Stachel-dependent signaling: Nondissociation model

In addition to the dissociation-dependent activation of aGPCRs, findings conflicting with this activation scenario have also been reported. Several in vitro and in vivo studies have indicated that receptor self-cleavage is not essential for signaling or cell autonomous functions of certain aGPCRs supporting a model in which NTF release is not required to induce aGPCR activation via the TIA (Fig. 6A). However, not all reports dissected basal receptor activities from TIA-mediated stimulation.

For example, a study in *C. elegans* indicated that a cleavage-deficient mutant of LAT-1/L can rescue phenotypic defects similar to the cleavable receptor.^294^ However, its metabotropic signaling activity was not examined at the time, and since the TIA sequence had not yet been described, its necessity for receptor function was not assessed. Its metabotropic signaling activity was discovered thereafter, and its TIA sequence identified.^295^ Similarly, basal activities of cleavage-deficient mutants of D1,^296^ G1,^297^ and G2^298^ were not significantly affected in all examined pathways. However, a contribution of the TIA to these signals was not directly tested. Of note, when dissecting the contribution of self-cleavage from agonism, it is important to consider that the most C-terminal AA of the HL/T cleavage triad overlaps with the N-terminus of the TIA. A study in *Drosophila melanogaster* actively addressed this by creating H>A (−2) and T>A (+1) alleles of the L homolog Cirl, showing that disruption of the internal agonist (T>A) failed to rescue the receptor function, whereas the lack of cleavage showed wildtype results.^195^ Additional support for cleavage-independent and thus nondissociative aGPCR activation via the TIA sequence comes from uncleavable G5 and D1 receptors, which can be activated by mechanical forces in an TIA-dependent manner.^172^^,^^216^ However, conflicting reports exist for both examples,^287^^,^^291^ and studies frequently differ in their observations regarding self-cleavability of aGPCRs,^216^^,^^291^^,^^299^

### C TIA/Stachel-independent signaling

Although the TIA is clearly important for the activation of signaling by several aGPCRs, it is not universally important (Fig. 6A). For example, deletion or mutation of the TIA has little impact on G protein–mediated signaling by B1,^297^ C1, or C3,^300^ whereas parallel studies revealed signaling by G1^297^ and C2^300^ to be heavily TIA-dependent. Similarly, mutation of the TIA was found to have no effect on stimulation of full-length G1 by an activating antibody,^301^ although TIA exposure is essential for signaling by the isolated G1 CTF region.^33^^,^^297^ These observations demonstrate that the importance of the TIA for signaling varies by receptor as well as by the mode of activation.

Consistent with these findings, a recent comprehensive analysis of G protein–mediated signaling by the CTF regions of all human aGPCRs revealed TIA-dependent signaling in approximately half the receptors.^284^ In contrast, the signaling activities of the other aGPCRs examined in this study appeared to be TIA-independent. Intriguingly, AlphaFold models of the isolated CTF correlated strongly with the TIA dependence of signaling for each receptor, with intramolecularly bound TIA sequences predictive of TIA-dependent signaling.^284^

Two distinct views have emerged regarding TIA-independent aGPCR signaling. First, for some aGPCRs, there may be additional points of contact between the NTF and CTF beyond those by which the NTF sequesters the TIA. These interactions may enable the NTF to influence CTF conformation, consistent with recent cryo-EM studies on L3 showing that the GAIN domain exhibits conformational coupling with the 7TMD region.^50^ Second, TIA-independent signaling could be indicative of tonic signaling capabilities by certain 7TMD, which could feasibly underlie long-lasting actions of particular aGPCRs, such as control of planar cell polarity (PCP) by C1-3^302^^,^^303^ or conventional dendritic cell type 2 positioning in the spleen by E5.^94^ In this model, an important role of the NTF may be to guide the subcellular targeting of the CTF to ensure that TIA-independent signaling occurs in the right location.

*Critical synopsis and outlook: How aGPCRs become activated has naturally been one of the most intensely studied questions in the field. The demonstration of tethered agonism more than a decade ago catalyzed research on individual aGPCR functions across pharmacological, structural, and physiological levels. Together with the discoveries of receptor dissociation through autoproteolytic processing and the mechanosensitivity of many aGPCRs, the role of the TIA in receptor signaling has remained a central focus of investigation. Despite substantial progress—supported by structural studies elucidating the conformation of the TIA bound to the 7TMD—several observations remain incompatible with the most parsimonious model of TIA-dependent activation via receptor dissociation. Chief among these is the finding that nonautoproteolyzed aGPCRs can nevertheless signal in a TIA-dependent manner. In addition, convincingly, TIA-independent signaling events have been documented, raising the question of whether these distinct signaling modes can be mediated by the same receptor protein and under which conditions aGPCRs may become biased toward one mode or another. A critical and systematic dissection of these phenomena is therefore warranted to reconcile these disparate mechanistic models and define the principles that govern aGPCR activation.*

## XI Signaling routes

### A Cell-autonomous signaling

#### 1 G proteins

G proteins are the primary transducers of most GPCR-driven cellular responses (Fig. 6A). Receptors bind G proteins and catalyze nucleotide exchange, enabling interaction with various secondary effectors to propagate intracellular responses.^304^^,^^305^

Evidence linking aGPCRs with G protein–mediated signaling was derived from affinity chromatography and immunoprecipitation studies, which revealed interactions between G proteins and L1^161^ and G1.^122^ Subsequently, regulation of Ca^2+^ mobilization and RhoA activation, indicative of G protein signaling, were observed downstream of L1^44^ and G1,^306^ respectively. Mice lacking G1 or collagen III exhibit similar cerebral cortex defects as animals with neuronal G*α*12/13 KOs,^117^^,^^307^, ^308^, ^309^ suggesting a link between collagen binding to G1 and G12/13 signaling. A diversity of G protein-coupling partners has since been indicated for multiple aGPCRs.^32^^,^^33^^,^^107^^,^^130^^,^^176^^,^^209^^,^^211^^,^^216^^,^^233^^,^^270^^,^^287^^,^^290^^,^^296^^,^^310^, ^311^, ^312^, ^313^, ^314^

Biochemical studies showed that the TIA of aGPCR mediates receptor activation and stimulation of G protein GTP*γ*S binding.^32^^,^^33^ This approach helped identify many other aGPCR-G protein coupling interactions.^171^^,^^287^^,^^290^

A large-scale profiling study used G protein conformational biosensors and signaling assays downstream of G protein activation to define the TIA-dependent coupling patterns of the human aGPCR family in a single system.^284^ This identified a preference for signaling through the G*α*_12/13_ subfamily, which activates RhoGEF to control cytoskeletal dynamics, thereby influencing cell migration, contractility, shape, and adhesion.^315^, ^316^, ^317^ All these biological activities align well with the role of aGPCRs as mechanoreceptors.

The concordance of receptor and G protein KO phenotypes,^94^^,^^295^ the stimulation of aGPCR signaling by synthetic peptide agonists,^286^^,^^318^ and the disruption of secondary messenger pathways upon receptor deletion or mutation^134^^,^^209^^,^^211^^,^^295^^,^^319^ have collectively confirmed several aGPCR-G protein signaling axes in vivo. Specific G protein signaling pathways have also been linked to various aGPCR-driven human disease states.^320^, ^321^, ^322^ However, the physiological significance of other biochemically identified aGPCR-G PPIs remains to be investigated.

#### 2 Arrestins

Arrestins are cytosolic proteins that facilitate the desensitization and internalization of activated and phosphorylated GPCRs. Receptor phosphorylation by GRKs (G protein receptor kinases) (Fig. 6A) creates a phosphorylation pattern of serine or threonine residues, which directs arrestin interactions and function. In addition to turning off G protein–mediated signaling, recruitment of arrestins to GPCRs can result in the initiation of signaling cascades by scaffolding and activating effector proteins like ERK1/2 or JNK3.^323^^,^^324^

Regarding arrestin interactions with aGPCRs, the CTF of G1,^283^^,^^297^ B1,^270^^,^^297^ B2,^320^ and G2^233^ have been shown to robustly coimmunoprecipitate with arrestins. In the case of G2, arrestin binding can facilitate complex formation with cystic fibrosis transmembrane conductance regulator (CFTR).^314^ In bioluminescence energy transfer (BRET)-based studies, both F5^318^ and L3^290^ have shown to recruit arrestins in an activity-dependent manner.

Recently, comprehensive BRET assays were performed with all human aGPCR CTF constructs to determine potential arrestin interactions.^284^ In these studies, TIA/*Stachel*-dependent recruitment of arrestins was observed for E1, E3, F1, F4, and G7. Similarly, recent screening using a cumate-inducible Tango-Trio assay revealed arrestin binding to A1, A3, F1, G1, G2, and G5.^325^ Further work in this area is likely to reveal many more examples of aGPCR regulation and signaling mediated by arrestin recruitment.

#### 3 Signaling pathways independent of G proteins and arrestins

Beyond their best-characterized G protein and arrestin pathways, aGPCRs signal through noncanonical mechanisms that utilize intracellular scaffolds and ectodomain-assembled receptor complexes (Fig. 6A). For example, subfamily B members control Rho-GTPase signaling and cytoskeletal remodeling by interacting with Rho-GTPase regulatory proteins (GEFs/activators and GAPs/inhibitors) via their large ICTs. B1 and B3 are associated with the ELMO/Dock180 Rac1-GEF complex to drive Rac1-dependent phagocytosis, myoblast fusion, and neuronal morphogenesis,^67^^,^^69^^,^^277^^,^^278^^,^^326^ although the expression of B1 in macrophages remains debated.^255^ In neurons, B1 recruits the Par3/Tiam1 Rac1-GEF complex to synapses to promote Rac1-dependent dendritic spine and excitatory synapse development,^66^^,^^222^ and later engages the Rac1-GAP/RhoA-GEF Bcr to switch signaling toward RhoA-dependent dendrite growth arrest.^327^ Additionally, B1 ICL1 interacts with the E3 ubiquitin ligase MDM2, preventing MDM2-mediated PSD95 and p53 degradation and impacting synaptic plasticity and medulloblastoma tumorigenesis.^328^^,^^329^ These findings highlight ICT-dependent effectors as aGPCR signal transducers beyond G proteins and arrestins.

#### 4 Signaling with coreceptors

Adhesion GPCRs interact with various coreceptors to regulate intrinsic G protein signaling or extrinsic signaling through associated receptors (Fig. 6B). These interactions are crucial for diverse physiological processes, including vascular development, neural functions, and immune responses.

E5 was the first aGPCR shown to heterodimerize with another receptor, the lysophosphatidic acid receptor 1, leading to amplified LPA-dependent Rho and extracellular signal-regulated kinase activation.^102^^,^^103^

A2 forms complexes with the WNT coreceptors RECK, FZD, and LRP5/6, regulating WNT7A/B-specific *β*-catenin signaling in brain endothelial cells, crucial for angiogenesis in the developing CNS and for blood-brain barrier integrity (Fig. 6B).^53^, ^54^, ^55^, ^56^, ^57^^,^^330^^,^^331^

B1 and B3 interact with RTN4/NoGo receptors regulating neuronal development.^62^^,^^64^

The cadherin-like C subfamily regulates epithelial PCP and synaptogenesis by forming asymmetrical cell-cell contacts.^85^^,^^332^ The C-VANGL complex on 1 cell interacts with the C-FZD complex on the adjacent cell. Notably, A and C subfamily members may not always signal through G proteins,^284^ whereas C receptors can couple to G proteins.^300^

D1 interacts with protein tyrosine kinase 7 (PTK7) on neighboring cells, which positively regulates D1 signaling and requires both NTF interactions and cleavage at the GPS.^86^ These findings shed light on the role of D1 in physiological processes and pathological conditions, such as glioblastoma and other malignancies.^88^^,^^333^^,^^334^

G1 interacts with CD81 in resting NK cells, inhibiting NK effector functions.^279^ Cross-linking of the G1 NTF by anti-G1 mAbs leads to dissociation of the G1-CD81 complex, activating NK cells. G3/GPR97 is a critical component of the PR3-CD177-G3-PAR2-CD16b complex on neutrophils, promoting enzymatic activity of membrane PR3 to cleave PAR2 thus orchestrating PAR2-induced neutrophil activation.^48^ Thus, ENT-mediated aGPCR-GPCR complexes represent another signaling mechanism by which aGPCRs regulate diverse cell-type-specific functions.

In *Drosophila*, the L homolog Cirl is expressed as both a conventional 7TM receptor and a single-pass transmembrane (1TM) variant. These Cirl isoforms appear to heterodimerize (7TM–1TM) via ENT-driven interactions, forming a complex that signals through G*α*_o_ proteins to enable physiological mechanosensing in sensory neurons.^211^ Aside from other long-known extracellular interactions,^335^ mammalian L1 associates with CNTN6 in cultured neurons, inhibiting proapoptotic signaling.^142^

### B Non–cell autonomous signaling

Non–cell autonomy refers to the ability of a gene to influence a phenotype, behavior, or response in cells other than those expressing its gene products (Fig. 6C). An illustrative example of non–cell autonomy is the action of a hormone that is secreted by a gland cell that acts on neighboring (paracrine) or distant target cells (endocrine). Non–cell autonomous signals can also be transmitted contact-dependently (juxtacrine), where the sending cell is in direct contact with the receiving cell, for example, via interacting membrane proteins (Fig. 6C). The structure of aGPCRs lends itself to non–cell autonomous signaling as NTF or other ENT fragment shedding through mechanical forces or proteases can liberate signals from receptor molecules that impact adjacent neighboring or distant cells. In addition, the formation of aGPCR-ligand complexes between cell neighbors can underlie the non-cell autonomy of aGPCR signals (Fig. 6C).^5^^,^^6^^,^^193^ Therefore, for this form of signaling, the terms “N terminus-only,” “7TM-independent,” or “*trans*-signaling” are sometimes used.^190^

For example, shed ENT fragments of E5 and the vasculostatins released from B1 ENTs impact angiogenesis.^99^ Released extracellular fragments of A2^61^ and B1^70^ fragments may control endothelial cell survival by engaging with integrins and/or CD36.^336^

The ENT of L4 promotes epithelial-mesenchymal transition in myofibroblast-like cells and enhances angiogenesis. It is speculated that the NTF is released upon epithelial damage in endothelial extracellular vesicles and aids endothelial sprouting.^337^

L3 is expressed in postsynaptic horizontal cell dendrites in the mouse retina and controls synaptic release from presynaptic cone photoreceptor cells,^209^ although it is unclear whether the NTF of L3 needs to be released for that effect or remains in the mature NTF-CTF receptor complex.

Furthermore, it has been reported that the NTF of G6 can be secreted and bind to cardiomyocytes.^319^ In addition, loss of G6-NTF results in defective compact-wall myocardium in zebrafish.^338^ Based on these data, G6 appears to be expressed exclusively in the endocardium in the heart and to regulate cardiomyocyte behavior in a non–cell autonomous manner and is required for proper heart development.

In *Drosophila*, the aGPCR Mayo is expressed in the midgut, where it regulates the generation of enterocytes by affecting cell lineage decisions. Genetic removal of *Mayo*, however, also causes a drastic increase in cardiac pacing, a site where no Mayo expression is observed, through the dysregulation of potassium levels in the hemolymph by an unknown mechanism.^339^

The Toll-like receptor (TLR) Toll-8/Tollo controls the number of neurons in the cortex of *D. melanogaster* by stimulation of asymmetric neuroblast divisions. This activity is suppressed by the L homolog Cirl, which is expressed in neighboring cortex glial cells, from where it releases its NTF that acts as an inhibiting modulator of the TLR Tollo/Toll-8.^153^ A similar interaction between Cirl and Toll-8 in *trans* is employed to control asymmetric myosin-II polarization at cell-cell boundaries during embryogenesis and tissue morphogenesis in the fly.^154^ Structural, functional, and expression studies in *C. elegans* further support the interaction between subfamily L aGPCRs and TLR.^340^

Also, in *C. elegans*, it was shown that the LAT-1 ENT directly interacts with a ligand of the DSL (Delta, Serrate, and LAG-2) family, thereby modulating the Notch pathway in a non–cell autonomous fashion.^156^ This interaction occurs on the same cell; the signal, however, is relayed to the opposing cell via the Notch receptor. It remains unclear whether the LAT-1 NTF is released in this context, as non–cell autonomy can also emerge from direct interactions of the ENT of an aGPCR with surface-mounted partners without NTF release. For instance, a membrane-bound LAT-1 NTF is sufficient to modulate fertility by controlling sperm guidance, ovulation, and germ cell apoptosis in a non–cell autonomous manner.^219^^,^^294^ The receptor further controls neuronal morphogenesis solely by its NTF.^341^ Another example is the expression of B1 or B3 on glial cells or neurons, which can mediate interaction with RTN4/NoGo receptors on adjacent neurons,^51^ controlling neural network activity.^64^

The C ortholog Flamingo is required for correct photoreceptor axon targeting in the fly’s retina in a non–cell autonomous fashion,^342^ an effect that was also observed for the nematode homolog *fmi-1* in neuronal pathfinding.^343^

*Critical synopsis and outlook: The complex and expanding repertoire of signaling routes in which aGPCRs are embedded is undoubtedly one of the most fascinating—and most consequential—considerations for their prospects as pharmacological targets, a goal that remains unmet to date. Their potential druggability has both benefited from and been complicated by this functional versatility. Insights into the involvement of aGPCRs in multiple signaling pathways and complexes, and even their ability to act as ligands rather than solely as receptors, have broadened the landscape of potential sites for pharmacological intervention through small molecules or biologicals. At the same time, identifying modulators that selectively interfere with only one of several receptor signaling routes may prove particularly challenging.*

## XII Physiology and pathophysiology

### A Organ systems

In this section, we provide a dataset on the anatomical and cellular distribution of all human aGPCR transcripts (section VIII; Fig. 5).

#### 1 Nervous system: Glia and neurons

In vertebrate nervous system development, neural tube closure—the fusion of neural folds at the midline that gives rise to the brain, spinal cord, and neural crest cells—occurs first, followed by neuronal birth and the migration of newborn neurons. Adhesion GPCRs play essential roles in each step, and consequently, their dysfunction is associated with neurological diseases. C1 is required for neural tube closure in mice.^344^ G1 regulates the birth and placement of cortical neurons. Loss-of-function mutations in G1 elicit bilateral frontoparietal polymicrogyria, a devastating human brain malformation,^345^^,^^346^ characterized by neuronal overmigration, ectopias on the brain surface, and myelination deficits.^345^^,^^347^ G1 binds collagen III presented in the pial basement membrane^117^ to initiate the proper stop signal. Similarly, subfamily L aGPCRs also direct neuronal migration using contact repulsion through binding to teneurins and fibronectin leucine-rich transmembrane protein (Fig. 3A).^21^ Following migration, neurons extend axons and dendrites (regulated by subfamily B^64^^,^^327^ and C^83^ members) and identify their partners in neural circuits. L2 facilitates hippocampal partner finding through spatially restricted expression and interaction with Teneurin-3, mediating axonal repulsion from incorrect targets^348^ through G protein signaling.^349^

Synaptogenesis follows brain patterning. Adhesion GPCRs of the L, B, and C subfamilies regulate dendritic spine and synapse formation throughout the CNS, including the hippocampus, cerebellum, cortex, olfactory bulb, and neuromuscular junction.^66^^,^^76^^,^^77^^,^^85^^,^^210^^,^^223^^,^^328^^,^^350^, ^351^, ^352^, ^353^, ^354^, ^355^, ^356^, ^357^, ^358^, ^359^ Intriguingly, these receptors may contribute to a molecular code that specifies distinct synapse types, for example, L1 is implicated in inhibitory synapses, L2/3 in excitatory inputs,^210^^,^^223^^,^^350^ and B3 in the formation of climbing fiber, but not parallel fibers.^77^^,^^353^ Recently described ADGRL-containing transsynaptic adhesion complexes regulated by alternative splicing are required for synapse assembly.^209^^,^^210^^,^^360^^,^^361^ In some cases, lipid metabolites appear to regulate synaptogenesis through F1, which promotes neurite growth and synaptogenesis by binding to its ligand N-docosahexaenoylethanolamine.^108^^,^^109^

Myelination is largely postnatal, involving oligodendrocytes (in the CNS) and Schwann cells (in the peripheral nervous system [PNS]) wrapping hydrophobic membranes around axons, enabling saltatory transmission of action potentials and providing trophic support. G1 functions cell autonomously in oligodendrocyte development and CNS myelination. Its loss-of-function yields CNS myelin deficits in zebrafish,^362^ mice,^363^ and humans.^345^^,^^364^ G1 interaction with microglia-derived transglutaminase acts in this process.^365^ V1 regulates myelination by means of MAG protein stability in myelin-forming cells of the auditory pathway.^366^ In the PNS, G1 regulates myelin formation and maintenance through RhoA and the scaffold plectin.^123^ G6 is indispensable in Schwann cell development and PNS myelination.^367^, ^368^, ^369^ Its binding to collagen IV and laminin-211 initiates a G*α*_s_/cAMP signal to instruct myelin wrapping.^132^^,^^134^

Adhesion GPCR research in glial function is nascent and an area of expected growth. Microglia regulate interneuron development,^370^ synaptic pruning,^114^ and protective response to amyloid deposition^371^ through G1. Astrocytes actively regulate synaptic function by forming tripartite synapses with pre- and postsynaptic terminals. Many aGPCRs are present in astrocytes^372^^,^^373^ and could serve as receptors enabling astrocytic sentinel functions at synapses. For example, astrocytic V1 controls glutamate homeostasis,^374^ and its dysfunction may be linked to epilepsy.^375^

Collectively, these findings establish aGPCRs as essential stage-specific regulators of nervous system development, from neural tube closure through neurogenesis, migration, synaptogenesis, and myelination, whose disruption drives human neurological disease.

#### 2 Sensory systems

Members of the aGPCR family are involved in both the development and physiology of sensory systems. Pathogenic variants of V1 are causative of defects in the auditory and visual systems that are clinically recognized as Usher syndrome type 2 (USH2C), a subtype of human Usher syndrome, the most common form of hereditary deaf-blindness.^376^^,^^377^ USH2C is related to dysfunctions in the sensory cells of the inner ear and eye, where the extraordinarily long ENT of V1 forms fiber links. V1 establishes the ankle-links between neighboring stereocilia, which are essential for the correct hair bundle development of mechanosensory hair cells,^378^ and it forms fibers that stabilize the ciliary pocket of the light-sensitive photoreceptors.^235^ Relatedly, the *D. melanogaster* ADGRL homolog Cirl contributes to the formation and function of photo- and mechano-sensory organs,^195^^,^^211^^,^^379^^,^^380^ and the C homolog Flamingo partakes in visual system development.^342^^,^^381^^,^^382^ Consistent with the subfamily L role in synaptogenesis,^139^^,^^209^^,^^335^ Cirl was identified as a player in the synaptic assembly of photoreceptor cells (R8 axons).^380^ Interestingly, L3 controls synaptic release from cone photoreceptors on horizontal cells in mice non–cell autonomously (Fig. 6C).^218^

**Fig. 8:**
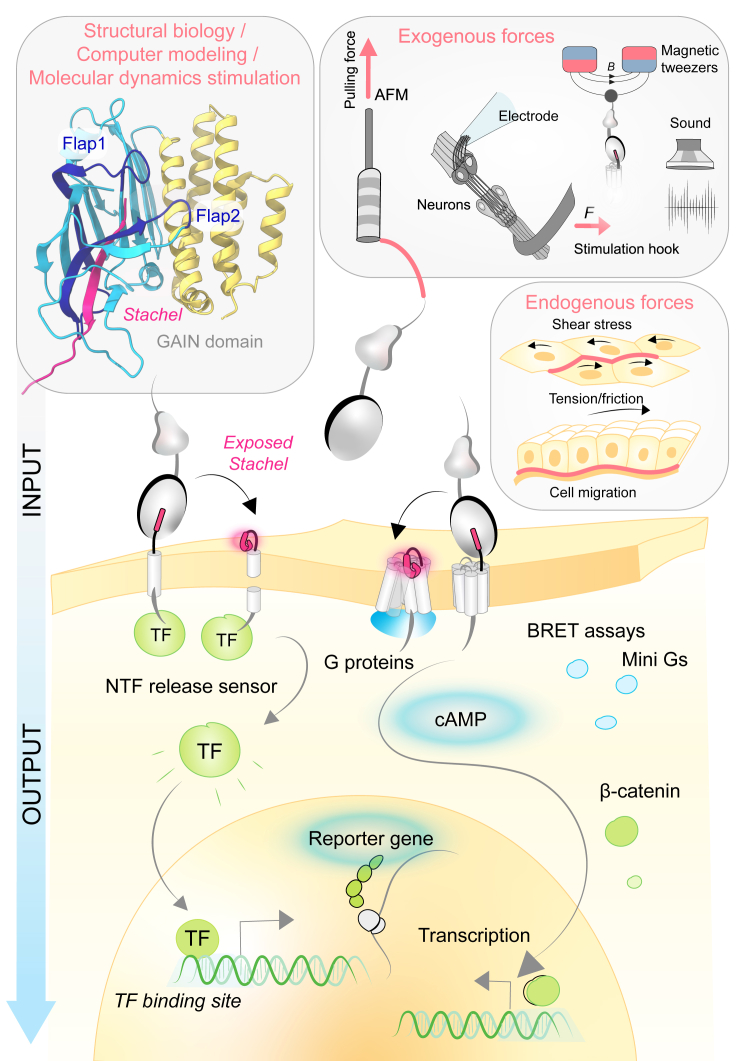
Experimental technologies for aGPCR research. With the finding that aGPCRs are prone to detect adhesive ligands and mechanical stimuli, the field has begun to explore and adapt existing technologies and establish new experimental strategies to mechanically activate aGPCRs and capture the respective mechanoresponse. Some mechanical stimulation paradigms are shown including AFM, magnetic tweezers, acute application of sound stimuli, piezo element-driven pulling forces, and shear stress. Sophisticated computer-based simulations in combination with crystal structures enables directed experimental strategies to investigate structural dynamics and conformational states of specific aGPCR domains. The NTF release sensor (NRS) is a transgenic system that converts the physical NTF separation into visible reporter gene signal and can thus provide quantitative spatiotemporal information of NTF release of a given aGPCR. Technologies such as mini-Gs, cAMP, and BRET assays have been used to track and quantify metabotropic activity of mechanically-stimulated aGPCRs. See section XV.

In the PNS, the subfamily L aGPCR Cirl is important for the physiology of mechanosensory neurons,^195^^,^^379^ providing the initial in vivo characterization of an aGPCR as metabotropic mechanosensory.^383^ Expressed in chordotonal organs of *D. melanogaster*, Cirl sensitizes mechanosensory neurons for the stimulation by proprioceptive, auditory, and tactile stimuli by acting upstream of mechanosensitive transient receptor potential channels (Fig. 8).^379^ Further investigations demonstrated that Cirl modulates responses to innocuous and noxious mechanical stimuli in opposing directions. By sensitizing low-threshold mechanoreceptors and dampening high-threshold nociceptors, Cirl facilitates the separation of mechanosensory signals carrying different physiological information.^194^ Moreover, the antinociceptive action of Cirl suggests a possible target for peripheral analgesic therapy.^194^ Recent work showed that interaction of different Cirl isoforms enables the discrimination between distinct mechanical stimulus intensities through a G*α*_o_/cAMP-dependent signaling route.^211^

In addition, vertebrate aGPCRs serve as critical components in mechanoceptive sensory organs, where they link mechanical force stimulation to ion channel function and intracellular signaling. Equilibrioception, the sense of balance, relies on rapid mechanoelectrical transduction in vestibular hair cells. L2, expressed at the apical surface of utricular hair cells, is required for normal vestibular function in mice.^384^ Loss of L2 impairs balance behavior and abolishes mechanoelectrical transduction currents independent of tip links. L2 conveys sensitivity to force stimuli to hair cells by enhancing the open probability of the ion channel TMC1, thereby driving glutamate release and calcium signaling.^384^ D1 serves a similar function as a key force sensor in utricular hair cells.^385^ D1 converts mechanical stimuli into altered intracellular cAMP signaling via G*α*_i_ signaling, which, in turn, regulates plasma membrane excitability and is coupled to CNGA3 ion channel activity in a subset of hair cells. Reconstitution experiments and structural analyses confirmed the D1-mediated mechanotransduction pathway.^385^

#### 3 Vasculature

The formation of new blood vessels through sprouting angiogenesis is a highly coordinated and multistep collective cell migration event. aGPCRs regulate angiogenesis at various key steps. Endothelial cell-enriched G6 promotes angiogenesis in a cell autonomous manner by stimulating VEGFR2 transcription^386^ or by interacting with and enhancing LRP1 expression.^387^ The stimulation of endothelial cell proliferation or survival contributes to the angiogenic functions of G6 and A2.^61^^,^^386^^,^^387^ In contrast, promotion of endothelial cell migration via small GTPase activation (Fig. 6A), which results in the modulation of cellular adhesion, filopodia, or lamellipodia, seems to constitute a more generic proangiogenic function of aGPCRs. Promigratory aGPCRs include E5, which regulates expression of the Rho GTPase Cdc42,^388^ via its soluble NTF that acts as a chemoattractant for endothelial cells through integrin receptors.^99^ Similarly, G3 and A2 activate endothelial cell migration by acting on the small GTPases Rac, RhoA, or Cdc42.^58^^,^^276^^,^^389^ Rac, in turn, upregulates A2 expression during capillary-like network formation.^390^ A link between A2 and Cdc42 has also been detected in pericytes.^391^ By contrast, L4 promotes angiogenesis without affecting endothelial cell migration, instead participating in VEGF/Notch-dependent tip cell specification.^392^^,^^393^ A2, an essential receptor for brain vascularization,^58^^,^^394^^,^^395^ also acts specifically in tip cells^179^ by regulating the WNT/*β*-catenin-dependent expression of MMP25.^396^

Reflecting their pleiotropic functions in the physiological angiogenic cascade, aGPCRs also affect tumor vascularization. L4^392^^,^^397^ and A2^398^^,^^399^ are enriched in tumor endothelial cells and their genetic inactivation reduces tumor growth and vessel density. G1, E2, and E5 expression in tumor cells stimulates tumor angiogenesis by increasing intratumoral levels of key angiogenic molecules like VEGF and MMP9.^282^^,^^400^ By contrast, B1, B2, and B3 act as antiangiogenic and antitumorigenic factors, at least in part through their NTF, with the NTF of B1 termed vasculostatin (Fig. 6C).^70^^,^^189^^,^^192^^,^^401^^,^^402^

The vascular functions of aGPCRs extend beyond the regulation of angiogenesis. F5 has been implicated in patterning the retinal vasculature^403^ and genetically interacts with L4 to shape the aortic arch arteries and the cardiac outflow tract, functions that may not be endothelial-autonomous.^404^ F5 and L2 contribute to flow and fluid shear stress mechanotransduction.^405^^,^^406^ C1 controls lymphatic endothelial cell movements during valve formation by inhibiting the maturation of adherens junctions.^407^ L2, A2, and G6 contribute to the control of vascular permeability,^408^the latter 2 especially at the blood-brain barrier.^387^^,^^408^^,^^409^ L4 overexpression triggers endothelial-mesenchymal transition, linked to an increase in chemokine and cytokine expression,^410^ while its silencing markedly affects endothelial metabolism.^411^

#### 4 Lymphatic vessels

The lymphatic vasculature plays key roles in interstitial fluid balance, immune surveillance, and lipid absorption. Lymphatic endothelial cells (LEC) exhibit unique mechanosensitive properties and specialized cell-cell junctions, enabling them to adapt their permeability and regulate lymph flow.^412^, ^413^, ^414^ These features, along with their “puzzle” morphology, make LEC an attractive model for exploring the function of aGPCRs. To date, C1 and G3 have been studied in human lymphatics.^389^^,^^407^^,^^415^, ^416^, ^417^, ^418^, ^419^ C1 regulates lymphatic valve formation through endothelial cell rearrangements and junction maturation via VE-cadherin stabilization.^407^ In disease, C1 is implicated in hereditary lymphedema, a lymphatic vascular disorder characterized by chronic swelling.^415^^,^^416^ However, the effects of C1 lymphedema-associated genetic variants on receptor function remain unclear. Similarly, G3 regulates cytoskeletal organization, cellular adhesion, junctional integrity, and migration of LECs via activity modulation of the small GTPases Cdc42 and RhoA.^389^ Other aGPCRs, including A2, L4, G1, E5, and F5, are abundantly expressed in human LEC (Fig. 5).^389^^,^^420^^,^^421^ However, their specific functions in lymphatic biology remain unexplored.

Given the critical role of aGPCRs in other conditions such as cancer metastasis and obesity, where lymphatic dysfunction is implicated,^321^^,^^422^ exploring the role of aGPCRs in lymphatics will have broad implications.

#### 5 Skin

Human single-cell RNA sequencing (scRNA-seq) data show moderate levels of C1, C2, F4, G1, L1, L2, and V1 in basal and suprabasal keratinocytes that form the epidermis, the squamous (cornified) epithelium of the skin (Fig. 5). These transcripts are also present in squamous (uncornified) epithelial cells covering other organs, such as the oral cavity, tongue, esophagus, and vagina. Among these, only F4 has been confirmed at the protein level in humans.^247^ A few basal and all suprabasal, uncornified keratinocytes express F4. In psoriatic skin, F4 expression is diminished, suggesting its involvement in epidermal differentiation. Deletion of F4 reduced the number of keratinocyte layers in organotypic cocultures and abolished expression of keratin 1. Endogenous F4 exhibits unexpected close intracellular colocalization with keratin 1.^247^ In mice, not only F4 is present in the skin and other squamous cornified epithelia but also F2,^423^ suggesting specific functions in these epithelia. Notably, F2 is completely missing in humans (Fig. 5). Members of the AC subfamily are, among others, expressed in a wide range of epithelia in human and mouse, where especially C1 controls the establishment of epithelial PCP,^424^ a process that polarizes epithelial cells within the plane of a tissue. For instance, mammalian C1 is responsible for the coordinated alignment of hair follicles across the skin surface.^79^

#### 6 Heart

Adhesion GPCRs are expressed in the heart during development (F5^404^, G6^319^^,^^425^^,^^426^, L2^427^^,^^428^, and L4^404^) and adulthood (F5^404^, G6^319^^,^^425^, L2^428^, L4^404^, and G1^429^^,^^430^) in endocardial cells (G6^319^, F5^404^, and L4^404^), vascular endothelial cells (F5^404^ and L4^404^), cardiomyocytes (G1^429^^,^^430^ and L2^427^^,^^428^), and cardiac cushions (L2^427^) (Fig. 5).

KO studies have demonstrated their relevance. Analysis of global *L2*^*KO*^ mice, which are embryonic lethal, and *L2* knockdown in chicken and stem cells indicates that L2 specifies cardiac lineage commitment^431^^,^^432^ and controls endothelial-to-mesenchymal transition within cardiac cushions.^427^ Postnatally induced cardiomyocyte-specific *L2*-KO caused dilated cardiomyopathy, serious arrhythmia, and death, which could be rescued with p38-MAPK activators.^433^ Similarly, global G6 deletion is embryonic lethal, causing a thinned ventricular wall, hypotrabeculation, bradycardia, arrhythmia, abnormal mitochondria, and/or circulatory failure.^319^^,^^426^^,^^434^ Recent data suggest these phenotypes may be secondary to placental defects.^434^ However, another study reported normal placenta,^426^ and data in zebrafish (no placenta) showed that G6 is required for trabeculation.^319^^,^^338^ Increased perinatal lethality, ventricular septum defects, and/or malformed large vessels^404^ have been reported for global double *L4/F5* double KO, which alone causes no or a mild phenotype.^435^ Surprisingly, similar to G6,^434^ endothelial-specific deletion of *L4/F5* did not cause a heart phenotype. These data indicate that aGPCRs in different cardiac cell types play important roles in heart development, but more work is required to resolve current controversies.

Studies also emphasize the importance of aGPCRs in adulthood. Cardiomyocyte-specific *G1*-KO and global *L4*-KO each caused little to no cardiac phenotype in unstressed hearts but an accelerated cardiac dysfunction upon chronic pressure overload.^430^^,^^436^ Cardiomyocyte-specific *G1*-KO mice displayed increased LV dilation and heart weight with no increase in wall thickness, indicating impaired hypertrophy, although cardiomyocyte cross-sectional area was only slightly and not significantly decreased. Impaired hypertrophy would be in line with previous reports that *G1*-KO/knockdown^437^^,^^438^ or PCBP2-mediated G1-mRNA degradation^438^ attenuated induced hypertrophy in skeletal muscle or neonatal cardiomyocytes. In contrast, *L4*^*KO*^ mice displayed increased pressure overload-induced LV wall thickness, fibrosis, and cardiomyocyte cross-sectional area.^436^ Collectively, there remains uncertainty about which signaling pathways and cellular behaviors are controlled by aGPCRs in the heart.

#### 7 Lung

Transcripts of 12 aGPCR genes (*L1*, *L2*, *E5*, *A3*, *C1*, *C2*, *D1*, *F1*, *F5*, *G1*, *G6*, and *V1*) are expressed in specialized epithelial cells throughout the respiratory tract including ciliated cells, basal respiratory cells, club cells, ionocytes, alveolar type 1 (AT1), and alveolar type 2 (AT2) cells (Fig. 5). However, a role for most of these receptors in lung function has not been reported. F5 is the most abundantly expressed aGPCR in AT1 and AT2 cells, and several research groups generated loss-of-function mice to determine its role in vivo. The most striking phenotype in *F5*^*KO*^ mice was a marked accumulation of pulmonary surfactant in the distal lung,^112^^,^^266^^,^^267^ resulting in progressive immune cell–mediated alveolar simplification.^112^^,^^435^^,^^439^, ^440^, ^441^ Mechanistically, AT2 cell-specific F5 expression regulates surfactant secretion and uptake in AT2 cells via G*α*_q/11_ signaling.^281^^,^^440^ Coimmunoprecipitation studies identified surfactant protein D as a putative F5 ligand.^112^ However, functional ligand-dependent receptor activation studies have not been reported.

Several aGPCRs have emerged as hits from unbiased screening approaches and genome-wide association studies in chronic lung diseases. For example, in idiopathic pulmonary fibrosis, E5 has been proposed as a marker for quiescent fibroblasts^442^ and reduced G1 expression was observed in fibroblasts cultured from IPF patients.^443^ In chronic obstructive pulmonary disease (COPD), single nucleotide polymorphisms (SNPs) in the *G6* locus have been reproducibly associated with reduced lung function, a pathological hallmark of tissue destruction in COPD.^444^, ^445^, ^446^, ^447^ In addition, decreased lung G6 expression in COPD patients supports a common SNP as being causal for disease.^445^ In the context of idiopathic pulmonary arterial hypertension, G6 expression was increased in human airway smooth muscle cells,^448^ and novel SNPs suggest potential causal variants affecting G6 expression and/or function.^449^ Furthermore, TIA-mediated activation of G6 in human airway smooth muscle cells suggested a role in cell proliferation and airway remodeling.^449^ Finally, *G6* knockdown in iPSC-derived human AT2 cells alters cellular responses to injury, demonstrating a role for this receptor in postinjury epithelial cell repair in the distal lung.^450^

In summary, future studies are necessary to elucidate the function of many aGPCR family members expressed in pulmonary epithelial and other respiratory tract cell types, including fibroblasts, endothelial cells, and smooth muscle cells.

#### 8 Musculoskeletal system

The musculoskeletal system, comprising bones, muscles, tendons, and joints, is essential for structural support, movement, and metabolism. Dysfunctions in this system can lead to conditions such as osteoporosis, muscle atrophy, and arthritis. aGPCRs have emerged as key regulators of musculoskeletal function, influencing development, repair, and mechanotransduction.^451^

Genetic studies link *D1* and *G6* to human height, weight, and skeletal frame size.^1^^,^^452^^,^^453^ Notably, G6 maintains growth plate homeostasis via the PTHrP/IHH pathway, with mutations implicated in adolescent idiopathic scoliosis.^454^, ^455^, ^456^, ^457^, ^458^ Similarly, D1 acts as a membrane receptor for androgens, activating the G*α*_s_/cAMP/PKA pathway to enhance muscle strength and growth. The development of the selective androgen analogs, such as AP503, targeting D1 without binding nuclear androgen receptors, offers a promising treatment for muscle atrophy with minimal side effects, broadening the scope of androgen therapy.^88^ D1 has recently been identified as a regulator of bone formation, acting simultaneously on osteoblasts and osteoclasts. The receptor is activated by mechanical forces and interaction with its ligand PTK7.^254^^,^^459^

Beyond these roles, G1 plays a significant role in protein synthesis and muscle hypertrophy. Its function is driven by PGC-1*α*4 or mechanical loading, which activates the G*α*_12/13_ pathway.^437^ Interestingly, G1 expression is upregulated during early differentiation of human cultured myoblasts. Although G1-deficient myoblasts show impaired fusion in vitro, *G1*^*KO*^ mice exhibit no overt phenotype, suggesting that compensatory mechanisms may mitigate its loss during muscle development.^460^ Furthermore, B1 and B3 promote myoblast fusion. B1 promotes myoblast fusion by recognizing phosphatidylserine on apoptotic cells through its thrombospondin repeats, initiating the ELMO/Dock180/Rac1 pathway.^326^ Similarly, B3 interacts with ELMO to activate Rac1 (Fig. 6A), driving embryonic myoblast fusion processes.^277^

Other aGPCRs also contribute to musculoskeletal function. E5 deletion results in abnormal sarcoplasmic reticulum structure, although skeletal muscle function remains unaffected, indicative of its impact on structure rather than function.^249^ Meanwhile, V1 has been identified as a regulator of bone density in humans and mice, linking it to osteoporosis susceptibility.^461^

Collectively, these findings solidify the role of aGPCRs as mechanosensors and signaling hubs in the musculoskeletal system, with significant implications for treating conditions like adolescent idiopathic scoliosis, osteoporosis, and muscle degeneration.

#### 9 Immune system and spleen

The immune system-associated aGPCRs are primarily clustered in 2 subfamilies, E and G (Fig. 5). Notably, several E and G receptors exhibit restricted expression within specific immune cell populations, making them selective biomarkers for distinct immune cell subsets.^253^ For example, E1 (F4/80) serves as a well established marker for mouse tissue macrophages, whereas E1 was identified as a specific marker for human eosinophils.^462^, ^463^, ^464^ Individual aGPCRs are key markers of human mature polymorphonuclear granulocytes (E3) and pancytotoxic lymphocytes, as well as microglia (G1).^251^^,^^465^^,^^466^ Furthermore, aGPCRs have unique pathophysiological roles in regulating innate and adaptive immune responses, owing to their dual functions in cell adhesion and signaling.^467^ Thus, myeloid-restricted aGPCRs, such as E2 and G3, are involved in necrotic-like cell recognition and specific activation of protease-activated receptor 2, respectively, leading to inflammatory activation of macrophages and neutrophils.^48^^,^^91^^,^^313^ G1 is an NK-cell inhibitory receptor that suppresses cellular cytotoxicity, cytokine production, and cell migration.^251^^,^^279^^,^^468^ E5-deficient mice exhibited mild granulocytosis and enhanced antibacterial activity, whereas E1 was found to promote regulatory T cell–mediated peripheral immune tolerance.^469^^,^^470^

Compelling evidence supporting the role of aGPCRs as metabotropic mechanosensors has initially emerged from studies on immune aGPCRs. The E2^C492Y^ missense mutation, altering the GAIN domain of the receptor (Fig. 4), results in a less stable receptor complex that readily releases its NTF upon vibratory stimulation in the presence of its ligand, triggering excessive histamine release by mast cells to cause a rare dermal allergic disorder known as vibratory urticaria.^89^ Similarly, E5 functions as a shear stress-dependent mechanosensor on leukocytes by interacting with CD55 (Fig. 3C).^471^ Notably, E5-mediated mechanosensing of CD55 on red blood cells is crucial for maintaining proper compartmentalization, homeostasis, and adaptive immune functions of type 2 conventional dendritic cells and marginal zone B cells in the spleen.^94^^,^^97^ Likewise, G1 plays a critical role in platelet shape change during hemostasis by acting as a specific collagen receptor that responds to shear forces in blood circulation.^286^

In addition, some aGPCRs are expressed in several differentiation stages of hematopoietic stem and progenitor cells, suggesting their potential involvement in normal hematopoietic development and leukemogenesis.^472^, ^473^, ^474^, ^475^ Finally, immune aGPCRs, including G1, E2, and E5, are implicated in various (patho)physiological processes and immunological disorders, highlighting their role in immune (dys)function.^476^, ^477^, ^478^, ^479^ For instance, E2 is temporarily upregulated on neutrophils in the posttraumatic course,^479^^,^^480^ and its expression is higher on neutrophils from sepsis compared with noninfectious patients with a systemic inflammatory response syndrome.^478^ In conclusion, specific aGPCRs are actively involved in modulating diverse immune responses, with a crucial contribution to immune dysfunction and disorders.

#### 10 Kidney

Numerous aGPCRs are expressed in the murine metanephric kidney in a variety of cell types (Fig. 5), including glomerular endothelial cells (F5^481^, G1^482^^,^^483^, G3^484^, and possibly L4^404^^,^^485^), mesangial cells (G3^484^), parietal epithelial cells (G6^486^), and podocytes (A2^487^, C1: embryo^488^, and G3^484^), as well as cells of the proximal tubules (B1^489^, C1^488^, G1^252^, and G3^484^), distal tubules (B1^489^ and G1^252^), collecting ducts (C1: embryo^488^, F5^490^^,^^491^, G6^425^^,^^486^, G3^485^, and F1^485^), and renal pelvis/urothelium (F1^423^^,^^492^ and G6^325^^,^^486^). This expression pattern has in part also been observed in zebrafish^493^ and humans^494^ (F1^492^, G1^252^, and G6^486^).

Transcriptomic and proteomic data suggest another 7 aGPCRs to be expressed in the murine nephron.^495^ Notably, polycystin-1, the protein product of the *PKD1* gene whose mutation causes autosomal dominant polycystic kidney disease, can be regarded as an atypical aGPCR^193^^,^^496^ containing a GAIN domain and being responsive to a TIA (Figs. 4 and 6A).^497^^,^^498^ A variety of kidney diseases are associated with altered expression levels or patterns of a number of aGPCRs^494^ (A2^487^, G1^483^, G3^484^, and G6^499^). Although these descriptive data indicate that aGPCRs play an important role in kidney development and physiology, our understanding of their relevance is limited. C1 is the best-characterized aGPCR in kidney development with *C1*^*KO*^ mice exhibiting uretric bud branching defects, growth retardation, dilated cortical tubules, and mitotic spindle misorientation.^500^ Adult global *F5*^*KO*^ exhibit impaired function with compromised basement membrane composition and morphological defects in the glomeruli.^481^ Kidney-specific *F5*^*KO*^ have a significantly reduced urine pH, attributable to an increased V-ATPase accumulation.^490^ Combined *F5;L4*^*DKO*^ results in ∼50% perinatal lethality. Alive-born *F5;L4*^*DKO*^ mice exhibit loss of endothelial fenestration and fusion of podocyte foot processes, resulting in proteinuria, uremia, and death at 4 weeks.^404^ However, endothelial-specific *F5;L4*^*DKO*^ animals display no renal defects. Also, global *F1*^*KO*^ does not result in overt defects.^323^ Finally, it has been shown that deletion of aGPCRs can be protective (G3: acute kidney injury,^484^ hypertensive nephropathy^501^; and G1: diabetes^483^) or detrimental (A2: diabetes^487^) in kidney disease.

#### 11 Pancreas

Adhesion GPCRs are increasingly recognized as regulators of glucose metabolism, acting at multiple levels including pancreatic islets, insulin-sensitive tissues, and local inflammatory environments. In human pancreatic islets, expression of several aGPCRs, such as *A2, A3, B3, C2, C3, E5, F1, F4, F5, G1, L1, L2,* and *L4*, has been reported (Fig. 5).^502^^,^^503^ Their roles range from pancreatic development to insulin secretion. For instance, G1 enhances *β*-cell mitochondrial function and insulin release via collagen III-mediated ECM sensing, although its deletion does not impair glucose tolerance in mice.^504^, ^505^, ^506^ L1 and L3 exert opposing effects on insulin secretion,^215^ whereas B3, via C1ql3 activation, suppresses insulin secretion by lowering cAMP levels.^507^

#### 12 Metabolism, fat, and liver

Activation and signaling of aGPCRs change the physiological function of adipocytes, hepatocytes, and myocytes, thereby influencing whole-body energy homeostasis. Indeed, it has been demonstrated that more than one-third of aGPCRs are among the most abundantly expressed GPCRs in white adipose tissue.^508^ Several members, namely A2, A3, D1, G1, G2, G6, L2, and F5, are involved in preadipocyte differentiation into mature adipocytes.^508^, ^509^, ^510^, ^511^ Besides, aGPCRs can also directly influence adipocyte function: G2 activation increases lipolysis^508^ and modulates insulin-stimulated glucose uptake.^508^ Regulation of insulin sensitivity has also been found for F5.^113^^,^^509^

Additionally, aGPCRs can affect the thermogenic program as documented for B3^512^ and specifically impact adipocytes as observed for A3, which is highly expressed in human adipocytes and murine brown fat. A3 knockdown in mice reduces uncoupling protein 1 and other thermogenic markers and exacerbates obesity, whereas A3 overexpression induces beige adipocyte biogenesis and increases energy expenditure, improving metabolic homeostasis.^513^ G2 expression is upregulated when brown adipose undergoes “whitening,” suggesting that it negatively regulates brown/beige fat thermogenic capacity.^514^ In a KO mouse model, it was shown that B3 reduces thermogenesis; however, it is still uncertain if this is mediated by its adipocyte-specific function.^512^

Differential expression of several aGPCRs in the liver has been reported in hepatocytes (*A3, G6, G7, G8, F5, L2,* and *L4*) and cholangiocytes (*A3, E5, G1, G2, G6, F1,* and *L2*) (Fig. 5).^515^ However, their functions remain incompletely understood. Emerging evidence links them to liver metabolism and injury. As such, F5 affects lipid metabolism^458^ and ferroptosis in sepsis-induced liver injury.^516^ Furthermore, its deletion alleviates sepsis- and acetaminophen-induced liver injury.^517^ F1 regulates hepatic lipid metabolism,^518^ and its absence mitigates steatosis and fibrosis in mice.^519^ C2 affects LDL cholesterol levels^520^ and lipid accumulation via the modulation of reactive oxygen species.^521^ G1 sensing of 17*α*-hydroxypregnenolone protects against ferroptosis-induced liver injury.^522^

Taken together, these findings highlight critical roles for aGPCRs in metabolism and energy homeostasis.

#### 13 Gastrointestinal tract

A closer examination of the human scRNA-seq data for aGPCRs (Fig. 5) reveals that several aGPCRs (*A3, E5, G1, G4, G6,* and *G7*) exhibit moderate expression across various (glandular) epithelial cell types in the gastrointestinal tract. Additionally, qRT-PCR analysis of 12 rat gastrointestinal segments identified 28 aGPCRs, some of which displayed restricted expression patterns along the gastrointestinal tract axis, likely associated with specific gut functions.^523^ Similarly, an analysis of 4 mouse gastrointestinal canal subsegments detected low-abundance aGPCRs, with *B1-3* localized in the muscle-myenteric nerve layer and *G4* found in the duodenal, jejunal, and ileal mucosa.^524^

Several aGPCRs are specifically expressed in specialized cell types of the epithelial layer. Human E5 localizes at adherens junctions, interacting with *β*-catenin,^274^ although it is located intracellularly in colorectal cancer.^525^ Human G1 colocalizes with pepsinogen at the gastric gland base and may support colonic stem cell expansion.^121^^,^^252^ Mouse G2 has been reported to be selectively expressed in mature chemosensory cells,^526^ whereas human G4 is enriched in enterochromaffin and neuroendocrine carcinoma cells, serving as a potential biomarker.^527^ Furthermore, G4 pentraxin-domain facilitated receptor homodimerization has been suggested to be critical to endogenous activation, although its function remains unknown (Fig. 3D).^169^

In gastrointestinal tract development, the aGPCR Mayo of *D. melanogaster* shows midgut hyperplasia, hyperkalemia, and tachycardia.^339^ In mice, overexpression of E5 causes a dose-dependent megaintestine with normal microscopic morphology, providing a model for postnatal intestinal growth.^528^ Expression of E5 and B1 in the colon was found to similarly protect mice from dextrane sulfate sodium -induced colitis, but likely via different mechanisms.^529^ G7, found predominantly in intestinal epithelial cells in mice and humans, may aid in nutrient absorption.^530^

Although the cellular distribution in the gastrointestinal tract of certain aGPCRs has been recently characterized and offers insight into their roles in (patho)physiological processes, several receptors like A3 and G6 remain largely uncharacterized, highlighting the need for a comprehensive investigation of their protein expression patterns and gastrointestinal tract function.

#### 14 Female reproductive system

Congenital abnormalities of the female reproductive tract affect 10% of women, often with severe consequences.^531^ aGPCRs are crucial in the development of these tissues, aiding in cell positioning and organization.^532^^,^^533^ They facilitate cell-ECM interactions, essential for tissue integrity and function. Additionally, aGPCRs are involved in developmental signaling pathways, including the WNT signaling pathways, which regulate tissue morphogenesis and differentiation.^153^^,^^534^, ^535^, ^536^, ^537^, ^538^

The *C. elegans* L homolog LAT-1 is important for the hermaphrodite nematode ovulation, sperm guidance, and germ cell apoptosis, through a *trans*-mechanism independent of its CTF (Fig. 6C).^156^^,^^219^

In mice, half of *A3*-deficient females fail to develop a vaginal opening during puberty; correspondingly, A3 is expressed in the female urogenital system, with the highest prepubertal expression and with a rapid drop after sexual maturation.^532^ *A3* deficiency impairs estradiol-dependent vaginal canalization by unbalancing apoptotic regulators, potentially driven by the PCP WNT pathway,^532^ an established pathway for A3.^63^^,^^262^^,^^271^^,^^273^^,^^539^, ^540^, ^541^, ^542^ Aligned with the idea that A subfamily members interact with the WNT pathway is the finding that reduced postpubertal A3 expression coincides with upregulated A2 expression—also active in WNT pathway signaling—in the female reproductive tract.^532^ Curiously, also G protein-dependent signaling (Fig. 6A), although weak, was recently described for A3.^539^

Birds display high expression of G1 and its ligand, collagen III, in the Müllerian duct, which gives rise to the fallopian tubes, uterus, and upper vagina in human females, and the oviducts in the chick (Fig. 7).^533^ Knockdown of *G1* results in truncated ducts, suggesting a central role of G1 in the developmental elongation process, potentially via PAX2 signaling.^533^ Intriguingly, G1 also modulates cellular *β*-catenin levels and regulates the canonical WNT pathway, possibly via its ligand transglutaminase-2.^543^

G2 and G6 are both linked to uterus and placental development during pregnancy.^434^^,^^544^ G2 regulates the decidualization of endometrial stromal cells during implantation and early gestation, likely involving the PI3K/Akt/mTOR signaling pathway.^545^ For G6, G protein signaling by progesterone promotes breast cancer^136^ and likely also female reproductive system development. Likewise, D1, expressed in the murine oviduct, regulates ductal fluid flow and embryo transit, possibly via Plxdc2.^87^

aGPCRs involved in female reproduction have also been linked to male fertility, for example, A3,^546^ G1,^547^ and G2,^548^ supporting their fundamental roles in development.

#### 15 Male reproductive system

aGPCRs involved in the male reproductive tract are A2^314^ A3^546^ G2 is highly expressed in the male reproductive system, primarily localized in the efferent ducts and proximal epididymis.^549^^,^^550^ G2 plays a critical role in sperm maturation. *G2*^*KO*^ mice exhibit reduced sperm count or motility, and morphological abnormalities (such as head defects)^551^ while also causing downregulation of genes related to sperm maturation in the epididymis (including cystatin, lipocalins, *β*-defensins, Adam28, Crisp1, and Enpp2).^548^ G2 couples with the anion channel CFTR in a G*α*_q_-dependent manner to regulate Cl^-^ and pH homeostasis. The G2-G*α*_q_ signaling axis maintains the baseline outward rectifying current of CFTR, which is essential for fluid reabsorption and sperm maturation. G2, CFTR, *β*-arrestin1, and G*α*_q_ form a supercomplex localized to the apical membrane of nonciliated cells, acting as a regional signaling hub to regulate fluid reabsorption and ion homeostasis (Fig. 6A, B).^314^

Clinical studies have identified various *G2* mutations (such as *p.Glu516Ter, p.Leu668ArgfsTer21, p.Arg814Ter,* and *p.Lys818Ter*) associated with male infertility and closely linked to congenital bilateral absence of the vas deferens. These mutations result in the deletion or truncation of the 7-transmembrane domain, disrupting the coupling of G2 with downstream G*α*_q/_G*α*_s_ proteins and *β*-arrestins (Fig. 6A).^552^, ^553^, ^554^

L2 interacts with its ligand LRG1 to ameliorate vascular and neurological abnormalities and restore diabetic erectile function.^150^

### B Diseases

Adhesion GPCRs are recognized in numerous disease contexts by contributing to monogenic diseases and complex pathologies—including cancer, diabetes, and neurological disorders—and by targeting structures for viral components.

#### 1 Cancer

E5 was the first aGPCR reported to be regulated during tumorigenesis.^555^ Over the past decades, many more aGPCRs have been implicated in cancer. Members of all 9 aGPCR subfamilies are now considered to partake in tumorigenesis and/or the tumor microenvironment, influencing cancer growth and metastasis. Somatic mutations in aGPCRs have been detected in multiple cancers, but whether they drive or accompany tumorigenesis remains unclear. Alterations in aGPCR expression and/or signaling can contribute to key oncogenic processes (cancer hallmarks), such as tumor cell growth (proliferation and survival), motility and spread (migration, invasion, and metastasis), access to vasculature (vascular cooption, angiogenesis, and vasculogenesis), tumor-promoting inflammation (immune cell recruitment), immune escape, and therapeutic resistance. Below is a nonexhaustive selection of key examples of aGPCR involvement in cancer:

##### a Tumor progression and growth

Adhesion GPCRs can either suppress or promote tumor growth depending on their expression level and signaling context. Several studies have profiled the aGPCR expression in individual malignancies and assessed their prognostic impact.^100^^,^^556^, ^557^, ^558^, ^559^, ^560^, ^561^

For example, G1 is overexpressed in acute myeloid leukemia, colorectal, and prostate cancers and sustains in vivo tumor growth, invasion, and/or therapeutic resistance.^562^ Conversely, the interaction of G1 with its ECM ligand, transglutaminase-2, blocks the growth and metastasis of melanoma^166^ and exerts tumor-suppressive functions in glioma.^563^

Similarly, F1 in HER2^+^ breast cancer promotes tumorigenesis but switches to a tumor-suppressive role upon interacting with laminin-111, leading to decreased receptor signaling, tumor cell senescence, increased HER2 expression, and enhanced sensitivity to anti-HER2 therapies.^111^

D1, a G*α*_s_-coupled aGPCR known to be regulated by ESYT1 in a Ca^2+^-dependent manner, is allosterically activated by its ligand PTK7 to promote glioblastoma growth and brain invasion.^86^^,^^280^^,^^333^^,^^564^^,^^565^ D1 has also been implicated in a few other malignancies, including lung and gastric adenocarcinoma, and leukemia,^559^^,^^566^, ^567^, ^568^ although its exact function in those settings has not been clearly determined.

Several members of the E subfamily, most notably E5, have also been implicated in a plethora of malignancies, where they contribute to cancer stem cell maintenance, tumor cell migration, invasion, apoptosis, proliferation, and response to therapy.^100^^,^^569^, ^570^, ^571^, ^572^, ^573^, ^574^, ^575^, ^576^, ^577^, ^578^, ^579^, ^580^, ^581^, ^582^, ^583^, ^584^, ^585^, ^586^, ^587^, ^588^, ^589^, ^590^, ^591^, ^592^, ^593^

A2 was shown to regulate glioblastoma proliferation through effects on mitotic assembly and progression.^594^ A3 was identified as a marker of stem cells in breast tissue, which are greatly expanded upon oncogenic transformation.^595^

B family members are transcriptionally downregulated or frequently mutated in various cancers, including brain, breast, and lung, suggesting a tumor-suppressive role.^402^^,^^595^, ^596^, ^597^, ^598^ In brain malignancies, B1 undergoes epigenetic silencing, and reactivating its expression inhibits tumor cell proliferation, angiogenesis, and tumor growth in vivo, further supporting its tumor-suppressive function.^329^^,^^599^

##### b Tumor cell migration/invasion and metastasis

Many aGPCRs, such as E1, E2, E5, L1, and G1, mediate ECM interactions and/or promote epithelial-to-mesenchymal transition,^102^^,^^587^^,^^600^, ^601^, ^602^, ^603^, ^604^, ^605^, ^606^, ^607^, ^608^, ^609^, ^610^, ^611^, ^612^, ^613^, ^614^ whereas L4 is associated with endothelial-to-mesenchymal transition.^410^ By triggering changes in cell adhesion and motility, these receptors enable cancer cells to adopt invasive mesenchymal-like properties, promoting metastasis.

A2 expressed on pericyte-like cells derived from lung adenocarcinoma stem cells enables them to initiate brain metastases through *trans*-endothelial migration.^556^ This effect of A2 is mediated by its ability to act as a WNT coreceptor, thus promoting canonical WNT7/b-catenin signaling.^59^^,^^261^^,^^601^

L1-3, whose expression is induced by nuclear androgen receptor signaling, promote prostate cancer growth.^615^ A similar effect is noted for L3 in urothelial cancer.^616^ Cancer-linked mutations affecting the L3 GAIN domain impair its adhesion functions and G*α*_13_ signaling, leading to altered cell motility and cytoskeletal organization that may contribute to tumor progression.^617^ L3 knockdown reduces bladder cancer cell migration.^618^

G1 localizes to the leading edge of glioblastomas, supporting cell adhesion and invasive behavior of tumor cells.^619^ G1 also promotes hepatocellular carcinoma metastasis.^477^^,^^617^ L4 promotes migration in glioblastoma, colorectal cancer, retinoblastoma, and neuroblastoma.^620^, ^621^, ^622^, ^623^

C1 promotes migration in ovarian cancer,^624^ whereas C3 knockdown in lung adenocarcinoma^625^ and neuroendocrine prostate cancer^626^ suppresses migration.

##### c Immune evasion

Several aGPCRs are expressed in immune lineages of the tumor microenvironment. Members of the E subfamily (E1, E2, E3, and E5) can modulate the immune microenvironment by influencing immune cell infiltration to the tumor site and controlling the function of tumor-associated immune cells.^100^^,^^577^^,^^627^, ^628^, ^629^, ^630^, ^631^

L4 overexpression in breast cancer cells is linked to an immunosuppressive tumor environment, marked by fewer cytotoxic T cells and more M2-like macrophages.^632^

G1 is upregulated in exhausted, tumor-reactive T cells in cholangiocarcinoma, renal cell carcinoma, lung cancer, and the bone marrow in AML,^633^, ^634^, ^635^, ^636^, ^637^ suggesting a broader role in immune suppression.

B3 regulates F-actin in T cells, reducing traction force and impairing cytotoxicity in melanoma and colorectal cancer.^638^ These receptors may promote immune evasion by recruiting immune suppressor cells to the tumor or by inhibiting antitumor immune responses.

##### d Angiogenesis

Some aGPCRs, including L4^392^^,^^393^^,^^639^ and G1,^562^ are involved in tumor angiogenesis. By stimulating the tumor’s ability to form blood vessels, aGPCRs directly contribute to cancer progression and are potential therapeutic targets.

The angiogenic actions of aGPCRs can be exploited therapeutically. For example, the use of anti-L4 antibodies or scFVs in glioblastoma xenografts normalizes tumor vessels and improves survival in comparison to untreated controls or those treated with anti-VEGF antibody (bevacizumab) alone.^640^^,^^641^ G1 can inhibit VEGF-induced angiogenesis in melanoma,^282^ whereas B1 acts as a negative regulator of angiogenesis through N-terminal cleavage events that release vasculostatins.^192^ In addition, E2 and E5 may also play a role in regulating tumor angiogenesis.^400^ Finally, A2 expressed on tumor endothelial cells is necessary for VEGF-induced tumor angiogenesis.^399^

#### 2 Type 2 diabetes

Adhesion GPCRs regulate systemic insulin sensitivity through actions in adipose tissue and muscle. F5 mediates insulin-sensitizing effects of the hepatokine FNDC4 in adipose tissue. Its deletion impairs adipokine release and promotes systemic insulin resistance.^113^^,^^509^ A3, activated by the flavonoid hesperetin, also contributes to improved insulin sensitivity in adipose tissue.^513^ Although aGPCRs are highly expressed in myoblasts, their function in skeletal muscle insulin sensitivity remains unexplored.

Importantly, aGPCRs also influence diabetes pathophysiology through immune-metabolic crosstalk. G3 expression in adipose tissue macrophages promotes proinflammatory signaling; its deletion dampens macrophage activation and reduces obesity-induced adipose inflammation.^642^ These findings collectively point to aGPCRs as context-dependent regulators of insulin secretion, sensitivity, and inflammation—key elements in diabetes development and progression.

#### 3 Viral infection

Viruses either encode GPCRs (viral vGPCRs)^643^^,^^644^ or exploit host GPCRs^645^ to manipulate cellular processes and enhance their control over the host environment. In response, hosts continually adapt their GPCRs to counter these viral strategies. Consequently, it is not surprising that viruses regulate or interact with aGPCRs, given their crucial roles in cell signaling,^293^ maintaining host physical barriers,^79^^,^^387^^,^^646^, ^647^, ^648^ and immune surveillance.^167^^,^^253^^,^^467^^,^^468^^,^^649^^,^^650^

During severe acute respiratory syndrome coronavirus 2 (SARS-CoV-2) infection, several aGPCRs are implicated. Bioinformatic analysis revealed an interaction between the SARS-CoV-2 spike subunit and L4 mRNA levels, which are consistently downregulated in infected cells.^651^ Additionally, D1 and G6 are upregulated in SARS-CoV-2–infected epithelial cell models. Targeted downregulation of these receptors reduces the release of free virus particles.^651^

Adhesion GPCRs also play a role in latent viral infections. G1 is upregulated in human immunodeficiency virus–positive CD4^+^ T cells^652^ and in cytomegalovirus-positive CD8^+^ T cells.^150^ Viruses also directly interact with aGPCRs, for example, binding of the mumps virus small hydrophobic protein to A3, triggering epithelial barrier dysfunction.^653^^,^^654^

Studying interactions between viral proteins and aGPCRs is key to developing antiviral therapies and understanding viral pathogenesis. Despite being an emerging field, the few interactions discovered so far suggest that much remains to be uncovered.

#### 4 Bacterial infections

B1 recognizes phosphatidylserine on the surface of apoptotic cells and bacteria, promoting their engulfment by macrophages and other phagocytes through the activation of downstream signaling pathways.^67^^,^^655^ B2 and B3 are implicated in modulating immune responses^650^^,^^656^^,^^657^ and cellular interactions during pathogen clearance.^658^, ^659^, ^660^, ^661^ Together, these receptors contribute to efficient immune surveillance and removal of infectious agents, enhancing host defense mechanisms.

#### 5 Neurodevelopmental, neurodegenerative, and neuropsychiatric disorders

B receptors play critical roles in brain development and synaptic architecture. B1 is essential for dendritic spine formation and excitatory synaptogenesis, and its absence in mice leads to social deficits, reduced brain weight, increased neuronal apoptosis, and heightened susceptibility to seizures; phenotypes relevant to autism spectrum disorder and epilepsy.^299^^,^^662^ Additionally, B1 interacts with autism-linked proteins such as neuroligin 1 and IRSp53, and autism spectrum disorder–relevant B1 variants have been reported in patients.^662^ Decreased B1 expression is observed in the substantia nigra of Parkinson disease patients as well as the substantia nigra and striatum of Parkinson disease animal models,^663^ correlating with dopaminergic neuron loss. B2 is implicated in mood regulation and emotional behavior.^664^ *B2*^*KO*^ mice exhibit antidepressant-like behavior.^663^^,^^665^ Variants in B3, including polymorphism and copy number variations, are linked to schizophrenia-related disorganization,^666^^,^^667^ addiction,^668^ epilepsy, and bipolar disorder.^669^ Mice lacking full-length B3 have reduced brain and body weights, augmented energy expenditure, and deficits in social interaction.^512^^,^^670^

Recent insights into the genetic basis of craniorachischisis, a severe neural tube defect characterized by complete failure of neural tube closure have identified several potentially pathogenic variants in components that govern PCP establishment and maintenance including C1.^221^ Functional studies revealed that although these variants did not disrupt known PPIs, they significantly impaired proper subcellular localization caused by reduced protein expression and defective plasma membrane trafficking, mechanisms consistent with phenotypes observed in analogous mouse mutants.^344^

G1 has verified roles in spermatogonia,^547^ skeletal muscle^437^^,^^547^ and brain development,^345^ CNS myelination,^362^^,^^363^ and hemostasis.^286^ Mutations in G1 cause bilateral frontoparietal polymicrogyria, a recessive cortical malformation disorder characterized by severe developmental issues, intellectual disability, and seizures.^345^^,^^347^ In adults, *G1* mRNA levels are upregulated in patients who respond to antidepressant treatment.^671^^,^^672^ Reduced *G1* expression is linked to suicide, potentially due to a *G1* splice variant affecting synaptic pruning.^220^^,^^673^

L3 is the most studied latrophilin homolog in neuropsychiatric disorders. Variants are not only linked to childhood attention deficit hyperactivity disorder^674^^,^^675^ but also connected to autism spectrum disorder^674^, ^675^, ^676^ and substance use disorder.^677^ L2 variants are associated with microcephaly with severely reduced sulcation and rhombencephalosynapsis,^678^ and with cocaine use disorder.^679^ *L1* haploinsufficiency has been linked to intellectual disability and developmental delay in a small cohort of 10 individuals^352^ and to developmental and epileptic encephalopathy.^680^

Pathogenic variants in V1 cause human USH2C, a common form of combined deaf-blindness.^681^ V1 mouse models suffer from progressive deafness and show increased susceptibility to audiogenic seizures.^682^ Recent studies have linked heterozygous variation in V1 to various forms of epilepsy in humans.^683^^,^^684^ In the CNS, defects in V1 cause alterations in myelination^310^ and dysfunctions in hippocampal astrocytes.^374^

*Critical synopsis and outlook: The extensive repertoire of aGPCRs in model organisms and humans has been complemented by a tremendous increase in our understanding of the physiological functions these receptors support. A unifying theme across many recent findings is the role of aGPCRs as mechanosensors in diverse organ systems and cellular contexts. Given their unusual molecular architecture and the autoproteolytic processing characteristic of many family members, mechanical stimuli are natural physiological inputs for aGPCR activation. Importantly, such cues appear to be essential for multiple developmental programs during and after embryogenesis. It is therefore unsurprising that germline and somatic mutations in aGPCR loci are associated with, and in some cases causal for, human developmental disorders and cancers. Future research must decipher the specific contributions of impaired aGPCR signaling to individual pathological conditions, both to understand disease mechanisms and to identify opportunities where therapeutic targeting of these receptors may benefit patients. The systematic delineation of defined signaling pathways, activating conditions, and expression patterns of individual aGPCR homologs will greatly enhance and likely accelerate this emerging translational branch of aGPCR research in the years ahead.*

## XIII Adhesion G protein–coupled receptors as drug targets

Adhesion GPCRs are emerging as promising drug targets because they integrate mechanical and chemical signals, control key physiological processes, and are involved in several pathologies. The identification of pharmacological actuators of individual aGPCR functions is an urgent need to fully exploit their immense therapeutic potential.

### A ADGRA

A3 signaling can induce adipose thermogenesis, and the proposed A3 agonistic ligand hesperetin may represent an obesity treatment.^513^ Engineered WNT ligands, designed as specific A2/RECK agonists, enable blood-brain barrier repair in neurological disorders (Fig. 6B).^409^

### B ADGRB

Herpes simplex-derived oncolytic viruses engineered to express the B1 NTF-derived vasculostatin proteolytic fragment had potent antiangiogenic and tumor-suppressive effects in brain cancer models.^685^, ^686^, ^687^, ^688^ Additionally, small molecules inhibiting the epigenetic reader MBD2 and the writer EZH2 target B1 gene epigenetic silencing and present a promising strategy to restore B1 expression and its tumor-suppressive functions.^329^^,^^689^

### C ADGRD

A role for D1 in glioblastoma progression is supported by several studies. The identification of PTK7 as an allosteric modulator of D1, along with the development of antibodies targeting the D1 NTF, provides impetus to investigate these as potential therapeutic modalities.^86^^,^^291^ D1 was also implicated as a receptor for the androgen 5*α*-DHT in muscle cells, where its action to raise cAMP levels was proposed to enhance muscle strengthening. A small molecule D1 activator, AP503, may serve as a drug lead for its beneficial actions with limited side effects.^88^

### D ADGRE

Monoclonal antibody 1B2 directed against E5 proved efficacious in a mouse model of experimental arthritis and was proposed to work by neutralizing E5 through a combination of receptor internalization and induced NTF shedding.^690^ However, later in-depth analyses of its mode of action in vivo revealed that the antibody depletes granulocytes in mice under conditions of acute inflammation via a Fc receptor-dependent mechanism.^691^ An antibody-drug conjugate against E5 demonstrated efficacious killing of human glioblastoma cells, in which E5 plays a tumorigenic role.^100^ Moreover, an afucosylated monoclonal antibody to E1 efficiently depletes eosinophilic granulocytes.^464^ E2 may regulate serum factor H-related protein FHR1-related antibody-associated vasculitis,^91^ and E2 genetic variants are associated with vibratory urticaria.^89^ Immune targeting E2^475^ or E5^593^^,^^692^ with engineered T cells expressing chimeric antigen receptors are promising novel pharmacotherapeutic tools capitalizing on the specific cellular expression patterns of aGPCRs that may prove useful in the future.

### E ADGRF

Synaptamide is an endogenous lipid that binds to the F1 GAIN domain and reduces lysophosphatidic acid–induced inflammation in mice. It elevates cAMP levels in cultured microglia and suppresses proinflammatory cytokine levels, suggesting that F1 activation by synaptamide holds therapeutic potential to ameliorate brain and peripheral tissue inflammation.^693^

### F ADGRG

G1 has varied roles in physiology and cancer progression. Small molecule antagonists, the partial agonist 3-*α*-DOG, and modulatory antibodies target the receptor. Pharmacological studies indicate that the small molecules occupy the orthosteric site in lieu of the TIA^125^, ^126^, ^127^ and exhibit selectivity for G subfamily members.^263^^,^^287^ Individual G1 antibodies inhibited glioma cell migration^694^ and promoted RhoA signaling in breast cancer cells.^695^ A G1-targeted antibody-drug conjugate proved efficacious in colorectal cancer cell models.^696^

G6 is involved in axon myelination. In vivo phenotypic screening of small molecules was performed to correct mutant G6 Zebrafish Schwann cell development, otic, and myelination defects and lead to the identification of potential agonists that are undergoing further characterization.^138^^,^^263^^,^^697^ G2/CFTR signaling is required for sperm maturation, representing a potential therapeutic target for male infertility.^314^

### G ADGRL

L2 may play a role in equilibrioception and represents a potential therapeutic target for balance disorders such as vertigo. A small molecule termed D11 was reported to block the c-mesenchymal-to-epithelial transition mediated by L2-TMC1, thereby avoiding side effects associated with traditional vestibular suppressants.^384^

*Critical synopsis and outlook: The current landscape of drug-targeting strategies for aGPCRs remains limited and fragmented, reflecting our still emerging understanding of receptor- and ortholog-specific working principles. This immaturity is further compounded by the intersection of multiple input modalities—adhesive ligands, mechanical forces, and steroid hormones—that aGPCRs are capable of integrating or distinguishing. Only once we understand in molecular detail how an individual receptor parses these diverse cues, and which structural elements enable or bias one mode of activation over another, can a focused and rational development of pharmacological agents truly begin. A major challenge lies in identifying intervention points that selectively modulate defined signaling routes without perturbing others, a task complicated by the multifunctional nature of aGPCRs. Structural elucidation of ligand-binding sites, mechanosensitive receptor states, and allosteric interfaces will therefore be indispensable. In parallel, systematic pathway deconvolution and isoform-resolved expression studies will help reveal which signaling outputs are most physiologically or pathologically relevant, thereby guiding where pharmacological precision is needed most. As these mechanistic foundations solidify, new opportunities will emerge for designing small molecules, allosteric modulators, or engineered biologicals that can intervene with unprecedented selectivity. Ultimately, the maturation of our conceptual and structural understanding of aGPCR signaling will transform the currently fragmented therapeutic landscape into a more coherent framework for targeted drug discovery.*

## XIV Phylogeny of adhesion G protein–coupled receptor(s) and model organisms

### A Phylogenetic relationships of adhesion G protein–coupled receptor(s) subfamilies and homologs

aGPCRs have been present in Metazoa for at least 750 million years^697^ and are classified into 9 families (A, B, C, D, E, F, G, L, and V) based on the phylogenetic relationships of their 7TMD^1^ (Fig. 7). This classification, however, has been challenged by recent studies, which highlight ambiguities in the hierarchical organization of GPCRs and call for a revised system based on phylogenetically supported levels.^7^^,^^15^

Notably, the secretin-like receptor class is now considered to have evolved from aGPCRs, most likely from the D subfamily.^7^^,^^15^^,^^44^^,^^45^ The evolutionary conservation of aGPCRs is evident, as at least 1 member of each family has a fish ortholog, indicating that all aGPCR families were already present by the Silurian period, approximately 419 million years ago. In vertebrates, no additional independent aGPCR families have been identified beyond the known 9, and sequences with a 1-to-1 orthology to human aGPCRs exist in at least one species from each major mammalian lineage (Monotremata, Marsupialia, and Eutheria), suggesting that the full aGPCR repertoire was already established before the rise of mammals over 178 million years ago.^15^

However, despite this conservation, aGPCRs exhibit notable gene losses, duplications, and expansions across different taxa. For example, *F3* and *E5* are absent in birds, whereas multiple paralogs of certain aGPCRs, such as *E2*, exist in species like felids, marmots, and artiodactyls.^15^^,^^698^ These variations reflect the genomic plasticity of aGPCRs, influenced by whole-genome duplications and local gene duplication events. Such dynamics have led to neofunctionalization or subfunctionalization of gene duplicates, which must be considered when studying aGPCR function in model organisms, as their roles may differ significantly from those in humans.

### B Model organisms for adhesion G protein–coupled receptor(s) research

Research with animal models is a foundation of aGPCR research and has promoted insights into many of the physiological roles that are currently attributed to them.^292^^,^^293^^,^^699^, ^700^, ^701^ In the following section, the main model species currently in use and their individual utility for questions pertaining to aGPCRs are introduced.

#### 1 Choanoflagellates

As the closest living relatives of animals, choanoflagellates provide a window into the early evolution of GPCRs, and aGPCRs in particular.^702^^,^^703^ Systematic analyses of GPCR repertoires in 23 choanoflagellate species, including *Salpingoeca rosetta*, have revealed that aGPCRs constitute the largest GPCR family in most choanoflagellates, with up to 41 aGPCRs predicted in some species.^44^^,^^704^, ^705^, ^706^ Interestingly, the abundance and diversity of protein domains in the NTFs of choanoflagellate aGPCRs rival those found in metazoans, suggesting that the number of aGPCRs likely increased and their protein domain architectures diversified in the stem lineage leading to both metazoans and choanoflagellates.

Eighteen of the 19 choanoflagellate aGPCR subfamilies detected appear to have diversified independently from metazoan aGPCRs, with the exception of the V subfamily.^44^ Nonetheless, choanoflagellate and metazoan aGPCRs share key structural features in the form of the HormR/GAIN/7TMD layout along with additional extracellular domains.^706^ Thus, the HormR/GAIN/7TMD and diversity of other NTF domains evolved before the divergence of choanoflagellates and metazoans and were, therefore, foundational to the subsequent evolution of metazoan aGPCRs.

Future efforts to reconstruct the premetazoan functions of aGPCRs will benefit from the study of aGPCR functions and regulation in phylogenetically relevant organisms, including choanoflagellates and diverse early branching animals.

#### 2 Fruit fly (Drosophila melanogaster)

The aGPCR family in *D. melanogaster* contains 5 homologs allocated to subfamilies ADGRA (Remoulade), ADGRC (Flamingo/Starry night), ADGRL/A (Cirl), and 1 group equally evolutionarily related to all known aGPCR subfamilies termed ADGRX (Mayo, Ketchup).^7^

*Remoulade/CG15744 (remo*). The predicted ENT of Remo is structurally homologous to that of vertebrate A2 (Fig. 1A). Notably, its irregular GPS (H^−2^R^−1^T^+1^) suggests that it is not self-cleavable.^153^ Structure predictions of the *remo* gene product indicate coiled-coil elements in its ICT. Unlike A2, Remo lacks a C-terminal PBM. Recent observations have connected Remo signal transduction to Rac1, a member of the Rho family of small GTPases, and axon growth guidance in the CNS of the fly.^707^ It is unclear whether Remo can also work akin to A2 as a WNT coreceptor in complex with RECK, Frizzled, and Lrp5/6 (Fig. 6B).

*Flamingo/Starry night/CG11895 (Fmi* or *Stan*) shares the same basic domain structure of ADGRC homologs, consisting of cadherin repeats, an EGF-LamG region, HormR, GAIN domain and 7TMDs, and a long intracellular domain (Fig. 1A).^259^^,^^708^ It is most extensively characterized for its function in the “core” PCP pathway, controlling the orientation of epithelial structures, such as hairs, bristles, and the ommatidial units of the compound eye.^259^^,^^708^^,^^709^ Here, Fmi interacts homophilically at cell-cell contacts, binding asymmetrically with the Frizzled 7TMD receptor in 1 cell and the Stbm/Vang 4TM protein in the opposing cell, thus apparently acting as both ligand and receptor for itself.^259^^,^^332^^,^^710^, ^711^, ^712^, ^713^ Flamingo also functions extensively in *Drosophila* peripheral and CNS development. This includes dendritic patterning,^714^^,^^715^ photoreceptor target selection in the brain,^381^^,^^716^ and mushroom body development.^717^, ^718^, ^719^ Notably, both cell autonomous functions involving unidentified ligands^715^^,^^720^ and homophilic functions involving Fmi-Fmi binding^342^^,^^720^ have been reported. Moreover, although these functions appear to be independent of planar polarity pathway function,^381^^,^^715^ in several contexts, planar polarity proteins also appear to act together with Fmi in the nervous system.^718^^,^^719^^,^^721^^,^^722^

*Cirl/CG8639*. Similar to other ADGRL homologs, Cirl contains an extracellular RBL, HormR, and GAIN domains but lacks an OLF domain (Fig. 1A).^379^ Deletion of Cirl results in a broad reduction in the mechanosensitivity of larvae, including tactile sensitivity to gentle touch, auditory, proprioceptive, and nocifensive stimuli.^194^^,^^195^^,^^211^^,^^379^ This is explained by the expression of Cirl in dendrites and cilia of chordotonal neurons,^195^^,^^723^ which serve as the main mechanosensory nerve cells in insects and nociceptive nerve cells.^194^ In addition, Cirl is required for setting the number of neurons generated during central brain neurogenesis, where the aGPCR functions together with the TLR Tollo/Toll-8.^153^ For this function as a metabotropic mechanosensory, Cirl self-cleavage is necessary,^153^ whereas its mechanosensory role in the periphery does not require GAIN domain-mediated receptor autoproteolysis.^194^^,^^195^ The Cirl-Tollo interaction is also required for the planar cell polarization of contractile cell-cell contacts in embryonic ectoderm.^154^ In the visual system, Cirl appears to affect activity-dependent synaptic assembly.^380^

*Mayo/CG11318* and *Ketchup/CG15556 (ktch*). Mayo and Ketchup are minimalist aGPCRs whose structure predictions only indicate the presence of GAIN and 7TM domains in each.^7^ Both proteins contain a canonical GPS and are self-proteolysed.^153^^,^^339^ Mayo is expressed in the epithelia of the midgut and anal plate of third instar larvae.^724^ Genetic removal of *mayo* impacts enterocyte proliferation in the larval midgut, leading to a non–cell autonomous increase in potassium concentration in the hemolymph, which results in tachycardia.^339^ Ketchup is expressed in the proventriculus of the gastrointestinal canal and Malpighian tubules, which function as the kidney equivalent in insects.^724^ Ketchup’s function in these organs, which regulate ion and water homeostasis, is unknown at the present time.

#### 3 Roundworm (Caenorhabditis elegans)

In the nematode *C. elegans*, the following 3 aGPCRs exist: the L homologs LAT-1 and LAT-2, and the C homolog FMI-1.

Like its mammalian homologs (Fig. 1A), LAT-1 plays a role in the neuronal system of *C. elegans*. Here, it is essential for neuronal morphogenesis, affecting sensory structures such as the male sensory rays and head sensilla. Consistent with this, the absence of LAT-1 leads to defective male copulation behavior.^341^ Beyond its neuronal role, LAT-1 regulates anterior-posterior division plane orientations in the early embryo via G*α*_s_ signaling^295^^,^^534^ and contributes to fertility by modulating sperm guidance, ovulation, and germ cell apoptosis.^219^^,^^294^ Furthermore, LAT-1 modulates Notch signaling in the stem cell niche of the *C. elegans* gonad via direct interaction with the DSL ligand, thereby regulating germ cell proliferation to ensure the correct number of germ cells.^156^

LAT-2, in contrast, remains less understood. Expression analyses revealed its presence in the pharyngeal primordium during embryogenesis and later in the pharynx and excretory system, suggesting roles in feeding and waste regulation.^534^

Unlike in other species, the role of FMI-1 in PCP has not been described in *C. elegans* yet. The receptor is involved in neuronal circuit formation by controlling axon growth and pioneer-dependent axon navigation independently of PCP pathways.^343^^,^^725^^,^^726^ It also regulates dendrite self-avoidance by antagonizing the PCP component VANG-1/van Gogh.^727^ Additionally, FMI-1 has been linked to body size regulation and ECM composition, together with other PCP components.^728^

#### 4 Zebrafish (Danio rerio)

The zebrafish aGPCR repertoire contains close to 60 members that represent homologs of 24 of the mammalian 33 aGPCR (Fig. 1A).^493^ Missing homologs are restricted to subfamilies E, F, and G, which also exhibit zebrafish-specific expansions. These expansions, combined with the genome-wide duplication event that occurred within the teleost lineage, explain the larger number of zebrafish aGPCRs. Many true orthologs of human aGPCRs exist in zebrafish, making this model organism powerful to study their function.

L2 has been found to mediate shear stress mechanotransduction^406^ and control vascular permeability in zebrafish endothelial cells.^408^ CRISPR-Cas9 mutants and morphants of L3.1 (*lphn3*), the ortholog of L3, have been used to investigate its role in attention-deficit/hyperactivity and other externalizing disorders, revealing altered dopaminergic neuron distributions, hyperactive motor phenotypes, potential new therapeutic targets, and therapeutic and metabolic effects of existing and emerging drugs.^729^, ^730^, ^731^, ^732^, ^733^, ^734^, ^735^, ^736^, ^737^

The zebrafish paralogs of C1 (*adgrc1a* and *b*) regulate convergence and extension (CE) and epiboly movements during gastrulation,^738^^,^^739^ as well as tissue homeostasis and aging phenotypes in adults.^699^ Besides its role in CE,^739^ C2 controls facial motor neuron migration in the developing hindbrain.^740^ C3 is required for the development of GABA and acoustic startle circuits in the inner retina^741^ and hindbrain.^742^

Zebrafish A2 has been investigated for its role in promoting WNT/*β*-catenin signaling during brain angiogenesis and dorsal root ganglia formation, contributing to the identification of its coreceptor RECK,^53^ the delineation of its mechanism in WNT ligand-specific signaling,^54^^,^^56^ the identification of highly specific pathway agonists,^356^ and its downstream angiogenic effector (Fig. 6B).^343^ The closely related A3 was found to regulate PCP during zebrafish VE movements by binding Dishevelled,^273^ an interaction shared with zebrafish A2.^54^

The role of G1 in oligodendrocyte development and peripheral myelination was revealed by the analysis of zebrafish G1 mutants.^123^^,^^362^ Microglial-derived transglutaminase 2 was identified as a relevant ligand for A1 during CNS myelination, a finding supported by somatic gene disruptions in zebrafish.^365^ Morpholino knockdown studies further implicated this receptor in the formation of hematopoietic stem cells,^743^ a process that could be compensated by the ectopic expression of G3.^472^

Forward genetic screens in zebrafish revealed the essential role of G6 in PNS myelination.^367^ Additional roles of this receptor in zebrafish include spine ossification,^454^ cardiac trabeculation,^319^ and inner ear morphogenesis.^744^ The zebrafish model also contributed to elucidating the context-dependent mechanisms of G6 signaling, defining its ligands, and identifying potential small-molecule agonists and posttranscriptional regulators.^32^^,^^132^^,^^134^^,^^135^^,^^137^^,^^138^^,^^263^^,^^319^^,^^745^

Zebrafish V1 models have been generated for the analysis of V1 functions and disease mechanisms associated with mutations in V1.^746^, ^747^, ^748^ The *adgrv1*^*rmc22*^ zebrafish is the first *V1* mutant model for Usher syndrome type 2C that displays an early retinal dysfunction, which can be used as outcome measures in the evaluation of therapeutic strategies.^747^

#### 5 Chick (Gallus gallus)

In the chick embryo (avian) model for developmental biology studies, the ability to fenestrate the eggshell provides ease of access to the embryo and enables temporal manipulation of live embryo development. Thus, changes can be made to tissue position through grafting,^749^ gene expression,^749^^,^^750^ and cell signaling pathways.^751^ Overall, the chick model has contributed knowledge of C1 function in key gastrulation events^752^ as well as in neural tube closure mechanisms.^272^ The rich vascular network of the chick embryo chorioallantoic membrane also provides a unique assay system,^753^ offering efficient screening for antiangiogenic drugs^754^^,^^755^ as well as for studying cancer biology.^400^^,^^756^^,^^757^ A sterile environment can be easily maintained following tumor cell inoculation in vivo, and since the avian immune system is not mature until day 15 postgestation, immune rejection of xenografted cells is prevented. Indeed, a chorioallantoic membrane assay-based drug screen recently identified Dub as an angiogenesis inhibitor for breast cancer, which acts via the regulation of B1.^758^

#### 6 Mouse (Mus musculus) and rat (Rattus norvegicus)

Mice and rats offer several advantages for studying the complex roles of aGPCRs, including their genetic similarity to humans, the wide availability of genetic models, and their relevance to humans. The average protein identity between human and mouse aGPCRs is approximately 81%, with a minimum of 60% (G4) and a maximum of 98.5% (L1).

Due to the large exon-intron structure and numerous splice variants of aGPCRs,^204^ rodent KO models require careful design. Global KO of several aGPCRs, such as *A2*, *F5*, and *G6*, leads to severe phenotypes including embryonic lethality,^58^^,^^394^ surfactant accumulation,^112^^,^^266^^,^^267^ and perinatal death.^368^^,^^426^ In such cases, tissue-specific KO models can uncover more subtle receptor functions. Indeed, except for an *L3*^*KO*^ rat model,^759^^,^^760^ most *aGPCR*^*KOs*^ were generated in mice and use Cre-loxP systems for tissue-specific or temporally controlled receptor deletions (Table 2).^761^, ^762^, ^763^, ^764^, ^765^, ^766^, ^767^, ^768^, ^769^, ^770^, ^771^, ^772^, ^773^, ^774^, ^775^, ^776^, ^777^, ^778^, ^779^, ^780^, ^781^, ^782^, ^783^, ^784^, ^785^

**Table 2:** Currently available mouse models with associated publications from the Mouse Genome Informatics, International Mouse Strain Resource, and The Jackson Laboratory databases

Receptor	Strain	Repository/Depositor	Description	References
*ADGRA1*	*Adgra1*^*em1(IMPC)J*^	The Jackson Laboratory	Null/KO	^761^
	*Adgra1*^*tm1b(EUCOMM)Hmgu*^	Helmholtz Zentrum Muenchen GmbH	Null/KO	^762^
*ADGRA2*	*Adgra2*^*tm1.1Bstc*^	JAX 016881	Targeted (conditional ready); Exon 1 flanked by loxP sites for Cre-mediated excision	^395^
	*Gpr124 null*	Velocigene	Null/KO; Exons 3-13 targeted	^394^
*ADGRA3*	*Adgra3*^*tm1.1(HBEGF,-cre/ERT2)Pac*^	JAX 068344	Tamoxifen-inducible; Exon 1 targeted for null/KO	^595^
	*Adgra3*^*tm1Lex*^	Lexicon Pharmaceuticals	Null/KO	^762^
*ADGRB1*	*Adgrb1*^*tm2a(EUCOMM)Wtsi*^	Wellcome Trust Sanger Institute	Targeted (conditional ready); exons targeted for Cre-mediated excision	^763^
	*Adgrb1*^*t-/-*^	Erwin G. Van Meir	Constitutive Null/knockout of full-length isoform; exon 2 targeted; short Bai1 isoforms remains expressed from the alternative promoter	^328^
	*Bai1*^*−/−*^	University of Virginia	Gene-trap mutation between exons 2 and 3	^529^
*ADGRB2*	*Adgrb2*^*tm1b(KOMP)Mbp*^	UC Davis	Null/KO	^764^
*ADGRB3*	*Adgrb3*^*em1(IMPC)Bay*^	Baylor College of Medicine	Null/KO	^761^
	*Bai3*^*Flox*^	Michisuke Yuzaki	Targeted (conditional ready); exons targeted for Cre-mediated excision	^77^
	*Adgrb3*^*Δ7/Δ7*^	Erwin G. Van Meir	Constitutive null/knockout of full-length isoform; exon 10 targeted; short Bai3 isoforms remain expressed from the alternative promoter	^670^
	*Bai3*^*-/-*^	Sushant Bhatnagar	Constitutive null/knockout of full-length and short isoforms; exons 2 and 18 targeted	^512^
	*Adgrb3*^*Tn(sb-lacZ,GFP)PV449Jtak*^	Junji Takeda	Transposon insertion	^765^
*ADGRC1*	*Celsr1*^*ctb*^	JAX 016111	Spontaneous null/knockout; single G deletion results in a frameshift mutation	^766^
	*Celsr1*^*Crsh*^		Single-point mutation, D1040G	^344^
	*Celsr1*^*Scy*^		Single-point mutation, N1110K	^767^
	*Celsr1*^*em1(IMPC)Mbp*^	IMPC UC Davis	Null/KO	^761^
	*Celsr1*^*KO*^		Null/KO; exons 26–29 targeted	^768^
*ADGRC2*	*Celsr2*^*tm1Dgen*^	JAX 005779	Null/KO; exon 23 targeted for insertion of bacterial lacZ	^769^
	*Celsr2*^*KO*^		Null/KO; exons 16–28 targeted	^770^
*ADGRC3*	*Celsr3*^*KO*^		Null/KO; exons 19–27 targeted	^771^
*ADGRD1*	*Adgrd1*^*tm1a(EUCOMM)Wtsi*^	Wellcome Trust Sanger Institute	Targeted (conditional ready); exons targeted for Cre-mediated excision	^763^^,^^772^
	*Adgrd1*^*tm1b(EUCOMM)Wtsi*^	Wellcome Trust Sanger Institute	Null/knockout	^772^
*ADGRD2*	*No current mouse models - ADGRD2 is a pseudogene in mice and is therefore non-functional*			
*ADGRE1*	*Adgre1*^*tm1(cre)Kpf*^	Klaus Pfeffer	Targeted; endogenous coding sequence replaced with Cre and Neo	^773^
	*Adgre1*^*tm1.1Mrl*^	Merck Research Laboratory	Targeted (conditional ready); Exon 16-17 flanked by loxP sites for Cre-mediated excision	^774^
*ADGRE2*	*No mouse orthologs*			
*ADGRE3*	*No mouse orthologs*			
*ADGRE4*	*Adgre4*^*tm1b(EUCOMM)Hmgu*^	Helmholtz Zentrum Muenchen GmbH	Null/knockout; target exon flanked by loxP sites for Cre-mediated excision, reporter tagged	^761^
*ADGRE5*	*Adgre5*^*tm1Dgen*^	JAX 005788, Deltagen	Null/knockout; exons 2-5 targeted	^774^
	*Adgre5*^*tm1Kake*^	Kathleen Kelly	Null/knockout; exons 2-12 replaced with PGKneo cassette	^470^
	*Adgre5*^*em2Cys*^	Jason G. Cyster	Null/knockout; exons 2-3 targeted using CRISPR-Cas9 for intragenic deletion	^94^
*ADGRF1*	*Adgrf1*^*tm1Tcam*^	Takeda Cambridge	Null/knockout; exon 12 targeted	^423^
	*Adgrf1*^*tm1Smoc*^	Shanghai Model Organisms Center	Null/knockout; exons 11-13 targeted	^519^
	*Adgrf1*^*tm1a(KOMP)Wtsi*^	Wellcome Trust Sanger Institute	Targeted (conditional ready); exons targeted for Cre-mediated excision	^763^
*ADGRF2*	*Adgrf2*^*tm1Tcam*^	Andreas P Russ/Takeda Pharmaceuticals	Null/knockout; exon 7 targeted	^423^
	*Adgrf2*^*em1Iwto*^	Tsutomu Iwamoto	Null/knockout; exon 3 targeted	^775^
*ADGRF3*	*Adgrf3*^*em1(IMPC)J*^	JAX 042379	Null/knockout; exon 8 targeted	^761^
	*Adgrf3*^*tm1Lex*^	Lexicon Pharmaceuticals	Null/knockout	^762^
	*Adgrf3*^*em1Kzt*^	Keizo Tokuhiro	Null/KO; exons 7-10 targeted	^776^
*ADGRF4*	*Adgrf4*^*tm1Tcam*^	Takeda Cambridge	Null/knockout; exon 6 targeted	^423^
	*Adgrf4*^*tm1a(KOMP)Wtsi*^	Wellcome Trust Sanger Institute	Targeted (conditional ready); exons targeted for Cre-mediated excision	^763^
	*Adgrf4*^*tm2a(EUCOMM)Wtsi*^	Wellcome Trust Sanger Institute	Targeted (conditional ready); exons targeted for Cre-mediated excision	^763^
*ADGRF5*	*Adgrf5*^*tm1.1Bstc*^	JAX 022505	Targeted (conditional ready); exon 2 targeted	^266^
	*Adgrf5*^*tm1Shiro*^	Shigehisa Hirose	Null/knockout; exon 2 targeted	^112^
	*Adgrf5*^*tm1.1Jpbs*^	James P Bridges	Targeted (conditional ready); exon 17 targeted for Flp-mediated recombination	^267^
	*Adgrf5*^*tm1.2Jpbs*^	James P Bridges	Null/knockout; exon 17 targeted	^267^
	*Adgrf5*^*tm1a(KOMP)Wtsi*^	Wellcome Trust Sanger Institute	Targeted (conditional ready)	^763^
	*Adgrf5*^*tm1b(KOMP)Wtsi*^	Wellcome Trust Sanger Institute	Cre-excision of the tm1a allele	^772^
*ADGRG1*	*Adgrg1*^*tm1Lex*^	Lexicon Pharmaceuticals	Null/knockout; exons 2-3 targeted	^307^
	*Tg(Adgrg1-EGFP)HC35Gsat*	Rockefeller University	Transgenic; EGFP reporter gene inserted at the initiating codon of the first coding exon	^121^
*ADGRG2*	*HE6*^*KO*^		Null/knockout; exons 22-25 targeted	^551^
*ADGRG3*	*Adgrg3*^*tm1Fwa*^	Frederick W Alt	Null/knockout; exons 2-8 targeted	^777^
	*Adgrg3*^*tm1Smoc*^	Shanghai Model Organisms Center	Null/knockout; exons 1-2 targeted	^642^
*ADGRG4*	*Adgrg4*^*em1(IMPC)J*^	JAX 051244	Null/knockout; exon 3 targeted	^761^
*ADGRG5*	*Adgrg5*^*tm1Lex*^	Lexicon Pharmaceuticals	Null/knockout	^762^
	*Adgrg5*^*tm1a(EUCOMM)Wtsi*^	Wellcome Trust Sanger Institute	Targeted (conditional ready); exons targeted for Cre-mediated excision	^763^
	*Adgrg5*^*tm1b(EUCOMM)Wtsi*^	Wellcome Trust Sanger Institute	Null/knockout	^761^
*ADGRG6*	*Adgrg6*^*tm1Apr*^	Andreas P Russ/Takeda Pharmaceuticals	Null/knockout; exon 18 targeted	^426^
	*Adgrg6*^*tm1Taki*^	Tetsu Akiyama	Null/KO; exon 2 targeted	^319^
	*Adgrg6*^*tm1Smoc*^	Shanghai Model Organisms Center	Targeted (conditional ready); exon 2 targeted	^778^
	*Adgrg6*^*em1Jlp*^	Jose Luis de la Pompa	Intragenic deletion of exons 3 and 4 producing an N-terminal fragment lacking the CUB and PTX domains	^434^
	*Adgrg6*^*em2Jlp*^	Jose Luis de la Pompa	Null/knockout; exon 7 targeted	^434^
	*Adgrg6*^*tm1a(EUCOMM)Hmgu*^	Helmholtz Zentrum Muenchen GmbH	Targeted (conditional ready)	^425^
	*Gpr126*^*-/-*^	Lexicon Pharmaceuticals	Conditional ready	^310^
*ADGRG7*	*Adgrg7*^*tm1Wfro*^	Wei-Fang Rong	Null/knockout; exons 10-12 targeted	^530^
	*Adgrg7*^*tm1b(EUCOMM)Hmgu*^	Helmholtz Zentrum Muenchen GmbH	Null/knockout	^761^
*ADGRL1*	*Adgrl1*^*tm1Sud*^	JAX 006393	Null/knockout; exons 1-2 targeted	^779^
	*Adgrl1*^*tm2.1Sud*^	JAX 035181	Null/knockout; Myc-tagged mouse *ADGRL1* with floxed exon 2 for Cre-mediated excision	^223^
	*Adgrl1*^*tm2c(EUCOMM)Hmgu*^	JAX 035185	Targeted (conditional ready)	^223^
	*Adgrl1*^*-*^		Null/knockout; exons 1-3 targeted	^352^
*ADGRL2*	*Lphn2*^*tm1Dgen*^	HAR/EMMA	Null/knockout	^1^^,^^678^
	*Adgrl2*^*tm1Sud*^	JAX 023401	Targeted (conditional ready) contains Frt sites and a part of loxP sites for Cre-mediated excision or expression of an alternative transcript fused to mVenus	^350^
*ADGRL3*	*Adgrl3*^*tm1Sud*^	JAX 026684	Conditional; exon 6 flanked by loxP sites for Cre-mediated excision	^351^
	*Adgrl3*^*Gt(S17-5H1)Sor*^	Texas A&M Institute for Genomic Medicine	Null/knockout	^780^
	*Adgrl3*^*tm1(KOMP)Vlcg*^	Velocigene	Null/knockout	^218^
	*Adgrl3*^*tm1.1(KOMP)Vlcg*^	Velocigene	Null/knockout	^772^
*ADGRL4*	*Adgrl4*^*tm1Dgen*^	DeltaGen, European Mouse Mutant Archive, HAR	Null/knockout	^436^
	*Adgrl4*^*tm1Lex*^	Lexicon Pharmaceuticals	Null/knockout; exons 4-6 targeted	^762^
	*Adgrl4*^*em1(IMPC)Mbp*^	IMPC UC Davis	Null/knockout	^761^
*ADGRV1*	*Adgrv1*^*tm1Pwh*^	Perrin C White, JAX 009379	Null/knockout; exon 82 targeted	^682^
	*Adgrv1*^*tm1Msat*^	Makoto Sato	Null/knockout; exons 2-4 targeted	^781^
	*Adgrv1*^*tm2Msat*^	Makoto Sato	Null/knockout; exons 2-4 targeted, insertion of YFP	^782^
	*Adgrv1*^*tm1.1(KOMP)Vlcg*^	Velocigene	Null/knockout	^772^
	*Adgrv1*^*m1*^		Spontaneous Null/knockout; deletions in Exon 31 causes frameshift and premature stop codon	^783^
	*Adgrv1*^*frings*^		Spontaneous intragenic deletion; single nucleotide deletion that results in a nonsense mutation in Exon 27	^784^^,^^785^

Some studies have used knock-in models to introduce tags (eg, GFP, mCherry, HA, and Myc) to facilitate receptor visualization in live cells or tissues.^210^^,^^223^^,^^350^ This is especially valuable, given the challenges in developing antibodies for aGPCRs.

In addition to null models, disease mouse models help investigate how mutations disrupt protein function and cellular processes. For example, the human *Y6244fsX1* mutation in *V1*, a key component of the ankle-link complex essential for cochlear hair cell development, was modeled in *V1 Y6236fsX1* mice. These mice recapitulated Usher syndrome type 2, demonstrating the relevance of mutant mice for studying disease mechanisms.^681^ Notably, no humanized aGPCR rodent models exist, suggesting a promising area for future research.

*Critical synopsis and outlook: Evolutionary analyses of aGPCRs have both supported and propelled research into their underlying working principles. The recent discovery of a broad aGPCR repertoire in unicellular species at the brink of multicellularity underscores the importance of their molecular design in mediating cell-cell communication, likely by enabling the exchange of adhesive and mechanical cues within cell communities. Looking back into the evolutionary history of aGPCRs will continue to guide the recognition—and experimental interrogation, in both invertebrate and vertebrate models—of functional features that have remained conserved for at least 600 million years. Studies of the coevolution of extracellular aGPCR-ligand-binding domains and their cognate ligands have not yet been widely initiated, even though this line of inquiry may reveal the determinants of receptor-ligand specificity and thereby inform strategies for selectively modulating aGPCR function pharmacologically. Moreover, deciphering the evolutionary logic by which extracellular domains and ligands coadapted to enable the diverse mechanochemical signaling modes characteristic of aGPCRs may offer important insights into how receptor architectures became tailored to the environmental and biomechanical properties of their cellular expression sites across different organisms, organs, and tissues.*

## XV Experimental technologies for adhesion G protein–coupled receptor(s) interrogation

### A Molecular dynamics studies

Studies of how aGPCR proteins behave and move using molecular dynamic (MD) simulations have mainly focused on the GAIN domain (Fig. 4) and have uncovered 2 dynamic regions near the GPS, termed flap 1 and flap 2 (Fig. 8).^30^ Expanding on this perspective, MD simulations supported investigations into the GPS cleavage mechanism, validating the presence of a T-shaped *π-π* interaction of the catalytic triad histidine as a key determinant of GPS cleavage competence in the GAIN domain.^52^ In a biophysical study on CTF-NTF dissociation at the GAIN domain using single-molecule atomic force spectroscopy, MD was used to investigate GAIN mechanical stability and force propagation determinants of G1 mechanosensing.^786^^,^^787^

With the characterization of 7TMD structures of several aGPCRs, future MD investigations are bound to uncover dynamics of *Stachel*-binding and *Stachel*-dependent signal transduction (Fig. 6), as well as using first GAIN/7TMD complexes^27^^,^^50^ to investigate the dynamic continuum of *Stachel* release off the GAIN domain and transition into the 7TMD binding pocket (Fig. 4).

### B Homology modeling

Homology modeling serves as a critical computational tool for resolving aGPCR structures by leveraging conserved TMH frameworks from known homologous templates to predict the 3-dimensional conformations of target receptors.^788^ Its core workflow encompasses template selection, backbone mapping of transmembrane regions, side-chain optimization, and loop modeling, supplemented by MD simulations to refine dynamic conformations, such as the outward movement of the TMH6 in activated states.^789^^,^^790^

However, traditional homology modeling faces limitations in accuracy due to challenges, including template scarcity for aGPCRs and deviations in side-chain orientations within ligand-binding pockets. Deep learning–based protein structure prediction has emerged as a powerful technique for resolving the conformations of membrane proteins, including GPCRs. Recent advances in deep learning models, such as AlphaFold and RoseTTAFold, have surpassed the accuracy limitations of traditional homology modeling, achieving near-experimental resolution for full-sequence structure prediction.^791^^,^^792^ These approaches primarily rely on multiple sequence alignments and coevolutionary analysis, utilizing neural networks to learn spatial constraints and contact maps between residues, thereby inferring protein folding patterns.^10^ For highly flexible membrane proteins like aGPCRs, deep learning enables precise prediction of TMH topology. However, challenges remain in loop modeling, conformational dynamics, and fine-grained predictions of ligand-binding pockets.^793^

### C Acute receptor activation

Acute activation strategies have enabled investigation of the intracellular signaling activity of aGPCRs in vitro and are emerging as tools to study aGPCR functions in vivo (Fig. 8). Two main approaches, one relying on addition of ligands (Table 1) and the other on controlled exposure of the *Stachel*, have been explored.

Addition of soluble small molecules^88^^,^^108^^,^^125^^,^^126^^,^^136^^,^^137^^,^^230^ or synthetic peptides derived from the *Stachel* sequence,^32^^,^^33^^,^^77^^,^^174^^,^^176^^,^^216^^,^^281^^,^^287^^,^^312^^,^^701^ combined with measurements of G protein activation, GTP turnover, or second messenger regulation, has been used to interrogate aGPCR signaling in a manner analogous to classical GPCR agonists (Fig. 6A). However, ligand specificity and solubility remain a challenge in these approaches.

For 4 aGPCRs (G1, G6, D1, and L3), naturally occurring adhesive ligands (derived from the ECM or membrane-anchored) have also been shown to induce second messenger regulation upon acute presentation as purified proteins in solution^117^^,^^132^^,^^133^ or when presented on coated substrates or through cell mixing.^86^^,^^87^^,^^322^ Antibodies binding the NTF of G6 and D1 have been shown to induce cAMP production.^291^^,^^794^ In future assay development, testing the impact of adhesive ligands in a more physiological setting, for example, in a 3D matrix or in coculture while simultaneously applying an acute stimulus such as mechanical stress, will be informative. This can be supported by acute membrane anchoring of secreted ligand forms by exploiting genetic code expansion technology combined with biorthogonal integration of unnatural AAs within the ligand. When expressed in coculture with cognate receptor-expressing cells, such acute membrane ligand fixation can aid in investigating adhesion-dependent aGPCR activation.^795^

Signaling profiling, which systematically screens the 4 main G protein pathways and directly measures G protein coupling, is now possible through strategies that acutely expose the *Stachel*, thereby circumventing ligand solubility issues. One of the first approaches used urea to dissociate the NTF and expose the *Stachel*.^33^

In recent years, the strategy of engineering a protease site N-terminal to the *Stachel* to acutely trigger its exposure upon protease addition^188^^,^^226^^,^^284^^,^^290^^,^^300^^,^^796^, ^797^, ^798^ has paved the way for using live cell BRET readouts to monitor direct G protein activation and downstream effector interactions (Fig. 6A, B).

### D Force assays

A wide range of aGPCRs are involved in mechanotransduction, that is, the conversion of mechanical forces into biochemically and physiologically actionable information.^292^^,^^383^^,^^471^^,^^799^^,^^800^ Experimental approaches to define forces that are transmitted between aGPCRs and their adhesive ligands (Table 1) during mechanical stimulation, which affect individual aGPCR during signal transduction and influence cellular responses to aGPCR activation, are a rapidly emerging area in aGPCR research (Fig. 8). These efforts are essential for determining the specific role of a given aGPCR within the distinct phases of mechanotransduction:mechanotransmission, the transfer of forces to and between molecules and cells; mechanosensation, the detection of such forces by conformational changes within membrane proteins; and mechanoresponse,^801^ the cellular signaling reaction triggered by this molecular level of force detection. However, for many aGPCRs and their observed functions, these roles have yet to be clearly defined.

Techniques that enable direct application of force to receptors while simultaneously measuring intracellular signaling will pave the way for directly linking GAIN domain force load to GPCR signaling output. One such method combines optical tweezers and confocal microscopy, using a highly focused laser beam to manipulate (hold, pull, or push) living cells mechanically^802^^,^^803^ while simultaneously monitoring fluorescent signals. This approach leverages reporters that detect G protein recruitment, cAMP production,^804^ calcium mobilization,^805^ or Rho kinase activity^806^ downstream of aGPCR activation (Figs. 6 and 8).

Magnetic tweezers apply physiologically relevant force-loading rates (∼pN/s) to measure conformational changes and GAIN domain dissociation of aGPCRs (Figs. 4, 6, and 8).^807^^,^^808^ Recent studies show that the GAIN B subdomain of self-cleaved receptors such as G1, L1, and L3 undergoes partial unfolding at low forces before dissociating at 10–20 pN. Similar partial unfolding was also observed in noncleavable B3. These findings reveal conserved mechanical responses of the GAIN B subdomain across aGPCR subfamilies.

Single-molecule atomic force microscopy (AFM) is a valuable tool for characterizing the mechanical properties of individual protein domains and PPIs^809^ and has been used extensively for studying GPCR (Fig. 8).^810^ However, its application to aGPCRs is more limited, with only a single report documenting GAIN-*Stachel* dissociation forces for isolated GAIN domains lacking the 7TMD in a range of ∼ 95–160 pN at loading rates of 2000–20,000 pN/s.^786^

The activation of G6 through its endogenous ligands, laminin 211 and collagen IV, in combination with pulling or pushing forces were studied using AFM with a coated cantilever.^794^ A fluorescent cAMP sensor enabled measurement of this second messenger at the single-cell level, revealing distinct activation modes for these ligands.

A high-throughput mechanical stimulation assay utilizing a magnetic tweezer system, integrated with a GPCR biosensor platform, was developed to examine the mechanosensitivity of selected aGPCRs.^384^ In these assays, HEK293 cells expressing N-terminal Flag-tagged aGPCR and G protein BRET probes were incubated with paramagnetic beads, which were coated with anti-Flag M2 antibody. Forces were applied to the receptor-bound magnetic beads, enabling a real-time detection of force-induced G protein activation via a G protein dissociation BRET assay (Fig. 8). Using this method, 5 aGPCRs—D1, G6, L2, L3. and V1—were found to trigger G*α*_s_ or G*α*_i_ signaling in response to force stimulation. The platform’s versatility extends to endogenous aGPCR studies by leveraging the magnetic beads coated with antibodies specifically recognizing the extracellular regions of the target receptors.^384^ The force-induced changes in secondary messengers, such as cAMP and Ca^2+^, or other cellular signals, could also be quantified.

Vibration and shaking of heterologous cell cultures expressing aGPCRs G6,^134^ D1,^172^ and G5^216^ have also been used to study the impact of broad mechanical forces on receptor activity (Fig. 8).

Physiological force-dependency of cellular aGPCR functions were first observed in flies^325^ and mice.^437^ Increased G1 expression was shown to modulate skeletal muscle hypertrophy as a target in a transcriptional cascade active during exercise. Similarly, anabolic effects of G1 on skeletal muscle mass in vivo were achieved through wheel-running and muscle overload by stretching.^437^ Through direct sensory neuronal stimulation via Piezo-actuated glass probes operated between 100 and 1500 Hz showed Cirl-dependent suppression of cAMP levels using a transgenic EPAC sensor.^195^^,^^811^ Through electrophysiological recordings of proprioceptive neurons, Cirl-dependent maintenance of neuronal current amplitudes and frequency were demonstrated, which underlie mechanical stimulus-instructed organ functions and behaviors such as hearing, tactile perception, animal movement, and nociception.^194^^,^^195^^,^^211^^,^^379^ Additional technical efforts provided transgenic sensors to investigate physiological ligand- and force-dependent receptor dissociation as observed in proprioceptive neurons during joint motion or central brain neurogenesis,^153^^,^^723^ collectively placing Cirl within the processes of mechano-transmission and -sensing.

Recently, similar physiological approaches demonstrated the mechanosensing contribution of L2^384^ and D1^385^ to equilibrioception in mice. A fluid jet system was used to directly apply mechanical stimuli to the cell surface of hair cells.^812^ When integrated with BRET-based biosensors (Fig. 8), this system confirmed the mechanosensitivity of selected aGPCRs identified by a magnetic tweezer assay. Furthermore, the fluid jet system has been utilized to explore the physiological roles of aGPCR in the inner ear hair cells. Specifically, activation of L2 by the fluid jet stimulation increases intracellular Ca^2+^ levels and promotes glutamate secretion in mouse vestibular hair cells, a response that is severely impaired in L2-deficient hair cells.^384^^,^^812^ Therefore, this multimodal methodology bridges molecular-scale force application with systems-level physiological outputs, offering high resolution for dissecting aGPCR roles in mechanobiology.

### E Receptor dissociation

In most aGPCRs, the GAIN domain is autoproteolytically active, although, for any given aGPCR, self-cleavage may be contextual depending on cell type^25^^,^^185^^,^^306^^,^^813^ or other factors such as receptor glycosylation.^185^ The autoproteolytic cleavage of aGPCRs and thus receptor dissociation can be suppressed by point mutations immediately adjacent to the GPS^26^ or within the intradomain environment of the cleavage site,^52^ For example, mutations of a highly conserved histidine at the −2 position of the GPS abolishes cleavage and dissociation.^26^^,^^32^^,^^281^^,^^294^^,^^296^^,^^379^

Detecting NTF-CTF dissociation events (Figs. 4 and 6), especially at high spatial and/or temporal resolution, is important for understanding aGPCR function but technically challenging. A biochemical approach used to characterize aGPCR dissociation involves affinity purification/immunoprecipitation of receptors using NTF- or CTF-specific antibodies or epitope tags to assess whether the 2 fragments remain noncovalently bound or have dissociated. As was demonstrated for D1 using this approach and subcellular fractionation,^226^ cleavage occurs in the ER, but the NTF and CTF remain noncovalently bound until they reach the plasma membrane. Once localized at the plasma membrane dissociation can take place.

A recent advance is the development of an NRS system, which enables transcriptional detection and quantification of NTF dissociation from any given aGPCR in cell culture, and at cellular resolution, also in vivo (Fig. 8).^153^^,^^723^ Its utility was demonstrated using NRS reporters for the *Drosophila* receptors Cirl, Mayo, and Ketchup. In particular, analysis of Cirl-NTF release conditions in the developing brain revealed important biological functions of Cirl dissociation at the interface between glial and neural progenitor cells.^153^

*Critical synopsis and outlook: The adaptation of emerging technologies for the study of molecular, cellular, physiological, and pathophysiological aspects has significantly improved the analyses of aGPCR properties. Specific focus is currently warranted to emulate the native environment, in which aGPCR natively receive and respond to their adequate stimuli, in in vitro assays. This requires different approaches than the pharmacological and cell biological analyses of non-aGPCRs and is hampered by the lack of detailed information on the ligand spectrum of individual homologs, the properties of mechanical stimuli they are activated by, and the potential crosstalk with other agonistic conditions such as steroid agonists. To complete our knowledge on these determinants of aGPCR signals, an increasing number of biophysical approaches, such as the ones pioneered for the investigation of motor proteins, adhesion molecules, and other mechanoresponsive molecules, are required and need to be adapted to aGPCR questions. Also, structural studies that aim at characterizing full-length aGPCR in their native tissue environment including cryo-ET analyses, and molecular modeling techniques to grasp the dynamics of aGPCR systems without and with interactors are needed from here on out to continue the successful characterization of aGPCR-dependent signals.*

## XVI Perspectives

The decade that has passed since the last comprehensive review on aGPCRs published in this journal^1^ has witnessed tremendous scientific progress across all aspects of these receptors, including their structure, signaling, biochemistry, and the diverse cell, tissue, and organ functions they regulate, and the consequences of their dysfunction. This progress has been driven by the concerted efforts of the international research community dedicated to aGPCRs, as represented by the Adhesion GPCR Consortium (https://www.adhesiongpcr.org). This collaborative network has played a pivotal role in advancing methodological innovations, standardizing aGPCR nomenclature to ensure precise communication, and fostering interdisciplinary dialog among structural and cellular biologists, pharmacologists, biochemists, physiologists, geneticists, and clinicians. As a result, our mechanistic understanding of aGPCR activation and signaling paradigms has advanced significantly, laying the foundation for a more comprehensive understanding of aGPCR function and paving the way for novel therapeutic approaches targeting these complex receptors. However, many fundamental questions remain unanswered, new scientific challenges are emerging, and the technological toolbox for aGPCR research remains incomplete. Major knowledge gaps include endogenous receptor expression patterns, receptor-specific ligands, context-dependent signaling pathways, isoform-specific functions, and the integration of aGPCR functions within broader cellular and physiological networks. Technological advances, including the development of specific antibodies, protocols for the application of mechanical stimuli, and assays to read and quantify their mechanoresponses, are highly sought after. Addressing these scientific and technological challenges will require sustained collaboration, further technological advances in structural and functional assays, both in silico, in vitro, and in vivo development of fragment- and isoform-specific antibodies, and the translation of basic discoveries into clinically relevant strategies. Considering the remarkable achievements to date, aGPCR research stands well positioned to capitalize on these insights, deepen our biological understanding, and ultimately harness the therapeutic potential of aGPCRs in a wide range of diseases in which they play critical roles. We look forward with great anticipation to the advances and insights that the coming decade of aGPCR research will bring.

## Conflict of interests

Dimitris G. Placantonakis holds patents on the therapeutic targeting of ADGRE5 and ADGRD1 in GBM. Marie-Gabrielle Ludwig is an employee of Novartis Pharma AG. Laurent Sabbagh is an employee of Domain Therapeutics. Erwin G. Van Meir is co-founder, CSO, and shareholder of OncoSpherix, Inc. Giselle R. Wiggin is Employee of and shareholder in Nxera Pharma UK Limited. Norbert Sträter and Tobias Langenhan are coinventors of a pending patent covering NTF release sensors for aGPCRs (WO/2022/063915; priority application: EP 3974535; applicant: Leipzig University). David E. Gloriam is a part-time employee and warrant-holder at Kvantify. Benoit Vanhollebeke is shareholder and founder of NeuVasQ Biotechnologies. Stephen C. Blacklow is on the board of directors for the nonprofit Revson Foundation and nonprofit Institute for Protein Innovation, is on the scientific advisory board for Erasca, Inc. and MPM Capital, is head of the scientific advisory board with equity in Odyssey Therapeutics, and is a consultant for Scorpion Therapeutics.
